# Parsimonious Model of Vascular Patterning Links Transverse Hormone Fluxes to Lateral Root Initiation: Auxin Leads the Way, while Cytokinin Levels Out

**DOI:** 10.1371/journal.pcbi.1004450

**Published:** 2015-10-27

**Authors:** Sedeer el-Showk, Hanna Help-Rinta-Rahko, Tiina Blomster, Riccardo Siligato, Athanasius F. M. Marée, Ari Pekka Mähönen, Verônica A. Grieneisen

**Affiliations:** 1 Institute of Biotechnology, University of Helsinki, Helsinki, Finland; 2 Computational and Systems Biology, John Innes Centre, Norwich United Kingdom; 3 Department of Biosciences, Viikki Plant Science Centre, University of Helsinki, Helsinki, Finland; VIB University Gent, BELGIUM

## Abstract

An auxin maximum is positioned along the xylem axis of the *Arabidopsis* root tip. The pattern depends on mutual feedback between auxin and cytokinins mediated by the PIN class of auxin efflux transporters and *AHP6*, an inhibitor of cytokinin signalling. This interaction has been proposed to regulate the size and the position of the hormones’ respective signalling domains and specify distinct boundaries between them. To understand the dynamics of this regulatory network, we implemented a parsimonious computational model of auxin transport that considers hormonal regulation of the auxin transporters within a spatial context, explicitly taking into account cell shape and polarity and the presence of cell walls. Our analysis reveals that an informative spatial pattern in cytokinin levels generated by diffusion is a theoretically unlikely scenario. Furthermore, our model shows that such a pattern is not required for correct and robust auxin patterning. Instead, auxin-dependent modifications of cytokinin response, rather than variations in cytokinin levels, allow for the necessary feedbacks, which can amplify and stabilise the auxin maximum. Our simulations demonstrate the importance of hormonal regulation of auxin efflux for pattern robustness. While involvement of the PIN proteins in vascular patterning is well established, we predict and experimentally verify a role of *AUX1* and *LAX1/2* auxin influx transporters in this process. Furthermore, we show that polar localisation of PIN1 generates an auxin flux circuit that not only stabilises the accumulation of auxin within the xylem axis, but also provides a mechanism for auxin to accumulate specifically in the xylem-pole pericycle cells, an important early step in lateral root initiation. The model also revealed that pericycle cells on opposite xylem poles compete for auxin accumulation, consistent with the observation that lateral roots are not initiated opposite to each other.

## Author Summary

After moving onto land, plants developed vascular tissues to support their weight and transport water and nutrients. Vascular tissue consists of xylem, which makes up wood, and phloem, which gives rise to the innermost bark. In the model species *Arabidopsis thaliana*, these tissues form in the growing root tip in a radial pattern consisting of a xylem axis and two phloem poles. Xylem is thought to be positioned by negative interactions between two plant hormones, auxin and cytokinins. Cytokinins activate exporters which pump auxin out of cells, while auxin activates a gene which blocks cytokinin response. This leads auxin to accumulate in some cells which become xylem cells. We developed a computational model which includes only the essential processes but allows them to interact in a realistic spatial context. Using this model we show that these interactions can produce the expected auxin pattern even without a pattern in cytokinin distribution, contrary to expectations based on observed patterns in cytokinin signalling. Furthermore, we learned that hormonal regulation fine-tunes the exporters’ activity, and auxin importers play an important role. The regulatory network not only ensures correct formation of the vasculature but may also position root branches on alternating sides of the xylem.

## Introduction

The development of vascular tissue was a critical innovation in the evolutionary history of plants, providing a mechanism for long-distance transport and structural support that was crucial for their colonisation of land. In order to fulfil these functions, vascular tissues must be appropriately positioned to ensure the continuity of the vascular strands, forming a transport and communication network between distant tissues and organs. The vascular tissues also comprise wood, which is a critical global resource. Improving our understanding of vascular development in plants is therefore an important challenge with yields for both fundamental and applied biology.

**Fig 1:**
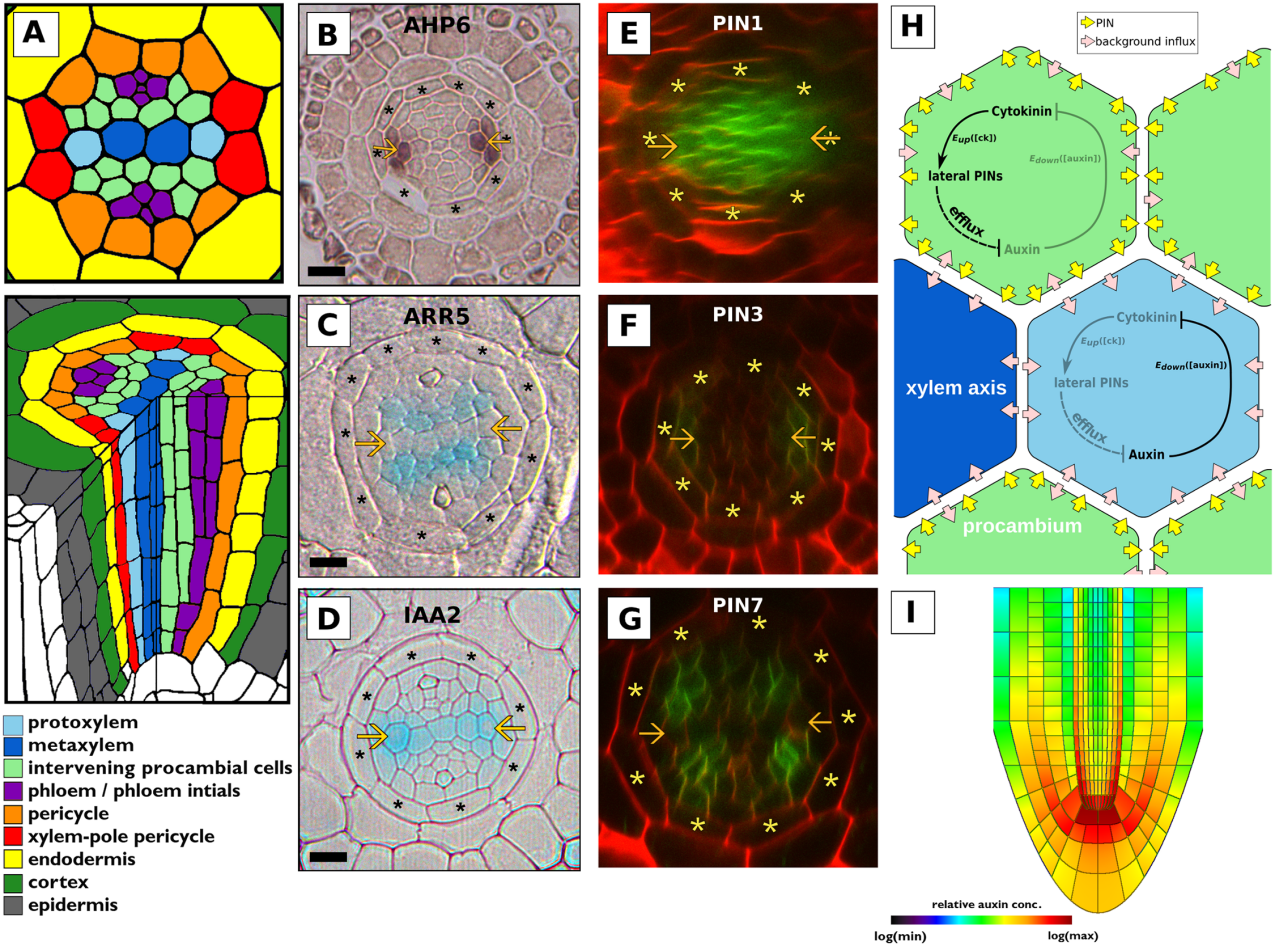
A mutually inhibitory interaction between auxin and cytokinin positions the auxin signalling maximum along the xylem axis. (A) A schematic of the *Arabidopsis* root tip. (B) Localisation of *AHP6* mRNA via *in situ* hybridization. (C) The cytokinin signalling domain reported by *pARR5::GUS*. (D) The auxin signalling domain reported by *pIAA2::GUS*. (E–G) Expression patterns of (E) *PIN1*; (F) *PIN3*; and (G) *PIN7* fused with GFP. (H) Schematic of the parsimonious model showing the mutually inhibitory interactions between auxin and cytokinin which are proposed to generate complementary signalling domains. The network is active in all cells but shown in only two, representative of the procambium/pericycle and the xylem axis; the faint elements are proposed to be downregulated in the corresponding domain. (I) Simulation of a longitudinal section of a root shows uneven auxin accumulation throughout the stele, with outer cells (pericycle) accumulating higher levels of auxin (parameter settings and PIN localisation as described in [5]). Arrowheads mark the protoxylem position in cross sections; scale bars represent 10*μm*. In the model schematic, yellow arrows represent PIN transporters and pink arrows represent passive auxin influx.

The precise and consistent layout of the vascular cylinder (stele) of *Arabidopsis thaliana* makes it an ideal system in which to study vascular patterning. The *Arabidopsis* stele is comprised of a central xylem axis flanked by intervening procambial cells; these separate the xylem from the phloem poles, which are located perpendicular to the xylem axis (Fig 1A). The xylem axis consists of two cell types, protoxylem and metaxylem; protoxylem cells are positioned at the margins of the axis and mature earlier than the metaxylem cells in the centre [1]. Specification of the protoxylem cells is one of the earliest events in post-embryonic vascular patterning [2] and so has been the focus of attempts to unravel the genetic and hormonal interactions involved in vascular patterning. It has recently been suggested that the correct positioning and specification of protoxylem in the *Arabidopsis* stele depends on a mutually inhibitory interaction between two major classes of plant hormones, auxin and cytokinins [3]. Although recent modelling efforts are consistent with this proposition [4], the relevance and plausibility of an informative spatial pattern in cytokinin levels in this context remains unclear.

Auxin and cytokinins have long been known to interact antagonistically in a wide range of processes in plants [6–8], such as meristem maintenance in the shoot [9] and root [10], control of axillary branching [11, 12], and lateral root initiation [13]. Recently, researchers have begun to elucidate the molecular and mechanistic bases of this antagonism. Auxin and cytokinins are known to influence each other’s synthesis [14–16], metabolism [16, 17] and signalling and response machinery [3, 9, 10, 18–20]. Moreover, cytokinin has emerged as a key regulator of auxin transport [3, 10, 21], and this regulation has been shown to play a central role in protoxylem specification [3]. Auxin transport depends on the activity of molecular transporters; while auxin can either passively permeate into cells or enter them via AUX1/LAX importers, its efflux must be mediated by exporters such as the PIN class of efflux carriers [22, 23]. Auxin may also move symplastically via plasmodesmata [24], and transporters of other families, such as the PGPs [25–28] and NRTs [29, 30], may interact with the PINs or otherwise modulate auxin movement. The PIN proteins, however, are the only auxin exporters known to be polarly localised within cells, and their localisation along specific regions of the plasma membrane has been shown to be an important factor determining the overall auxin distribution and flux pattern [31–33]. In the current work, we investigate how cytokinin signalling modifies the auxin distribution and flux via the PIN transporters in the *Arabidopsis* root tip and explore the possible functional consequences.

Cytokinin signalling is essential for correct vascular patterning. The stele of *wol* plants, which have a mutation in the cytokinin receptor *CRE1* leading to a severely attenuated cytokinin response, is smaller than in wild type and is composed entirely of protoxylem cells [2, 34]. Cytokinin inhibits the specification of protoxylem; repression of cytokinin signalling at the protoxylem position by *AHP6* (Fig 1B) is required for normal protoxylem specification, and treatment with exogenous cytokinin or a mutation in *AHP6* results in the spread of cytokinin signalling to the protoxylem position and a concomitant loss of protoxylem [35]. A domain of high cytokinin signalling is observed in the procambial cells in cross sections of wild-type roots (Fig 1C, [35]) with a complementary domain of high auxin signalling in the xylem axis (Fig 1D, [3]). Expression of PIN1 and PIN7 overlaps with the domain of cytokinin signalling (Fig 1E and 1G), which has been shown to regulate the expression of *PIN7* and the localisation of PIN1 on lateral plasma membranes in the procambium [3]. Coupled with the specific expression pattern of PIN3 (Fig 1F), this is proposed to result in transport of auxin away from the regions with high cytokinin signalling and towards the xylem axis, where it accumulates and promotes xylem identity. Auxin accumulation also activates *AHP6* expression at the protoxylem position [3]. Given that AHP6 in turn inhibits cytokinin signalling [35], the data suggest that a feedback loop involving hormone transport dynamics and effectively mutually inhibitory interactions provides a mechanism for positioning the xylem axis in *Arabidopsis* (Fig 1H, [3]). While *AHP6* expression along the xylem axis is regulated by the HD-ZIP III transcription factors [36], the mechanism by which cytokinin signalling is excluded from the metaxylem remains unclear, though the weak phenotype of the *ahp6* mutant suggests that a redundant partner of *AHP6* may be active in the metaxylem.

It is clear that cytokinin signalling plays an important role in vascular development. The patterns in cytokinin signalling, such as those observed via *pARR5::GUS*, are a combined consequence of both the spatial distribution in the levels of cytokinin and the strength with which individual cells respond to those levels of cytokinin. Since it is known that hormonal cross-talk can regulate the cellular cytokinin signalling and response machinery, it remains unclear to what extent patterns in cytokinin signalling reflect actual underlying distributions in cytokinin levels rather than reflecting patterns in the response machinery and hence indirectly the auxin pattern in the tissue, since this response depends on auxin. In the most extreme scenario, cytokinin could even be spatially homogeneous, with variations in signalling resulting solely from the expression patterns of the receptors and auxin-mediated modifications of the downstream effects of cytokinin. To shed light on the auxin dynamics guiding vascular patterning and the relative importance of cytokinin levels versus the cytokinin response machinery in this process, we here investigate auxin-cytokinin interactions in a realistic spatial context. Computational modelling provides tools with which to address complex feedbacks such as these and has been applied successfully in similar contexts [31, 37, 38]. Our model identified additional components of the genetic-hormonal network coordinating vascular development and has uncovered a mechanism by which regulated transverse auxin transport increases the robustness of the xylem-axis maximum and, unexpectedly, impinges on lateral root initiation.

## Materials and Methods

### Computational Methods

Simulations were conducted using a grid-based spatial model of auxin and cytokinin movement in which cells occupy an experimentally reasonable area and the cell wall is explicitly included; inputs to the model were cell and tissue geometry, as well as cell polarity. While the model does not attempt to explain the polarity patterns themselves (it assumes them as an initial condition), it does allow us to explore the concentration and flux patterns resulting from the patterns in polarity over the tissue. Our focus is to study how transporter regulation can alter these patterns given the feedbacks through auxin and cytokinin. The model can be considered an extension of a previously published model of longitudinal auxin transport in the *Arabidopsis* root [31]. While the earlier model only considered auxin and the role of transporters in an unregulated fashion, the current work explicitly considers the regulation of transporter activity under the control of auxin and cytokinin. These hormones, in turn, dynamically move within the tissue due to diffusion and membrane transport properties. We strive for a parsimonious model that allows us to gain general insights into the patterning mechanisms while maintaining sufficient proximity to the core players involved to allow our model to be compared and contrasted to mutants and hormone treatments.

For the purposes of our model, we explicitly capture three exporters and one importer under independent control of the regulatory dynamics described in [3] and [39]. The PIN proteins are treated as identical in terms of factors such as affinity or transport efficiency, differing only in how they are regulated and localised. However, an additional weaker PIN is used to represent weak expression of *PIN7* in the phloem position, differing only in having a lower permeability. We do not explicitly model PIN transcription and translation, nor do we include post-translational processes that might impact PIN activity. Instead, we use a simplified approach wherein PIN ‘expression’ in our simulations represents the PIN activity level resulting from all of these processes. Furthermore, an additional, unregulated efflux transporter is included with apolar expression in the outer tissue layers (endodermis, cortex, epidermis), while the AUX1/LAX importers are represented by an influx transporter with apolar localisation in all stele cells. This importer is auxin-upregulated only in the context of lateral root initiation. Finally, our model makes the simplifying assumption of uniform longitudinal flux through the cross section, which we implement by a uniform constant influx as well as a uniform efflux of auxin and cytokinin into and out of the plane of study. Note that within this parsimonious two-dimensional (2D) modelling setting, influx into the cross-sectional plane cannot be distinguished from biosynthesis, nor efflux out of the cross-sectional plane from degradation. All the processes that are included in the model are described hereinafter.

#### Synthesis and degradation of auxin and cytokinin

Biosynthesis and degradation, or alternatively, influx into and efflux out of the 2D plane of study, of auxin and cytokinin occurs in all cells according to the following basic equation:

$$\begin{array}{c} \frac{\partial C}{\partial t}=b-\delta C+\text{transport terms}, \end{array}$$ (1)

where *C* represents the concentration, *b* the biosynthesis rate (or influx into the plane), and *δ* the degradation rate (or efflux out of the plane). When we simulated cytokinin gradients, *b* was confined to specific cell types in order to create a localized source of influx/biosynthesis, as an extreme attempt to generate an informative gradient, while *δ* was set to provide the desired gradient steepness; the value of *b* and *K*_*A*_*cyt*__ (see below) were altered in those simulations to retain consistent average cytokinin signalling levels. Parameter values for the different simulation conditions are provided in Tables 1–3.

**Table 1:** General parameters.

symbol	description	unit	value
Δ*t*	numerical time step	*s* (seconds)	0.1
Δ*x*	numerical space step	*μm* (microns)	0.3
*D*_*auxin*_	auxin diffusion coefficient	*μm* ^2^/*s*	600
*P*_*i*_*bg*__	background auxin influx permeability	*μm*/*s*	5
*P*_*i*_*AUX*__	auxin influx permeability due to active import	*μm*/*s*	20
*P*_*e*_*bg*__	background auxin efflux permeability	*μm*/*s*	1
*P*_*e*_*PIN*__	auxin efflux permeability due to PIN expression	*μm*/*s*	20
*D*_*cyt*_	cytokinin diffusion coefficient	*μm* ^2^/*s*	600

These are the parameters used in all the simulations.

**Table 2:** Signalling parameters.

symbol	description	unit	value
*K*_*A*_*cyt*__	cytokinin concentration at which PIN transport activity is half maximum (for no cytokinin gradient, c.q. steep cytokinin gradient)	*a*.*u*.	10, 0.6
*K*_*A*_*PIN*__	auxin concentration at which PIN transport activity is half maximum	*a*.*u*.	100
*K*_*A*_*AUX*__	auxin concentration at which AUX1 transport activity is half maximum (during lateral root initiation)	*a*.*u*.	100
*n*	Hill coefficient for all signalling-response curves	none	2

Parameters related to hormonal regulation of the transporters. The *K*_*A*_*cyt*__ values give rise to a PIN activity level in the stele of ≈ 70% in the simulations without a cytokinin gradient and ≈ 20–50% in the simulations which generated a steep cytokinin gradient.

**Table 3:** Metabolic parameters.

symbol	description	unit	value
*b*_*auxin*_	auxin biosynthesis rate	$\frac{a.u.}{\left(s)(\mu m^{2}\right)}$	1 × 10^−4^
*δ*_*auxin*_	auxin degradation	*s* ^−1^	1 × 10^−5^
*b*_*cyt*_	cytokinin biosynthesis rate (no gradient; steep gradient)	$\frac{a.u.}{\left(s)(\mu m^{2}\right)}$	1 × 10^−3^; 100 (only in source cells)
*δ*_*cyt*_	cytokinin degradation (no gradient; steep gradient)	*s* ^−1^	1 × 10^−4^; 4

Parameters related to auxin and cytokinin metabolism.

#### Transport and diffusion of auxin and cytokinin

Under default conditions, cytokinin was allowed to diffuse freely within cells, as well as across the plasma membrane and throughout the apoplast, with no change in the diffusion coefficient. As we have not found a published diffusion coefficient for cytokinin, we used the diffusion coefficient of auxin (from [31]), which is a molecule of approximately the same size, composition, and shape. Auxin was allowed to diffuse freely within cells and within the apoplast at a lower rate. Diffusion of both auxin and cytokinin is in accordance with Fick’s law:

$$\begin{array}{c} \vec{J}=-D\vec{\nabla }C. \end{array}$$ (2)

Although auxin can diffuse freely within cells and in the cell walls, the plasma membrane represents a barrier to auxin diffusive flux. There is a small amount of “leakage” across the membrane, taken into account by a background influx and efflux permeability term. However, auxin flux across the plasma membrane occurs primarily via the action of transporters. In the absence of transporters, efflux is severely limited (represented by a low level of leakage), while influx occurs more readily but is still strongly increased by the presence of a transporter. Auxin flux across the plasma membrane is represented in our simulations by the following equations:

$$\begin{array}{c} \vec{J}=\{\begin{array}{cc} -\left(E_{pin}P_{e_{PIN}}\hat{n}\right)C_{in}+\left(E_{aux}P_{i_{AUX}}\hat{n}\right)C_{out}+\left(P_{i_{bg}}\hat{n}\right)C_{out} & \text{for}\;\text{membranes}\;\text{with}\;\text{at}\;\text{least}\;\text{one}\;\text{PIN}\;\text{expressed},\\ -\left(P_{e_{bg}}\hat{n}\right)C_{in}+\left(E_{aux}P_{i_{AUX}}\hat{n}\right)C_{out}+\left(P_{i_{bg}}\hat{n}\right)C_{out} & \text{otherwise}, \end{array} \end{array}$$ (3)

where

$$\begin{array}{c} 0\leq E_{pin/aux}\leq 1, \end{array}$$ (4)

$\hat{n}$ is the inward directed unit vector perpendicular to the plasma membrane, and *C*_*in*_ and *C*_*out*_ represent the auxin concentration immediately adjacent to the membrane at the cytosolic and cell wall side, respectively. *E*_*pin*_ and *E*_*aux*_ represent the relative activity level of the transporters, and *P*_*i*_ and *P*_*e*_ represent the permeability of the membrane to influx and efflux, respectively. The influx transporter is uniformly localised on all plasma membranes of all stele cells; influx permeability is separated into a smaller passive component, *P*_*i*_*bg*__, and a larger active component due to the transporter, *P*_*i*_*AUX*__. In the absence of efflux transporters, the passive efflux permeability *P*_*e*_*bg*__ is used, while membrane elements on which PIN transporters are localised have a higher efflux permeability, *P*_*e*_*PIN*__. When multiple PIN transporters are localised at the same membrane element, their activities are summed.

#### Regulation of transporter expression levels

The expression level of the individual transporters was determined according to a hormonal regulation model based on experimental data. Auxin upregulates *PIN3*[10] and, during lateral root initiation, *AUX1*[40]. In contrast, when calculating the expression levels of *PIN1* and *PIN7*, the concentrations of both auxin and cytokinin have to be taken into account [3]. *PIN1* and *PIN7* are upregulated by cytokinin, but simultaneously down-regulated by auxin, because auxin activates *AHP6*, thereby inhibiting the cytokinin response which would upregulate the PINs. The auxin-AHP6-cytokinin-PIN pathway has been simplified in this model as a direct downregulation by auxin of *PIN1* and *PIN7*. These regulatory interactions are represented in our parsimonious model by the following equations:

$$\begin{array}{c} E_{pin/aux}=\{\begin{array}{cc} E_{up}\left(\left[auxin\right]\right) & \text{for}\;PIN3\&(\text{during lateral root initation})\;AUX1,\\ E_{up}\left(\left[cytokinin\right]\right)*E_{down}\left(\left[auxin\right]\right) & \text{for}\;PIN1\&PIN7, \end{array} \end{array}$$ (5)

where *E*_*up*_ and *E*_*down*_ represent the hormonal up- and down-regulation of the auxin transporters in our parsimonious model, captured by simple sigmoidal Hill functions of transporter activity (PINs and AUX1) as a function of the average hormone concentration within the cell (*x*):

$$\begin{array}{c} E_{up}\left(x\right)=\frac{(\frac{x}{K_{A_{i}}}){}^{n}}{(\frac{x}{K_{A_{i}}}){}^{n}+1}, \end{array}$$ (6)

$$\begin{array}{c} E_{down}\left(x\right)=\frac{1}{{\left(\frac{x}{K_{A_{i}}}\right)}^{n}+1}. \end{array}$$ (7)

The parameter *K*_*A*_*i*__ represents the hormone level (*x*) that gives rise to a half maximum transport activity. It adopts three possible values, depending on the transporter/hormone combination: *K*_*A*_*AUX*__ for auxin-dependent AUX1 regulation; *K*_*A*_*PIN*__ for auxin-dependent PIN regulation; and *K*_*A*_*cyt*__ for cytokinin-dependent PIN regulation. Please see Table 2 for the values used. Please note that the values of *K*_*A*_*cyt*__ were chosen to give rise to an average PIN activity level of ≈ 70% in the stele (≈ 20–50% in simulations with a steep cytokinin gradient).

#### Cytokinin patterning

Under the default conditions, cytokinin movement in the model occurs solely via diffusion. A cytokinin gradient, such as shown in S7A Fig, was then generated by defining source cells (either the proto- and metaxylem or the phloem, as indicated in the figure captions) and altering the degradation rate to tune the slope of the gradient, as discussed above. In those simulations, a degradation rate of *δ* = 0.24*s* ^−1^ was used, producing a very steep gradient with a characteristic length λ = 11*μ*m and a root mean displacement of $\sqrt{\bar{x^{2}}}=15\mu \text{m}$. We also explored the potential impact of cytokinin confinement by the plasma membrane. While in most of the simulations cytokinin was considered to be able to diffuse freely throughout the cross section, in a subset of simulations the plasma membrane presented a barrier to cytokinin movement, implemented through a bidirectional membrane permeability for cytokinin, *P*_*cyt*_, which we varied. Cytokinin flux across the plasma membrane was then given by:

$$\begin{array}{c} \vec{J}=-\left(P_{cyt}\hat{n}\right)C_{in}+\left(P_{cyt}\hat{n}\right)C_{out}\;. \end{array}$$ (8)

Furthermore, these simulations included a separate and lower diffusion coefficient for cytokinin in the apoplast, *D*_*cyt*_*apo*__, as was done for auxin in this study and in [39].

#### Auxin-driven cytokinin biosynthesis

To explore the possibility that auxin-driven cytokinin biosynthesis could be capable of generating an informative pattern in cytokinin levels, as recently suggested [41], we also modelled the feedback of the auxin levels on cytokinin biosynthesis. This was done by replacing the cytokinin synthesis parameter *b* (in Eq (1)) by a sigmoidal function of the average auxin concentration within the cell (parameters given in Table 4):

$$\begin{array}{c} b\left(auxin\right)=K_{s}\frac{{\left[auxin\right]}^{m}}{{K_{A_{auxin}}}^{m}+{\left[auxin\right]}^{m}}b_{cyt}. \end{array}$$ (9)

**Table 4:** Auxin-driven cytokinin biosynthesis parameters.

symbol	description	unit	value
*K*_*A*_*auxin*__	auxin concentration at which cytokinin biosynthesis is half maximum	*a*.*u*.	0.01
*K*_*s*_	scaling factor for auxin-driven cytokinin biosynthesis	none	6
*m*	Hill coefficient for auxin-driven cytokinin biosynthesis	none	5

#### *wol* mutant and cytokinin treatment

We implement the *wol* mutant by directly modifying both PIN localization and intensity to closely match our own experimental observations (see S4A–S4C Fig). In addition, the anatomy of the realistic *wol* section is based on microscopy of a *wol* plant in order to account for changes in size and cell number. Likewise, to implement cytokinin treatment, we again directly modified PIN localization and intensity following the experimental observations (S4D–S4F Fig). In both cases, cytokinin response and levels are not changed and the PIN modifications are not an indirect consequence of changes in signalling interactions.

#### Numerically solving the equations

Auxin and cytokinin diffusion, membrane transport, production and decay were calculated numerically on the discretised grid points. This was done by numerically solving in an integrative fashion both the reaction part and the spatial coupling of the above equations using an alternating-implicit direction method [42]. Diffusion takes place when two grid points belong to the same cell or when both are part of the cell wall. Permeability is involved whenever two grid points are separated by a plasma membrane. Production and breakdown take place at each grid point belonging to a cell. As usual, appropriate tests were performed to ensure numerical stability and absence of grid effects.

#### Heat maps

Concentrations of auxin and cytokinin are visually shown within the tissue context in two different manners: either through a linear rainbow heatmap ranging from minimum to maximum concentration values (except for Fig 1I, in which we use a logarithmic colour map), or as a “DR5” colour-scheme inspired by auxin response. The latter also uses a linear map scaled between minimum and maximum values, but is designed to use colour intensity to emphasize the regions with high concentrations. Transporter activity is shown using this same colour scheme, now representing the transporter activity level on a per cell basis. The colour bars are shown within the figures or referred to within the captions.

### Experimental Materials and Methods

#### Plant material and histology

Surface-sterilized seeds were stratified for 2 days at 4°C before germination; anatomical and histological analyses were carried out on 5-day-old seedlings grown under long-day conditions. Histological analysis of GUS expression was performed as described in [2, 3]. The *aux1-21, aux1-22, lax1*, *lax2*, *aux1lax1lax2DR5::GFP*, *DR5rev::GFP* and *AUX1/LAX-VENUS-YFP* lines have been previously described [43–49].

#### Protoxylem visualisation

Protoxylem was visualised by clearing roots in an 8:3:1 solution of chloral hydrate: distilled water: glycerol [50] and visualised using Nomarski optics on a Leica DMLB 100T. For cytokinin treatments, plants were germinated on 10 nM 6-benzylaminopurine (Sigma Aldrich).

#### Confocal microscopy

The TCS::GFP plants were grown on 0.5X MS, 1% sucrose, 1% Phytagel medium at pH 5.7 for 4–5 days and induced with 1 μM trans-zeatin in liquid media (1% sucrose, 0.5 MS, pH 5.7) for 2 h, 6 h and overnight. The controls were transferred to a liquid medium without cytokinin for 24 hours. Wild type and *aux1lax1lax2* plants expressing DR5::GFP were grown on 0.5X MS, 1% sucrose, 1% Phytagel medium at pH 5.7 for 5 days before imaging.

The laser intensity and gain settings were adjusted to not saturate the signal in control roots in order to see a reduction or change in pattern in the triple mutants and an increase in the TCS::GFP signal in the cytokinin movement experiment. Imaging was done on Leica SP5 II HCS A, GFP settings, with a 488 argon laser for both experiments.

The TCS::GFP images were analysed with imageJ. Intensity values were extracted from a rectangle 48 μm in height bounded by the epidermis on the upper left and the cell wall between the QC and vascular initials at the lower edge. These were then averaged in Excel and plotted as a scatter graph with error bars indicating the 95% confidence interval.

#### Quantitative RT-PCR

Col-0 seeds were plated on 0.5X MS growth media and grown in a Sanyo growth cabinet for 5 days. Approximately 50 seedlings were transferred to liquid 0.5X MS growth media for either mock (DMSO) or IAA (1 μM) treatments and incubated with shaking. After 0.5 h and 2 h time points 2 mm root tip segments were harvested into liquid N. 100 ng of total RNA isolated with Thermo Scientific GeneJET Plant RNA Purification Kit was DNase-treated and oligodT-primed cDNA synthesis was performed with Revert Aid Premium Reverse Transcriptase (Thermo Scientific). Diluted cDNA was used as a template in qPCR with HOT FIREPol EvaGreen qPCR Mix Plus (no ROX) (Solis BioDyne) and the primers listed in Table 5. The Bio-Rad CFX384 was used with one cycle 95°C for 15 minutes, 50 cycles each consisting of 95°C for 10 s, 60°C for 10 s and 72°C for 30 s, one cycle 95°C for 10 s followed by melt curve analysis. Data was normalised to *ACT2* expression and relative expression calculated with the comparative CT method. Statistical significance was analysed using R packages nlme and gmodels by fitting a linear mixed model with a fixed effect for sample and a random effect for the biological repeat. The model contrasts were calculated and resulting p-values were adjusted for multiple comparisons. The experiment was performed in quadruplicate.

**Table 5:** Quantitative RT-PCR primers.

AGI code	Primer	Sequence
AT2G38120	AUX1_qPCR_F	CGCTCACATGCTCACTTACC
AT2G38120	AUX1_qPCR_R	GCATAAAGAACGGTGGCTTC
AT5G01240	LAX1_qPCR_F	CAAAAACCACGTTATTCAATGG
AT5G01240	LAX1_qPCR_R	CCGCTGCTTTCCAGTACG
AT2G21050	LAX2_qPCR_F	AATCTAAGCTATCTGACATGTTTTGG
AT2G21050	LAX2_qPCR_R	TGGCAGTGTCAACAGCACTT
AT1G77690	LAX3_qPCR_F	TCACCATTGCTTCACTCCTTC
AT1G77690	LAX3_qPCR_R	AAGCACCATTGTGGTTGGAC
AT3G11260	WOX5C1	GCAGAAACGTCGTAAAATCTCCATT
AT3G11260	WOX5C2	CACCTTCTCTTCCTCTTGACAATCTT
AT3G23030	IAA2 Fq	GAAGAATCTACACCTCCTACCAAAA
AT3G23030	IAA2 Rq	CACGTAGCTCACACTGTTGTTG
AT3G18780	ACT2 qPCR FP	ACATTGTGCTCAGTGGTGGA
AT3G18780	ACT2 qPCR RP	CTGAGGGAAGCAAGAATGGA

## Results

In order to investigate the regulatory network discussed above, we implemented its dynamics in a spatially explicit computational model of auxin transport within a cross section of the root, taking into account cell shape, polarity of transporters and the presence of cell walls. In addition to cytokinin regulation of *PIN1* and *PIN7*[3], we also included auxin upregulation of *PIN3*[10] and, during lateral root initiation, of auxin importers such as *AUX1*[40]. While we present some of our results using a rainbow-coloured linear heatmap to display the auxin concentration, it is often useful to highlight the auxin pattern in a manner which can be more readily compared with experimental data. We therefore also use a blue heatmap ranging from the minimum to maximum auxin concentration with an intensity curve designed to be similar to the more familiar output of the auxin reporter *DR5*. (See Materials and Methods for further details.)

### Lateral patterning of the PINs is necessary

The auxin distribution in the root can be predicted through simulations of auxin transport dynamics which take into account both transporter localisation and tissue layout [31]. Simulations based on realistic longitudinal sections of a root using a typical structured polarity pattern (for details, see [5]) predict both a longitudinal and a radial gradient of auxin within the vascular cylinder, with the highest levels found in the marginal cell file, which represents the pericycle (Fig 1I). A naïve rotation of this pattern around the central axis of the root would yield a stele with higher auxin signalling in all pericycle cells. This is clearly inconsistent with the observed pattern of auxin signalling, which is radially asymmetric (Fig 1D, [3]). Models which analyse auxin dynamics within a longitudinal cross-section ignore transverse PIN localisation, which includes tangential PIN localisation (i.e., clockwise and counterclockwise PIN localisation within a transverse cross-section) and radial asymmetries in inward and outward PIN localisation. However, the clear pattern observed in transverse cross-sections suggests that these elements may be important for the positioning of auxin along the transverse axis. To evaluate the role of the fluxes generated by transverse and radial PIN localisations, we implemented a computational model of auxin transport within transverse root sections. The flow of auxin and cytokinin into the 2D plane of observation (i.e. the root’s cross section) was modelled by assuming a constant and homogeneously distributed influx of the hormones into the plane, while the hormones’ efflux out of the plane was emulated through an effective homogeneous decay rate. Given that we focus here on studying the contribution to patterning by the radial and transversal fluxes mediated by transporters and their regulation, we did not assume any prepatterns in hormone entry or exit along the longitudinal axis, unless specified otherwise. (See Material and Methods for equations and simulation details.)

**Fig 2:**
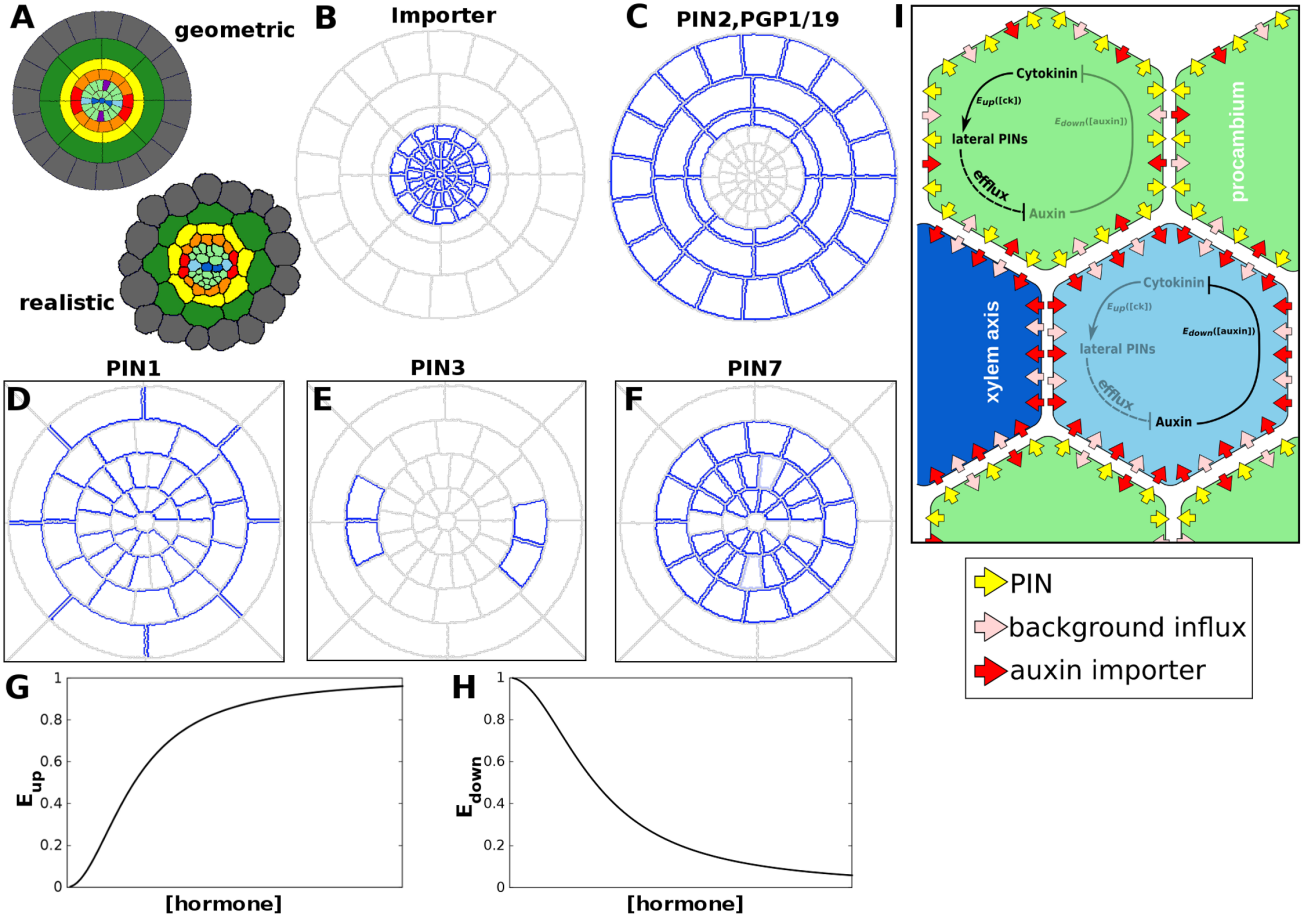
Expression and regulation of the auxin transporters. (A) Geometric and realistic cross sections used for the simulations. Cells were assigned a “cell type” (shown according to the colour code in Fig 1A) that determined the type and localisation of auxin transporters, in line with experimental data. (B) Localisation of the influx transporter. (C) Localisation of the efflux transporter in the outer layers. (D–F) localisation of PIN1, PIN3 and PIN7 in the stele of simulated roots, in accordance with experimental observations [3]. Membranes marked in blue have the potential to express a transporter; lighter colour indicates a lower maximum expression level. (G, H) Response curves for hormonal (G) upregulation and (H) downregulation of the auxin transporters. The expression of *PIN3*, which is simply upregulated by auxin, is *E*_*up*_([*auxin*]); since *PIN1* and *PIN7* are upregulated by cytokinin and repressed by auxin (via *AHP6*), their expression level is calculated as *E*_*up*_([*cytokinin*])*E_*down*_([*auxin*]). (I) A schematic of the model used in the simulations.

We conducted simulations with two different layouts, a ‘realistic’ cross-section that was generated through segmentation of an experimentally obtained confocal cross-section of the root, and a simplified, algorithmically generated ‘geometric’ cross-section (Fig 2A) to allow for a more general analysis of the dynamics. Our model takes into account the spatial structure of cells and the presence of cell walls as well as their organisation within a tissue. This is important because diffusion of the hormones occurs both within the cells and within the cell walls. In the case of auxin, diffusion is not allowed to occur over the plasma membranes; instead, known and regulated influx and efflux permeability rates over the plasma membrane are used. By contrast, cytokinin is allowed to diffuse unrestrictedly through the tissue, unless otherwise specified. An important feature of the model is that the PIN levels within a cell dictate the permeability rate of the auxin efflux through its plasma membrane, assuming a linear relationship between plasma-membrane localised functional PIN and PIN expression. Likewise, the presence of influx carriers, and their expression level, when regulated, determines the influx. Increasing transporter activity leads to a linear, non-saturating permeability increase in the appropriate direction (see Material and Methods).

Our spatial model explores the concentration and flux patterns resulting from underlying polarity patterns in the transporters, which we take as an initial condition to the model. For the purposes of our parsimonious model, PIN proteins are treated as identical in terms of factors such as affinity and transport efficiency, differing only in how they are regulated and localised. However, an additional weaker PIN was used to represent weak expression of *PIN7* in the phloem position, differing only in having a lower permeability rate. Furthermore, an additional efflux transporter with apolar localisation is included in the endodermis, cortex, and epidermis, to represent the reported expression and localisation of *PIN2*[51], *PGP1* and *PGP19*[26] in the outer tissue layers. Localisation of the AUX1/LAX importers is always apolar.

**Fig 3:**
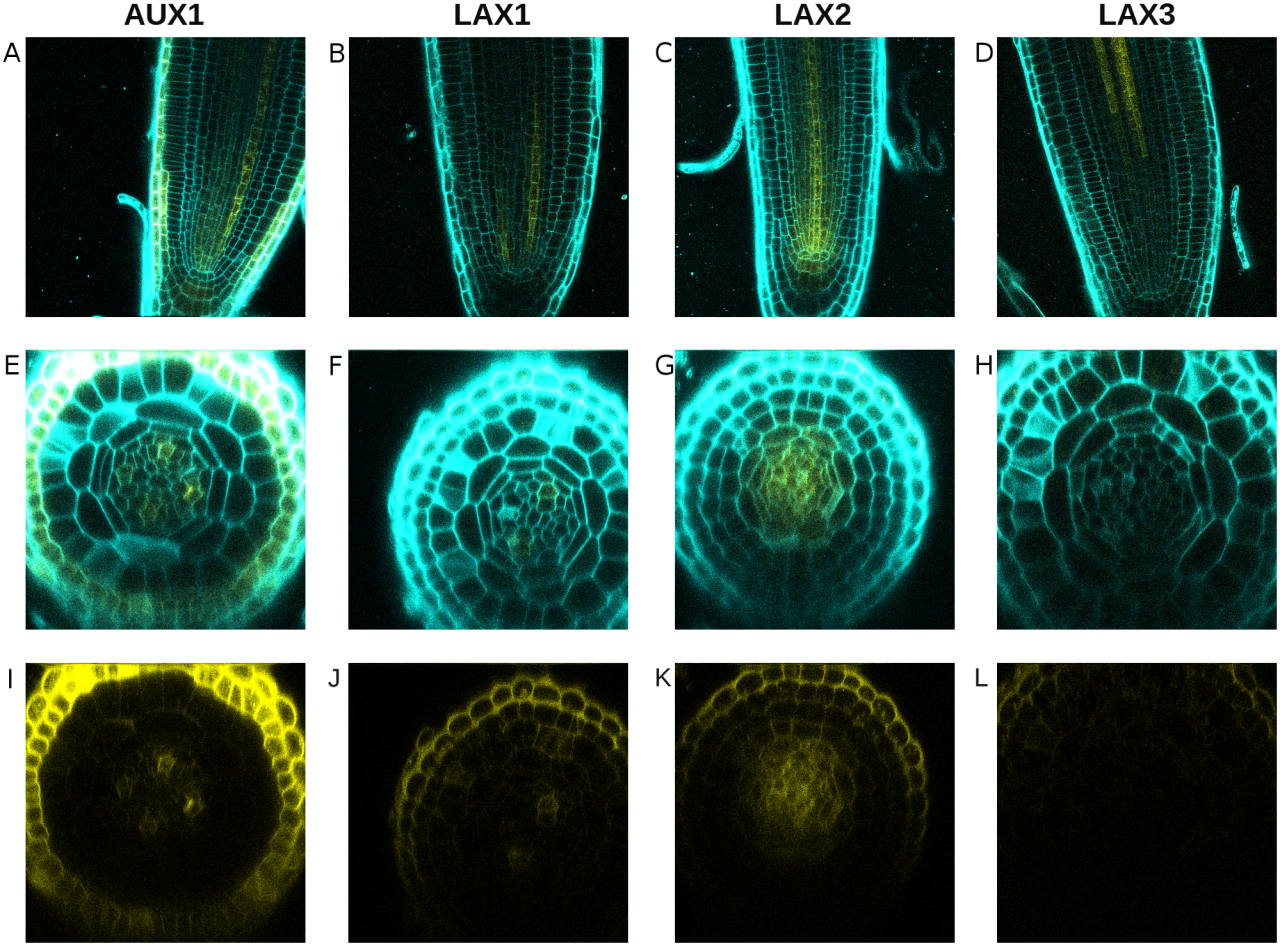
Expression pattern of the auxin importers. (A–D) Longitudinal sections showing (A) *AUX1-VENUS-YFP*, (B) *LAX1-VENUS-YFP*, (C) *LAX2-VENUS-YFP*, and (D) *LAX3-VENUS-YFP* expression in the root tip. (E–K) Transverse sections with colour channels merged (E–H) and only the yellow channel (I–L) showing (E, I) *AUX1*, (F, J) *LAX1*, (G, K) *LA*X2, and (H, L) *LAX3* expression in the root tip. Propidium iodide staining is shown in cyan.

This template, and its underlying elements and formulations, lends itself readily to study how transporter regulation, due to the auxin-cytokinin feedback interactions explained above, can alter hormonal patterns by modifying the permeability contributions of the transporters. Specifically, the current work captures three exporters and one importer under independent control, taking into account the regulatory loop described in [3]. The model therefore includes hormonal regulation of the levels of the auxin transporters *PIN1*, *PIN3* and *PIN7*, which are positioned according to experimental observations (Fig 2D–2F, [3]), and, in certain simulations related to lateral root initiation, of an auxin importer positioned in all stele cells (Fig 2B, see discussion below). Transporter efflux (and, during lateral root initiation, influx) contributions are altered by the hormones on a cellular level, using the average concentration of auxin and cytokinin in each cell (Fig 2I). Simple sigmoidal response curves (Hill-functions) are used, either in an activating or in an inhibitory fashion (Fig 2G and 2H). (See Material and Methods for equations and parameters.) Auxin upregulates *PIN3*[10] and, during lateral root initiation, *AUX1*[40]. By contrast, when calculating the expression levels of *PIN1* and *PIN7*, the concentration of both auxin and cytokinin have to be taken into account [3] in a multiplicative fashion. Note that cytokinin is not transported polarly. We begin with simulations in which cytokinin is considered to influx into and efflux out of all cells in the cross-sectional plane equally. *PIN1* and *PIN7* are upregulated by cytokinin, a process which is simultaneously down-regulated by auxin, due to *AHP6* activation, which inhibits PIN upregulation by cytokinin. Our model incorporates this as a direct inhibition by auxin of the *PIN1* and *PIN7* upregulation by cytokinin (see Eq (5) and further). Note that in order to achieve a parsimonious model, the exporter in the epidermis, cortex and endodermis is not regulated (Fig 2C).

**Fig 4:**
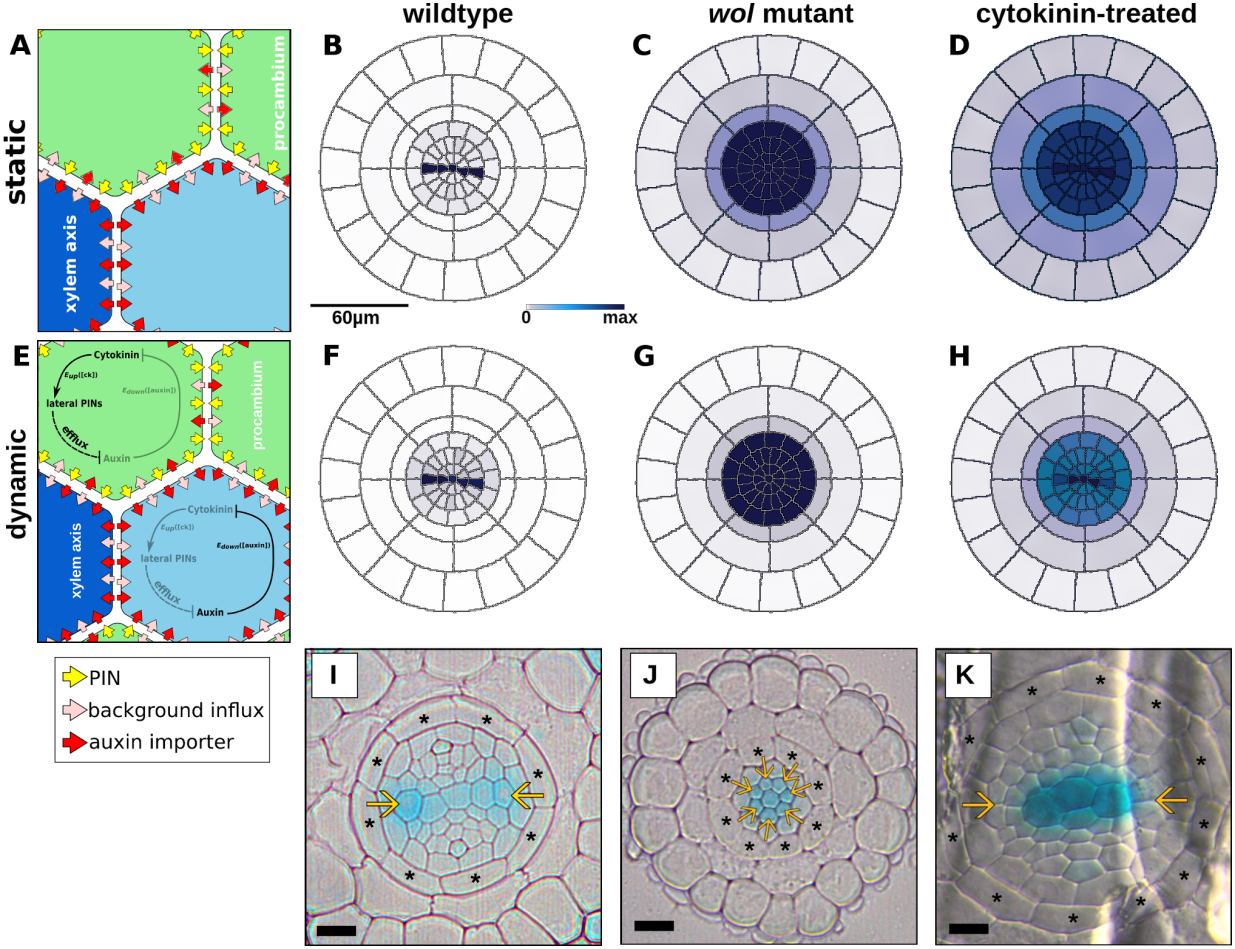
Regulation of the transporters required for the correct auxin pattern. (A–D) The ‘DR5-like’ output of (A) static simulations with unregulated PIN expression in (B) wild type; (C) *wol*; or (D) cytokinin-treated roots. (E–G) The ‘DR5-like’ output of (E) ‘dynamic’ simulations, in which the PINs are regulated, in (F) wild type; (G) *wol*; or (H) cytokinin-treated roots. (I–K) The pattern of the auxin signalling reporter IAA2::GUS in (I) wild type; (J) *wol*; and (K) cytokinin-treated roots. Scale bars in cross sections represent 10*μ*m. In the schematics, yellow arrows represent PIN transporters, pink arrows represent auxin influx via diffusive permeability, and red arrows represent an auxin influx transporter.

Our simulations include a non-specified, generalised auxin importer to represent the activity of the AUX1/LAX importers. AUX1, LAX1, and LAX2 are expressed in the stele near the QC, while LAX3 is not (Fig 3, [47, 48]); taken together, AUX1, LAX1, and LAX2 expression includes the entire stele. Based on these observations, we positioned the auxin importer in our simulations throughout the stele (Fig 2B) to represent the sum of AUX1, LAX1 and LAX2 activity. While *AUX1*, the best characterised auxin importer in *Arabidopsis,* was reported to be upregulated by auxin in roots based on a microarray experiment [40], later observations found no change in the expression of an *AUX1* marker line following auxin treatment [48], raising doubts regarding the generality of auxin-regulation of the auxin importers. We therefore investigated the regulation of the importers in this developmental context by conducting a qRT-PCR on 2 mm long sections of *Arabidopsis* root tips treated with 1 *μ*M indole-3-acetic acid (IAA). Expression of *AUX1*, *LAX2*, and *LAX3* showed a slight but significant increase in response to auxin (S1 Fig). However, the increase was relatively mild compared with genes that are known to be auxin-responsive, such as *IAA2*, *WOX5* (S1 Fig) or *AHP6*[3]. We therefore chose to not include regulation of the auxin importer level in our model in general, even though the qRT-PCR results do not rule out the possibility of indirect auxin regulation of the importers or cell-specific changes in response to auxin. We have also conducted simulations in which the importer is regulated by auxin and found no significant differences in the results (compare S2 Fig and Fig 4F–4H). The observation that *AUX1* is induced by auxin during lateral root formation [39, 40] suggests that this is at least the case in more mature tissues. We therefore do include this regulation when specifically analysing the process of lateral root initiation.

### Regulation of PIN expression is necessary to generate observed auxin patterns

To assess the importance of hormonal regulation in this context, we first considered the impact of a transverse pattern in transporter positioning without including the regulatory network. To do so, we conducted simulations in which the PINs were not hormonally regulated and were therefore constantly expressed at full strength (Fig 4A), which we refer to hereafter as ‘static’ simulations. In both the geometric (Fig 4) and realistic (S3 Fig) cross-sections of wild-type roots, static simulations reproduced auxin patterns similar to those observed in experiments (Fig 4B and 4I; S3A and S3D Fig). This suggests that the known transporter localisation is sufficient to generate appropriate wild-type auxin patterns within the different layouts. However, the simulated and experimentally observed auxin patterns did not match when cytokinin-deficient (*wol* mutants) or cytokinin-treated roots were considered, even though in the model the PINs were again positioned according to their experimentally observed subcellular localisation (S4 Fig) in these modified contexts. Note that while localisation of the PINs was altered (according to observations reported in [3]) in the *in silico* cytokinin treatment and *wol* mutant, cytokinin levels and response were not changed. The auxin pattern in the static simulations of *wol* (Fig 4C, S3B Fig) was broader than the observed pattern (Fig 4J, S3E Fig), with auxin accumulating in the endodermis as well as in the stele. Likewise, static simulations of cytokinin-treated roots (Fig 4D, S3C Fig) showed auxin accumulation in a broad domain that corresponds poorly to the reported pattern (Fig 4K, S3F Fig).

We therefore tested what patterns are predicted when we take into account the observations that PIN levels are dynamically regulated by auxin and cytokinins [3, 52]. (For details, see Materials and Methods.) These ‘dynamic’ simulations, in which we included hormonal regulation of the expression level of the PINs (Fig 4E), generated auxin patterns which were more similar to experimental observations in all three conditions, particularly *wol* (compare the dynamic results in Fig 4F–4H with Fig 4I–4K, in contrast to the static results in Fig 4B–4D).

**Fig 5:**
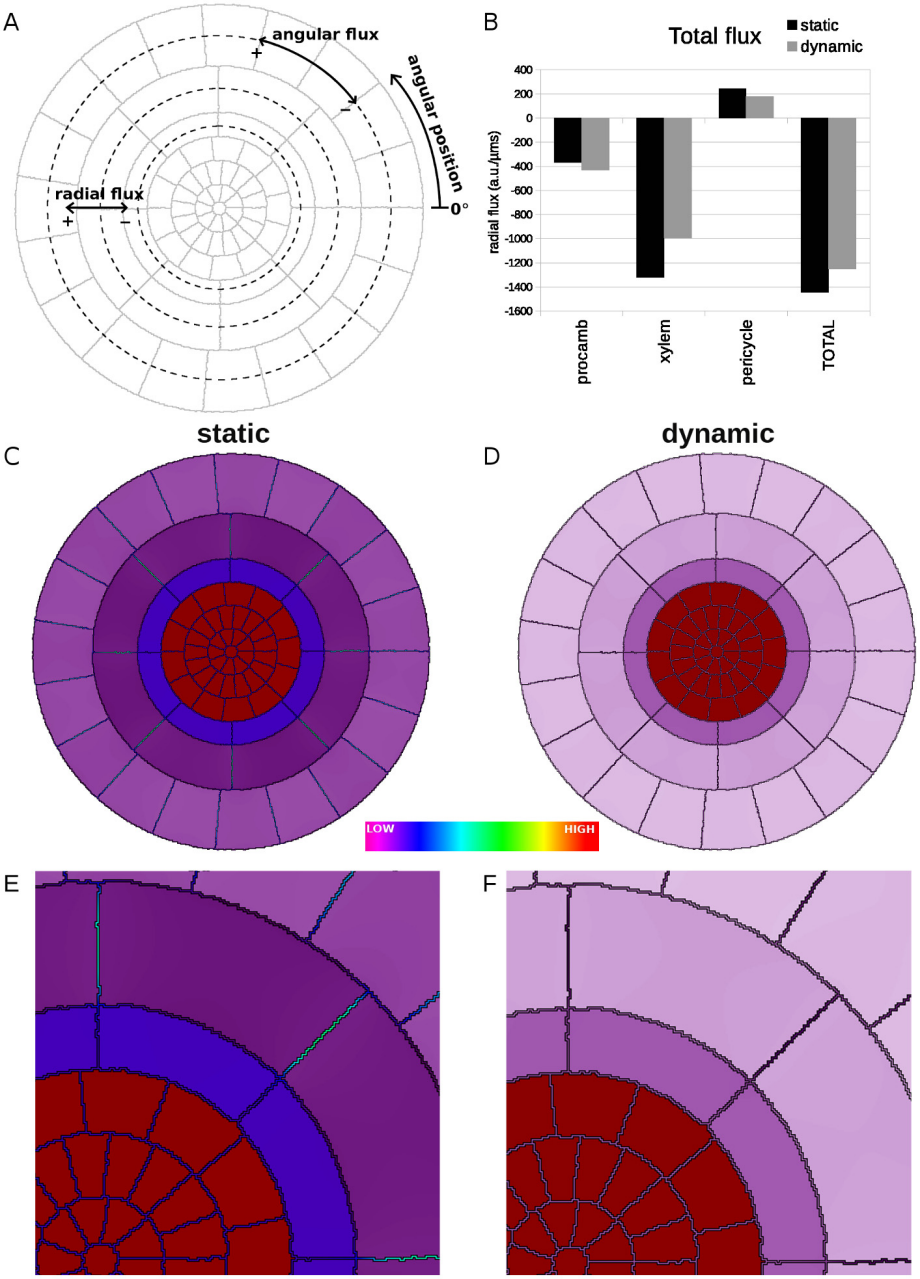
Auxin leaks out of the stele via the apoplast. (A) A diagram showing the orientations used in depicting the fluxes. Angular position is measured counter-clockwise from the line labelled 0°. Negative radial fluxes are inwards; positive are outwards. Negative angular fluxes are clockwise; positive are counter-clockwise. (B) The auxin flux integrated across the cells in each tissue layer. (C, D) Heatmap of auxin levels in the stele of (C) static and (D) dynamic simulations of *wol*. (E, F) Zoomed-in quadrant of the heatmap of the (E) static and (F) dynamic simulations of wol to facilitate comparison of apoplastic auxin levels.

We next tried to understand why the dynamic regulation of PIN levels is important in this patterning process. An inspection of the *in silico* auxin concentration profiles in *wol* with and without regulation suggests that introducing the regulation may promote the stele to effectively act as a sink, thereby generating the experimentally observed auxin pattern. To verify this, we compared the flux pattern of auxin in the static and full dynamics simulations of *wol* roots. We decomposed the flux at each position into a radial (inwards/outwards) and an angular (clockwise/counter-clockwise) component (Fig 5A). The stele as a whole acts as a sink, with a negative (*i.e.*, inwards) net flux in both the static and dynamic simulations (Fig 5B). Unexpectedly, the magnitude of the inward radial flux in the stele is slightly higher in the static simulations. Thus, the difference in the resultant patterns with and without regulation is not due to regulation causing or amplifying the stele to act as a sink.

We therefore explored whether the spatial pattern of the auxin flux might account for the difference between the static and dynamic simulations of *wol.* In general, the flux pattern is quite similar in static and dynamic simulations (S5 Fig). There is no significant difference in the direction of the flux under the two conditions, but the magnitude of the flux is uniformly greater in static simulations, since hormonal regulation reduces the activity of the stelar PINs in the dynamic simulation. As a result, the apoplast accumulates more auxin in the static than in the dynamic simulations, as is seen by a comparison of auxin levels under the two conditions (Fig 5C and 5D, zoomed for easier comparison in Fig 5E and 5F). We propose that the increased auxin in the apoplast in static simulations is available for uptake by the endodermis, cortex, and epidermis, resulting in increased auxin levels in these layers. Diffusion in the apoplast thus provides an avenue for auxin to ‘leak’ out of stele in the static simulations.

### Active auxin import is required for protoxylem specification

It has been shown that the *pin1* mutant presents defects in protoxylem formation while *pin7* does not [3]. We therefore simulated the *pin1* and *pin7* mutants *in silico* by completely removing either PIN1 or PIN7 from the model. Consistent with earlier experimental findings, auxin is severely reduced in the xylem axis in simulations of *pin1* (S6A and S6D Fig) but not *pin7*, although the auxin maximum is diffuse in *pin7* simulations (S6B and S6E Fig).

**Fig 6:**
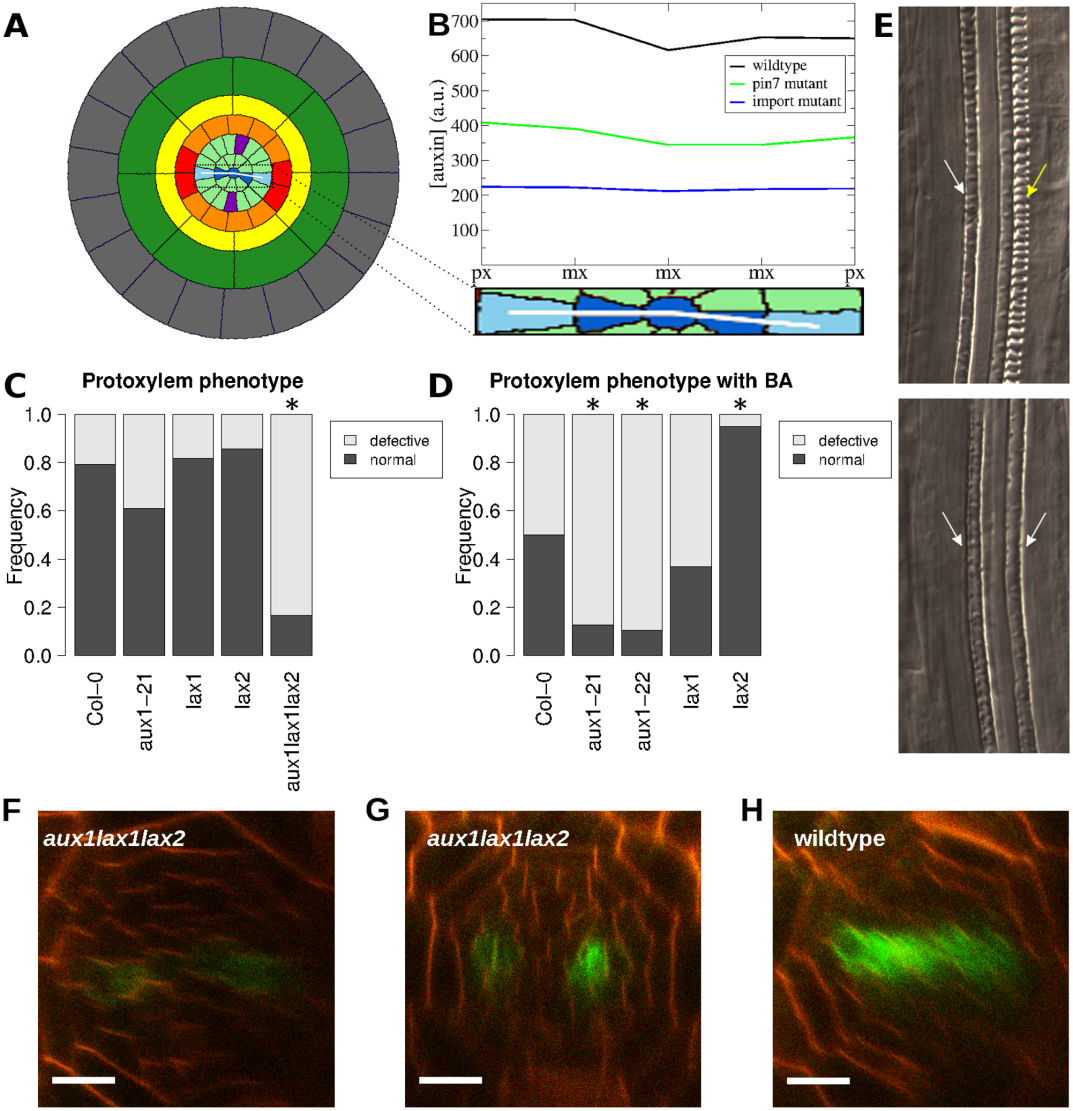
Active auxin import is required for correct protoxylem formation. (A) Auxin concentration was plotted for the cells of the xylem axis, marked with a white line. (B) Plot of the auxin concentration along the xylem axis in simulations of wild type (black), *pin7* (green), and the importer mutant (blue). px = protoxylem; mx = metaxylem. (C, D) Frequency of protoxylem defects in auxin importer mutants (C) without and (D) with a 48 h treatment of 10*n*m 6-benzylaminopurine. ‘Defective’ protoxylem has breaks in one or both strands; ‘normal’ protoxylem is continuous. Asterisks indicate a significant difference from wild type (Col-0). (E) Chloral hydrate cleared *aux1* roots with long gaps in one (top) or both (bottom) protoxylem strands. Yellow arrows indicate protoxylem; white arrows indicate missing protoxylem. (F–H) Expression of *DR5::GFP* in the (F, G) *aux1lax1lax2* mutant and (H) wild type. Scale bar: 10*μ*m.

While the *pin1* and *pin7* mutants have been examined in this context [3], experimental data on the role of the importers is lacking. We therefore investigated the role of the auxin importers in xylem specification. Initial examination of the simulated DR5 pattern in the auxin importer mutant did not reveal major differences from wild type (S6C and S6F Fig). However, an important difference became evident when we plotted the concentration of auxin accumulating in each cell of the xylem axis in simulated roots (Fig 6A and 6B). Despite the spread of the auxin maximum in simulated *pin7* roots and its retention in the axis in the auxin importer mutant, auxin levels in the xylem axis are higher in *pin7* than in the importer mutant (Fig 6B). In our simulations, the xylem axis of the importer mutant has only one-third as much auxin as in the wild-type xylem axis. Since auxin promotes protoxylem specification, we hypothesised that the lower auxin levels in the importer mutant may result in protoxylem phenotypes. We therefore examined the protoxylem of *Arabidopsis* plants with mutations in the auxin importers to test our prediction that these mutants would show defects in protoxylem formation.

Although the *aux1-21, lax1* and *lax2* mutants all exhibited defects in protoxylem formation, the defects were not significantly more frequent than in wild-type plants (Fig 6C). However, roots of the triple mutant *aux1lax1lax2* had a significantly higher frequency of protoxylem defects (multiple pairwise Fisher’s exact test, Hommel-corrected, *p* < 0.01). Consistent with this, expression of the auxin-regulated *DR5rev::GFP* marker was reduced in the *aux1lax1lax2* mutant in 11/14 roots (Fig 6F and 6G). We next conducted a more sensitive assay by germinating the mutants at a cytokinin concentration which normally causes protoxylem defects in about 50% of wild-type plants [35] in order to determine whether a reduction in active auxin import would make these plants more sensitive to cytokinin. Under these conditions, *lax1* plants showed no difference from wild type, but specification of protoxylem in *lax2* was, surprisingly, resistant to cytokinin (Fig 6D; multiple pairwise Fisher’s exact test, Hommel-corrected, *p* = 0.0118). Both *aux1-21* and *aux1-22* also showed significantly more protoxylem defects than wild-type plants (Fig 6D and 6E; multiple pairwise Fisher’s exact test, Hommel-corrected, *p* = 0.0479 and 0.0431, respectively), demonstrating that the robust formation of an auxin signalling maximum in the xylem axis is dependent on active auxin import by AUX1, as predicted by the model.

### Vascular patterning does not require a cytokinin gradient

So far, the model operated without generating a gradient of cytokinin in the stele, leading to the surprising implication that no spatial pattern in cytokinin levels is required for correct and robust auxin patterning in the stele. Nevertheless, two different mechanisms have been proposed by which cytokinin levels could be distributed in a graded fashion. First, rootwards transport of cytokinin via the phloem [52] raises the possibility that a gradient of cytokinin centred around the phloem position might play a role in transverse patterning. While earlier simulations suggest that this gradient is unlikely to act as a source of positional information [4], they were based solely on the connectivity network between cells, rather than the spatial structure of the cells and their connections involving the apoplast. Secondly, a recent modelling effort concluded that local cytokinin biosynthesis in the xylem is crucial for vascular patterning in *Arabidopsis*[41]. In this model, cytokinin synthesis in the xylem axis generates a gradient of cytokinin levels and response which organises the tissue by controlling cell divisions.

In considering the potential importance of certain cells (either phloem or xylem) as sources of cytokinin, we first asked what properties of movement would be required to generate a gradient in the cytokinin levels. The gradient produced by any freely diffusing substance depends on its diffusion coefficient, *D*, and rate of degradation, *δ*; the ratio of the two determines the characteristic length of the gradient, $\lambda =\sqrt{\frac{D}{\delta }},$ which is the distance at which the concentration has dropped to 1e, or about 37% of the concentration at the source. The characteristic length is a good indicator of the spatial range over which a gradient can be informative; its scale should more-or-less correspond to the size of the tissue under consideration. To form a cytokinin gradient with a characteristic length on the order of the *Arabidopsis* stele (50*μ*m) while assuming a diffusion coefficient similar to auxin, a degradation rate of around *δ* = 0.24 *s* ^−1^ is needed. Equivalently, when assuming a degradation rate similar to auxin, a diffusion coefficient of around *D* = 2.5 × 10^−3^ *μm* ^2^/*s* is needed. Both values, however, seem to be very unrealistic and are inconsistent with the known spread of cytokinin. It therefore seems unlikely that an informative gradient of cytokinin can be generated solely via diffusion.

Even though an informative gradient seems unlikely, we nevertheless examined whether such a gradient could play a role in determining the auxin accumulation pattern in our simulations. This was evaluated by allowing cytokinin influx or production to occur solely at either the phloem pole cells or the xylem axis cells. Even with an extremely high cytokinin degradation rate to guarantee a steep gradient centred around the position of the phloem poles (S7A Fig), the auxin pattern in dynamic simulations of wild-type roots (S7B Fig) or *wol* (S7C Fig) did not change noticeably; similar results were obtained when cytokinin influx or biosynthesis was instead restricted to only the xylem axis (S7D and S7E Fig). Consistent with [4], these findings indicate that no spatial pattern in cytokinin distribution is required to generate an auxin maximum in the xylem axis, a spatial pattern in cytokinin levels that is anyway unlikely. However, while no variations in cytokinin levels are required, the simulations do present variations in cytokinin response and signalling (due to the emerging auxin patterning, as each cell independently responds to auxin, which suppresses the cytokinin response machinery).

**Fig 7:**
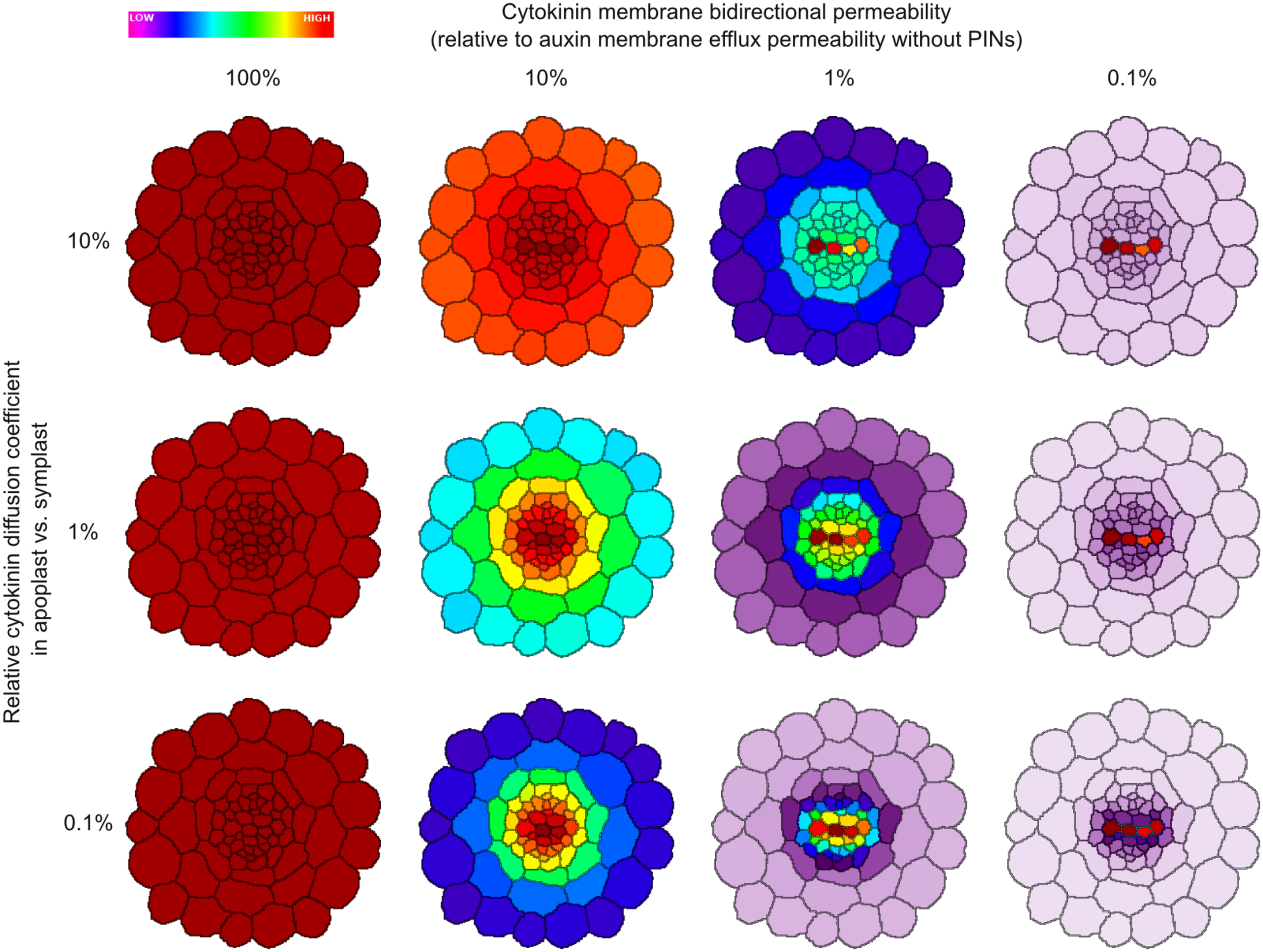
Severely reduced cytokinin movement is required to establish a cytokinin gradient across the *Arabidopsis* root via diffusion from a local biosynthesis source in the xylem axis. To establish a cytokinin gradient without altering the diffusion coefficient of cytokinin, we introduced bidirectional membrane permeability. Decreasing the membrane permeability (moving right within rows) concentrates cytokinin in the source cells (here, the xylem axis), but no morphogen gradient is established, because the diffusion within the apoplast evens out the distribution within the rest of the root. Decreasing the diffusion coefficient (downwards in columns) in the apoplast transforms the step-wise cytokinin distribution pattern into a true gradient. Parameter values are given in Table 6.

While our simulations indicate that a pattern in cytokinin levels is not required, it remains possible that such a pattern is relevant for vascular development. We therefore examined the plausibility of a cytokinin gradient more thoroughly. Given the extreme parameter constraints required to form a significant gradient in cytokinin levels, we considered whether other biophysical properties of the tissue might be able to relax those constraints. For example, a higher diffusion coefficient might be compatible with an informative cytokinin gradient if cytokinin movement is obstructed by plasma membranes. To test this, we ran simulations in which cytokinin movement was affected by plasma membranes, rather than being able to freely pass through them, by introducing a bidirectional cytokinin membrane permeability. To further release the constraints on cytokinin movement required to form a gradient, we considered a lower diffusion coefficient for cytokinin within the apoplast, as is the case for auxin [53]. We varied both parameters to find a regime in which localised cytokinin synthesis could form an informative gradient within the stele via diffusion, as suggested by a recent study [41]. We tested localised cytokinin production in either the xylem axis or the phloem. Remarkably, both apoplastic diffusion and cytokinin membrane permeability must be set to unrealistically low values to generate a gradient. When cytokinin membrane permeability was set equal to the (low) value for auxin efflux when there are no PIN transporters along the plasma membrane, diffusion was still sufficient to generate a very even, almost flat cytokinin distribution (Fig 7 and S8 Fig, leftmost column). Generating a weakly uneven distribution required a membrane permeability of 10% of the auxin value (Fig 7 and S8 Fig, second column), while a permeability of at most 1% was necessary to generate a strongly uneven distribution (Fig 7 and S8 Fig, third and fourth columns). Even under these extreme conditions, the uneven distribution could not be called a ‘morphogen gradient’, because cytokinin was high in the source cells and evenly low elsewhere, without gradation in between (Fig 7 and S8 Fig, top row); the pattern is thus completely cell-autonomous. To generate a weak morphogen gradient, it was necessary to also reduce the diffusion coefficient in the apoplast to 1% of the symplastic value (Fig 7 and S8 Fig, third and fourth column of middle row); a strong gradient required an apoplastic diffusion coefficient only 0.1% of that in the symplast (Fig 7 and S8 Fig, third and fourth column of bottom row). The reason is that the apoplast forms a contiguous space, causing cytokinin levels to even out over the tissue whenever transmembrane transport is very low. The key difference with auxin is that auxin transport can be polarly organised, allowing the slope of gradients to increase many orders of magnitude [54]. So far, there is no indication for such polar transport of cytokinin.

**Table 6:** Cytokinin gradient parameters.

symbol	description	unit	value
*D*_*cyt*_*apo*__	10%, 1%, 0.1% cytokinin diffusion coefficient in apoplast	*μm* ^2^/*s*	60, 6, 0.6
*P*_*cyt*_	100%, 10%, 1%, 0.1% bidirectional cytokinin membrane permeability	*μm*/*s*	1, 0.1, 0.01, 0.0016

Parameters used to study the formation of a cytokinin gradient driven by diffusion.

Until now, we have assumed a prepattern in cytokinin biosynthesis (or influx) in our efforts to explore the feasibility of forming an informative pattern of cytokinin levels. Alternatively, cytokinin biosynthesis could be regulated by auxin, generating a feedback loop that might enable the emergence of a formative gradient in cytokinin levels, as suggested by [55]. To explore this possibility, we implemented auxin-regulated cytokinin biosynthesis. Similar to the results of simulations with prepatterned cytokinin synthesis, auxin-driven cytokinin synthesis only manifested cytokinin patterning under extremely unlikely parameter settings. As soon as the apoplastic cytokinin diffusion coefficient or the cytokinin permeability rate was increased beyond an unreasonably low level, no informative cytokinin gradient was formed (S9 Fig).

The fact that none of our simulation attempts are capable of generating cytokinin patterning within reasonable parameters settings reflects a fundamental property regarding the spatial and temporal scales of morphogen gradients generated through localized production, undirected movement and decay. In such a system, the root mean square displacement, the distance which molecules are typically able to move before breakdown, is calculated as $\sqrt{\bar{x^{2}}}=\sqrt{\frac{2D}{\delta }}=\sqrt{2}\lambda$. Thus, the distance molecules are typically able to move is directly related to the characteristic length, which means that within the context of source-decay mechanism fixing the characteristic length implies fixing the length of communication. Consequently, cytokinin molecules diffusing in a gradient with a characteristic length of 50*μ*m would move, on average, around 71*μ*m before breaking down, independent of the specific choice of effective diffusion or decay [54]. Thus, any choice of parameters to form a cytokinin gradient on this scale implies that cytokinins are able to move less than 100 microns (less than the typical length of a mature root cell), which would preclude a role for it as a signal in longitudinal root-shoot communication, contrary to observations [52, 56].

**Fig 8:**
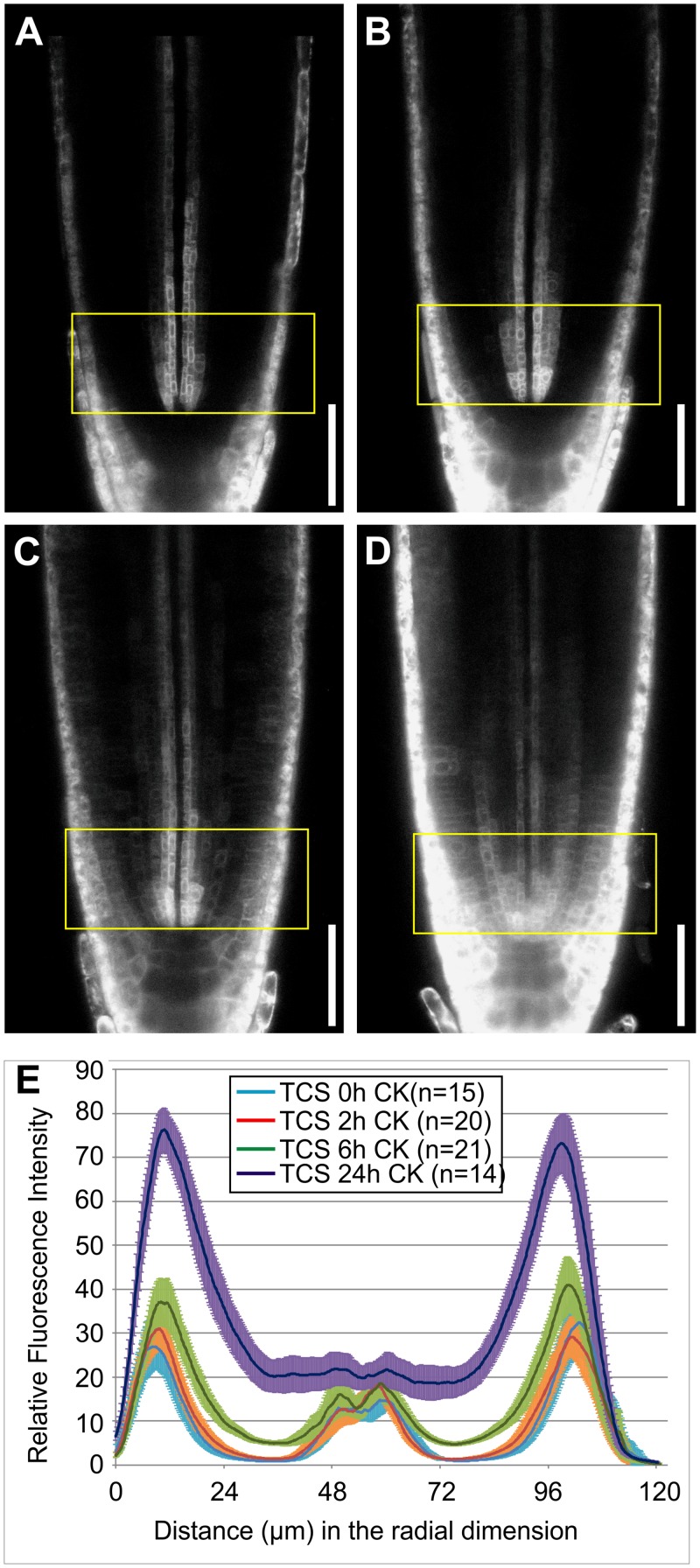
Cytokinin travels rapidly in the *Arabidopsis* root. (A–D) Confocal images showing expression of the fluorescent *TCS::GFP* cytokinin response marker following a (A) 0 h, (B) 2 h, (C) 6 h, and (D) 24 h cytokinin treatment. (E) Graph quantifying the intensity of *TCS::GFP* across the diameter of the *Arabidopsis* root following the cytokinin treatments. The scale bar is 50*μ*m; the yellow boxes highlight the region within 48*μ*m of the QC; error bars mark the 95% confidence interval.

Although severe restrictions on cytokinin movement seem unlikely based on our theoretical treatment and biological considerations of long-distance cytokinin communication, we also experimentally evaluated the validity of this reasoning. To do so, we treated *Arabidopsis* roots with cytokinin and measured the intensity of the fluorescent *TCS::GFP* cytokinin response marker [57] throughout the root cross section. *TCS* intensity increased in response to cytokinin within six hours, with a more marked increase seen following overnight treatment (Fig 8). Importantly, the increase was not confined to an initial response in the outer layers, but was observed at the same timescale throughout the root, implying that cytokinin was able to effectively penetrate into the stele within a timescale of hours, if not minutes. This is inconsistent with the very slow and limited cytokinin movement required in our model, reinforcing the biological implausibility of an informative cytokinin gradient forming on these spatial scales via an effective diffusion-driven process (note that an effective diffusion does not exclude that part of the observed spread might stem from cytokinin-triggered cytokinin biosynthesis).

Given the unlikelihood of an informative cytokinin gradient on the scale of radial patterning within the stele, along with experimental and computational data demonstrating that positional information from xylem- or phloem-derived cytokinin is not essential [4, 52], we conclude that cytokinin most likely evens out, and we therefore continue to operate in the remaining simulations under realistic cytokinin biosynthesis and transport parameter settings, which consequently give rise to an even distribution of cytokinin. Under these settings, the results of simulations with local variations in biosynthesis (either due to a prepattern or due to feedbacks) or with homogeneously distributed biosynthesis become undistinguishable.

### Polar PIN1 localisation generates an auxin circuit

**Fig 9:**
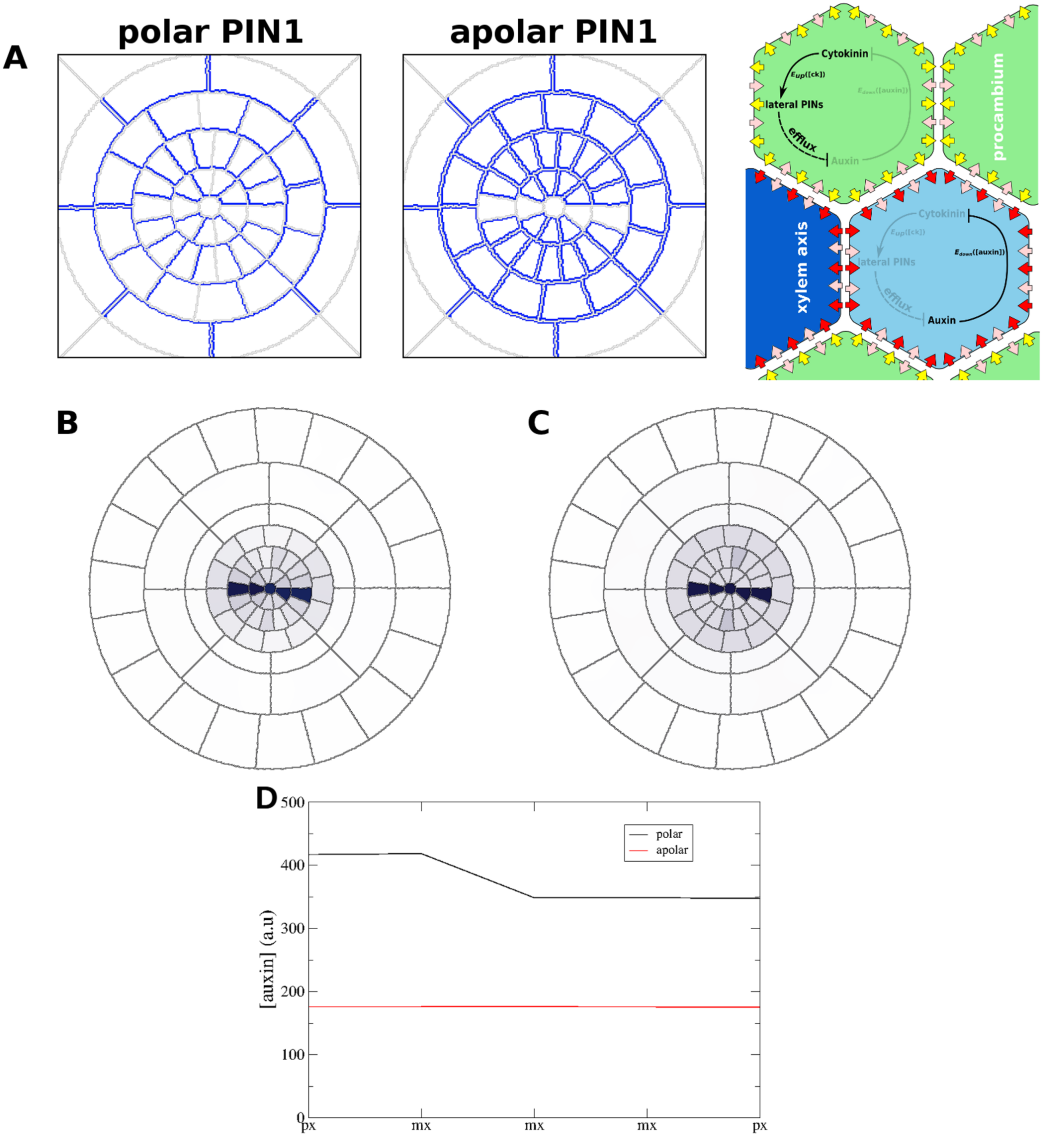
Apolar PIN1 localisation has only a minor effect on auxin accumulation in the xylem axis. (A) Polar (left) and apolar (right) subcellular localisation patterns of PIN1. (B, C) ‘DR5-like’ output showing the auxin pattern along xylem axis in simulations of roots with (B) polar and (C) apolar PIN1 localisation. (D) Plot showing the auxin concentration in cells along the xylem axis of simulations with polar (black) and apolar (red) PIN1.

While the observed subcellular localisation of PIN7 is clearly apolar, immunolocalisations of PIN1::GFP suggest polar localisation [3]. PIN1 is therefore polarly localised within the procambium cells in our simulations, positioned to transport auxin away from the phloem poles and towards the xylem axis (Fig 2D). However, since the experimental data cannot rule out apolar PIN1 localisation, we also investigated the consequences of this possibility in our model (Fig 9A). Apolar PIN1 localisation causes only a small change in the quantity of auxin accumulating in the axis (Fig 9B and 9C), but further examination revealed dramatic differences in the auxin flux pattern throughout the stele.

**Fig 10:**
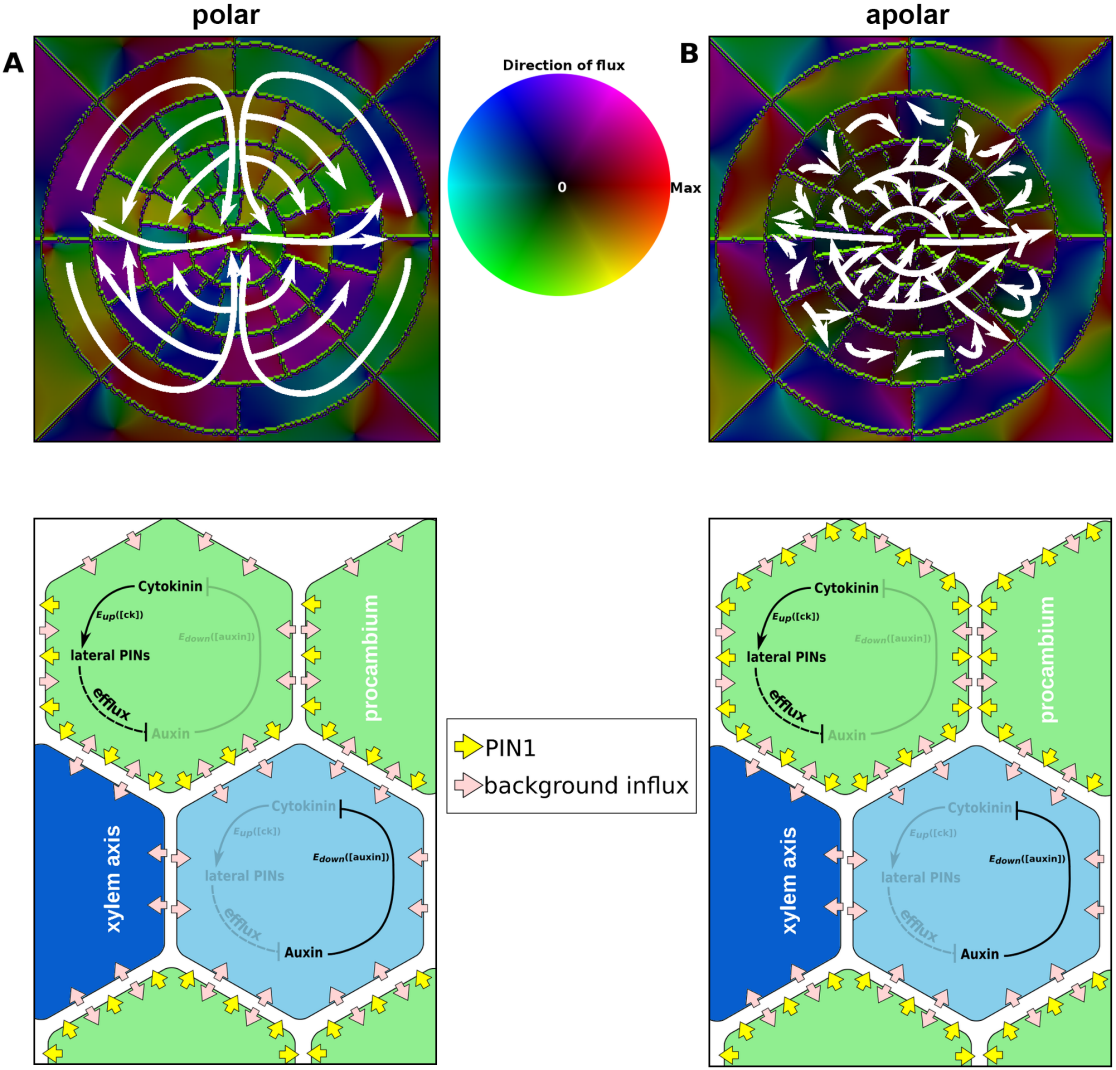
An auxin circuit is generated in the stele by polar localisation of PIN1. (A, B) A heatmap of the auxin flux in the stele of plants with polar (A) and apolar (B) PIN1 localisation with arrows depicting the overall flux pattern. The schematic models in the lower panels only show PIN1 localisation, since PIN3, PIN7, and the auxin importer are apolarly localised, as in all other simulations.

Polar localisation of PIN1 towards the xylem axis results in increased co-ordination of the fluxes through the procambium and pericycle (Fig 10A and 10B), which can be seen by a detailed examination of the flux patterns (S10 Fig). In simulations with apolar PIN1 localisation, the radial flux alternated between inwards and outwards along the circumference of the pericycle; by contrast, roots with polar localisation of PIN1 presented consistent inwards radial flux in the pericycle (despite spikes of outwards flux) except at the protoxylem poles (Fig 10A, S10C Fig). Likewise, the direction of the angular flux within the pericycle presented strong variations in the simulations with apolarly localised PIN1, but was consistently directed towards the xylem axis in the simulations with polar PIN1 (Fig 10A, S10D Fig). In addition, in polar simulations angular flux was consistently towards the phloem poles in the endodermis (Fig 10A, S10F Fig), but this was not the case in apolar simulations (S10F Fig). In the procambium, radial flux is inwards in simulations of roots with polar PIN1 (again, the protoxylem position is an exception), despite spikes of outwards flux in the cell walls, while roots with apolar PIN1 have a domain of outward flux in the procambium spanning over a quarter of the circumference (Fig 10A and 10B, S10A Fig). Unlike in the pericycle, the angular flux in the procambium does not show strong variations; however, simulations with polar PIN1 show four quadrants of angular flux, with each protoxylem pole receiving auxin via flux from two of the quadrants (Fig 10A, S10B Fig). By contrast, simulations of roots with apolar PIN1 localisation only have two domains of angular flux, both oriented towards the same protoxylem pole (Fig 10B, S10B Fig).

The co-ordinated fluxes resulting from polar subcellular localisation of PIN1 generate an auxin circuit. Auxin flows towards the phloem poles and then inwards; in the procambium, it flows towards the xylem axis and then out through the protoxylem poles (Fig 10A). This circuit is absent in simulations with apolar localisation of PIN1 in the procambium (Fig 10B), which show outward flux at the protoxylem poles but disordered flux in the pericycle and procambium. The auxin circuit leads to higher levels of auxin accumulation within the axis (Fig 9D), despite having greater levels of flux within the stele. Despite these significant differences, both polar and apolar PIN1 localisations generate an auxin maximum in the xylem axis, prompting us to consider whether the auxin circuit may confer other advantages, such as increased robustness.

### The auxin circuit confers robustness and flexibility

**Fig 11:**
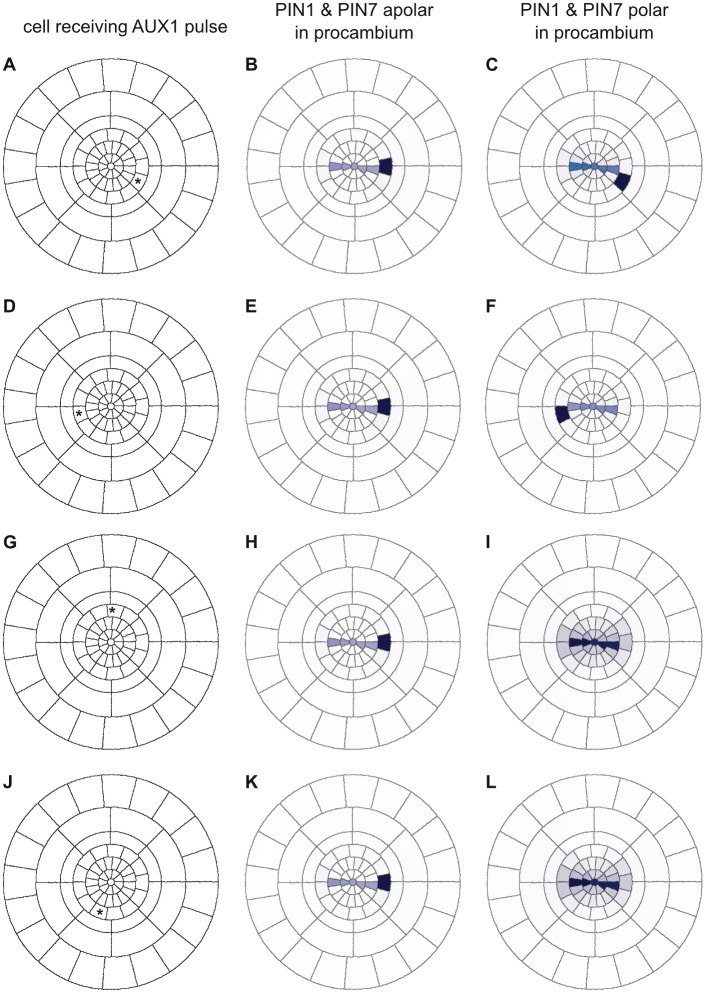
Polar localisation of PIN1 favours AUX1 activation in the xylem-pole pericycle cells. A 120-second pulse of AUX1 activation was provided to the pericycle cell marked with an asterisk (A, D, G, J). In simulations with apolar PIN1 and PIN7, this resulted in continued AUX1 activity and auxin accumulation in a specific pericycle cell, regardless of which cell received the pulse (B, E, H, K). By contrast, the pulse only led to persistent activation in xylem-pole pericycle cells in simulations with polar PIN1 and PIN7 (C, F, I, L). Auxin levels are depicted using a ‘DR5-like’ scale.

In order to explore whether the auxin flux circuit generated by polar PIN1 localisation confers additional robustness or other benefits to the developing root, we investigated the resilience of the auxin maximum to transient *AUX1* activation (and thus auxin accumulation) in pericycle cells in simulations with either apolar or polar localisation of PIN1 and PIN7. Upregulation of *AUX1* in xylem-pole pericycle cells is one of the earliest steps in lateral root initiation [39]. We replicated this in simulations by activating *AUX1* in a focal pericycle cell for a period of 120 seconds in a transverse root section located shootwards of previous simulations, where lateral root primordia are primed [58] (Fig 11A, 11D, 11G and 11J). Since the PIN3 expression pattern changes quickly shootwards of the QC [3] and its regulation is not as well understood, we only included *PIN1* and *PIN7* in these simulations; furthermore, we introduced auxin-regulated *AUX1* expression, but limited to only the pericycle cells. We also evaluated the effect of PIN polarity in the pericycle independently of the procambium.

Simulations in which PIN1 and PIN7 were apolarly localised in the pericycle consistently retained auxin accumulation in the xylem axis when an *AUX1* pulse was provided to a pericycle cell, regardless of the polarity of the PINs in the procambium cells (S11 Fig). However, this was not the case when PIN1 and PIN7 were polar in the pericycle. When polar localisation of PIN1 and PIN7 in the pericycle was combined with apolar localisation in the procambium, a pulse of *AUX1* activation was sufficient to stabilise *AUX1* upregulation and auxin accumulation in a xylem-pole pericycle cell. In geometric cross sections, activation within any pericycle cell could trigger the response, while in realistic root layouts it was limited to a subset of xylem-pole pericycle cells. However, auxin always accumulated in the same pericycle cell in geometric layouts, regardless of which cell received the *AUX1* pulse (Fig 11B, 11E, 11H and 11K). In realistic layouts, a subset of the xylem-pole pericycle cells accumulated auxin following an *AUX1* pulse (S12E and S12H Fig), while the other xylem-pole pericycle cells could not compete with the xylem axis for auxin accumulation (S12B and S12K Fig). By contrast, introducing polar PIN1 and PIN7 localisation in the procambium resulted in two important differences: (i) any xylem-pole pericycle cell (but no other pericycle cell) can present auxin accumulation and stable *AUX1* expression; and (ii) this is only triggered specifically in the cell that received the *AUX1* pulse (Fig 11C, 11F, 11I and 11L). The same result was found for both geometric and realistic root layouts (S12 Fig). Thus, the coordinated flux pattern generated by polar PIN1 localisation provides a mechanism for auxin to accumulate specifically in the xylem-pole pericycle cells, whenever another mechanism is able to trigger an initial (transient) increase in *AUX1* expression in those cells. Possible mechanisms that could trigger such an initial increase have been discussed elsewhere (see [59] for a review).

**Fig 12:**
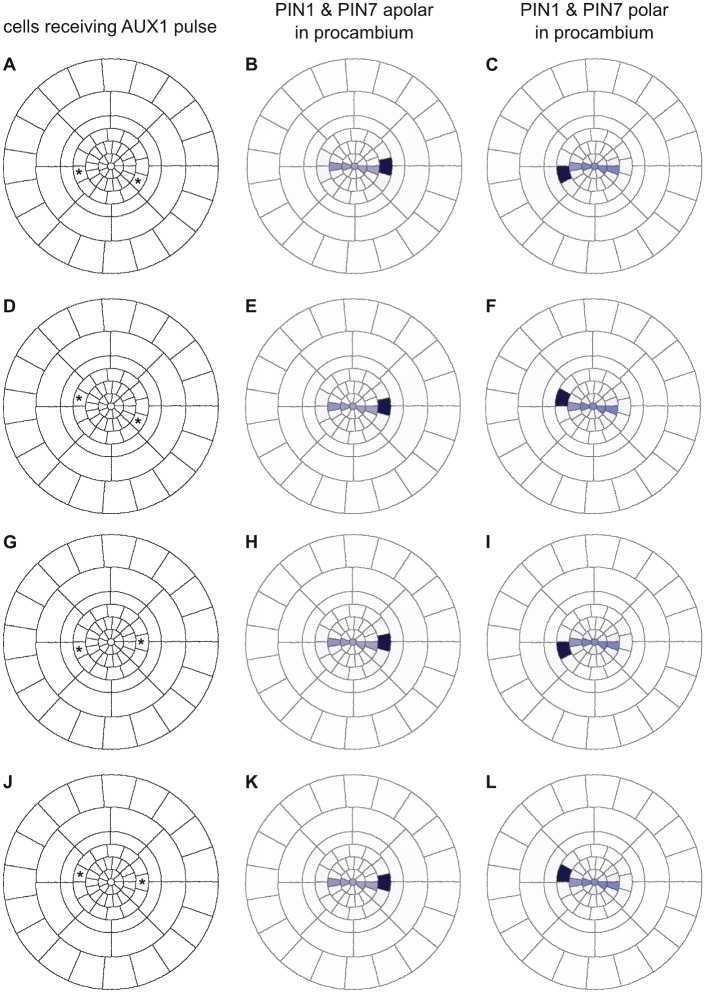
Xylem-pole pericycle cells compete for auxin accumulation in simulations of geometric cross sections. A simultaneous 120-second pulse of AUX1 activation in two pericycle cells marked with asterisks (A, D, G, J) results in auxin accumulation in only one cell. In simulations with apolar PIN1 and PIN7, the same cell accumulates auxin (B, E, H, K), whereas the identity of the winning cell varies in simulations with polar PIN1 and PIN7 (C, F, I, L).

**Fig 13:**
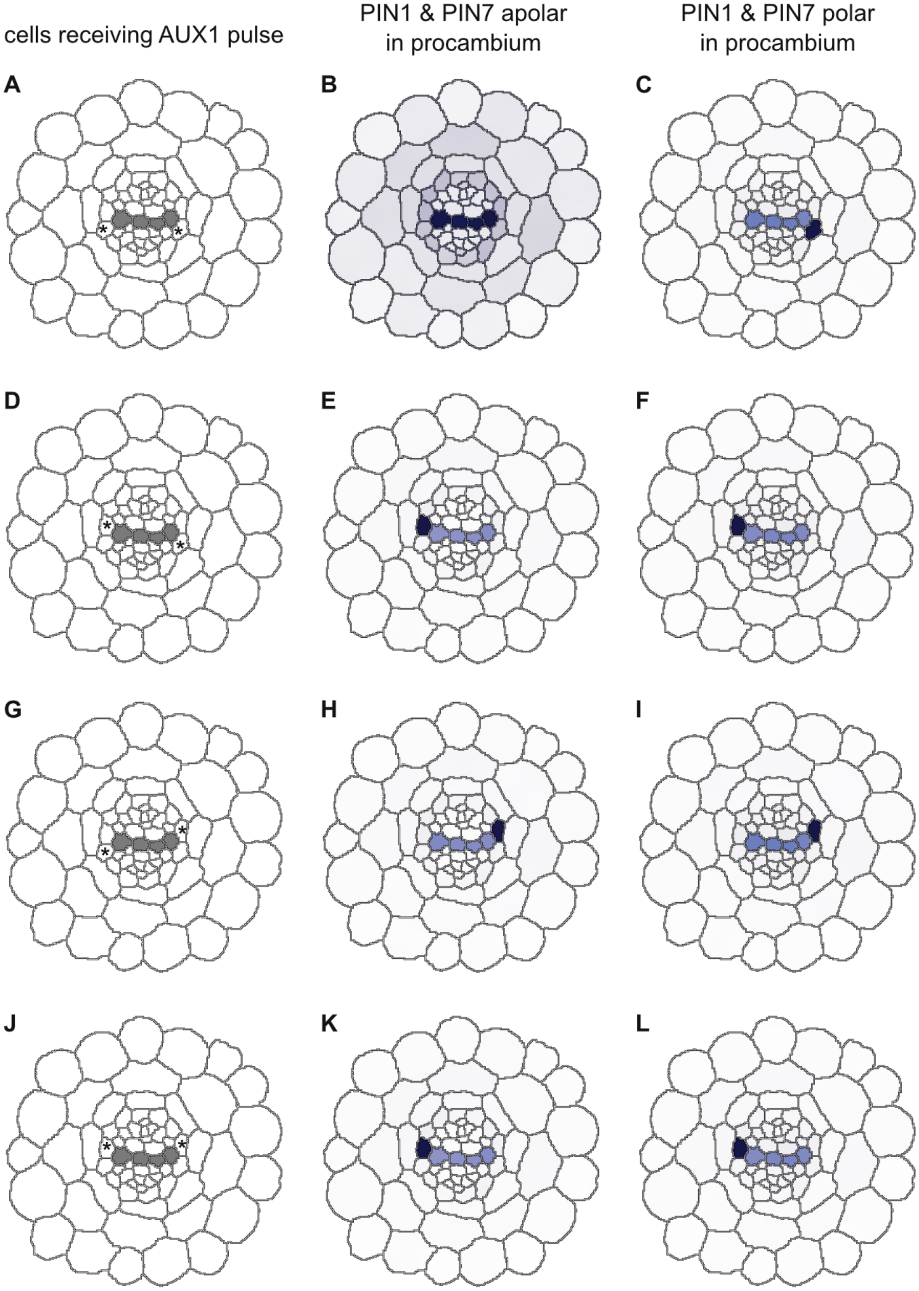
Xylem-pole pericycle cells compete for *AUX1* activation in simulations of realistic cross sections. A simultaneous 120-second pulse of *AUX1* activation in two pericycle cells marked with asterisks (A, D, G, J) results in auxin accumulation in only one cell. In simulations with apolar PIN1 and PIN7, the same cell accumulates auxin (B, E, H, K), whereas the identity of the winning cell varies in simulations with polar PIN1 and PIN7 (C, F, I, L). Auxin levels are depicted using a ‘DR5-like’ scale.

In *Arabidopsis*, lateral roots are initiated by the division of xylem-pole pericycle cells [1]; the initiation is triggered by auxin accumulation in the pericycle cells [60] and tends to alternate between opposite xylem poles [61]. The alternating left-right pattern of lateral root positioning is dependent on *AUX1*[58]. To evaluate whether this behaviour adheres in our computational model, we conducted simulations of roots with polarly localised PIN1 and PIN7 in which *AUX1* was simultaneously activated in opposite pericycle cells for 120 seconds in both geometric (Fig 12A, 12D, 12G and 12J) and realistic (Fig 13A, 13D, 13G and 13J) cross sections. In no case did *AUX1* remain active in both pericycle cells following a pulse; in simulations with polar PINs, one of the two focal cells invariably retained *AUX1* and thus accumulated auxin, while the other subsided (Fig 12C, 12F, 12I and 12L; Fig 13C, 13F, 13I and 13L). When PIN1 and PIN7 were apolarly localised in the procambium, simultaneous *AUX1* pulses resulted in an auxin pattern similar to a single pulse; geometric cross sections consistently accumulated auxin in the same cell, while realistic cross sections only accumulated auxin in a subset of the xylem-pole pericycle cells (Fig 12B, 12E, 12H and 12K; Fig 13B, 13E, 13H and 13K).

To further illustrate the potential of a “triggered” xylem-pole pericycle cell to suppress other xylem-pole pericycle cells to get triggered, we introduced a delay between the application of the AUX1 pulse in the two cells. For a sufficiently long delay, the cell which received the pulse earlier consistently accumulated auxin at the expense of the other cell. For realistic cross sections, a delay of 5s was sufficient (S13 Fig); for the geometric cross section, a 100s delay was sufficient, while a 20s delay was too brief (S14 Fig). This indicates a bistability induced by the transverse auxin flows; the system ensures that either one or the other pole can accumulate auxin. A situation in which both poles accumulate auxin is unstable, with tiny initial biases related to AUX1 and auxin levels and tissue layout determining which cell will eventually present persistent accumulation.

## Discussion

To date, efforts to understand auxin transport have focused primarily on the longitudinal dimension [31, 62–65]; recently, however, experiments have indicated a role for transverse auxin transport in vascular patterning [3], and computational research has begun to address this question [4, 41]. In this work we have demonstrated that the experimentally derived model is sufficient to maintain an auxin maximum along the xylem axis. We have also identified a novel role for active auxin influx in this network, and discovered a connection between subcellular polarity and the emergence of robustness and flexibility at the level of the whole tissue. These insights into the dynamics of transverse auxin transport will play an important role in future attempts to construct three-dimensional models of auxin transport and root growth in *Arabidopsis*.

Our simulations indicate that the mutually inhibitory auxin-cytokinin loop [3] is capable of maintaining an auxin maximum in the xylem axis. In reality, however, the auxin maximum becomes gradually confined to the protoxylem, while our simulations consistently show sustained auxin accumulation throughout the xylem axis, akin to the pattern observed near the quiescent centre in *Arabidopsis* roots [3]. Since the regulatory dynamics captured in our model do not recapitulate this change in the auxin pattern, we suggest that other factors are required to drive it; for example, *PIN3* expression is known to expand into the metaxylem cells in the same domain where auxin signalling disappears from them [3] and is a plausible candidate.

In addition, regulation of the expression level of PIN transporters by this network is required in order to produce the auxin pattern observed in several conditions, indicating that not only the localisation of the PINs but also the correct regulation of the strength of their expression plays an important role in auxin patterning. Our analysis of the auxin fluxes in simulations of *wol*-like roots with and without dynamic regulation of the PINs demonstrates that, despite identical localisation of the PINs, dynamic changes in their expression level lead to local differences in the auxin fluxes, causing differences in auxin distribution and flux at the tissue scale. In addition to its relevance to future computational models of auxin transport, this insight has implications for experimental biologists. If differences in expression level alone (i.e., without changes in PIN localisation) can lead to different patterns, this highlights the need to record not only the localisation of the PINs in any particular context, but also how strongly they are expressed and how this is regulated, which may involve changes in PIN expression or abundance (e.g., [66]).

While the addition of PIN regulation reconstitutes the observed auxin distribution in *wol*, dynamic simulations of cytokinin-treated roots do not show a good match with the experimental data, though they correspond more closely than static simulations. The simulations show unexpectedly high levels of auxin in the procambium, phloem, and pericycle cells. Several possible explanations for this discrepancy exist. Our simulations conflate *AUX1*, *LAX1*, and *LAX2* into a single importer, ignoring differences in their expression pattern and auxin uptake ability. In fact, LAX2, the importer present throughout the stele (Fig 3K), takes up auxin at a lower rate than AUX1 and LAX1 [48], which have a more specific expression pattern (Fig 3I and 3J). As a result, our simulations likely overestimate the amount of auxin taken up by these tissues. Furthermore, while the simulations include cytokinin-induced changes in the expression of the PIN transporters (S4D–S4F Fig), the possible effect of cytokinin on the importers is not included. Cytokinin-induced regulation of the AUX1/LAX genes or changes in their expression pattern may be able to explain the persistent mismatch. Indeed, it has recently been shown that expression of *AUX1* and *LAX2* is reduced by cytokinin treatment [67].

Our simulations indicate that active auxin import plays an unexpected key role in accumulating a sufficient level of auxin in the xylem axis to result in protoxylem specification. We confirmed this prediction by observing a high proportion of roots with defective protoxylem in the triple auxin importer mutant, *aux1lax1lax2*; furthermore, protoxylem formation was found to be hypersensitive to cytokinin in the *aux1* mutants. These findings are consistent with earlier studies, in which aberrant phyllotaxis and defective secondary xylem differentiation were found in double, triple, and quadruple importer mutants, but not in the single mutants [44, 49], although more recent work has also reported discontinuities in the vascular strands of the leaves of *lax2* mutants [48], suggesting that subtle phenotypes may be found in single importer mutants as well. In our analysis of the root tip, we unexpectedly found that protoxylem formation is resistant to cytokinin treatment in the *lax2* mutant. This may be due to the downregulation of *LAX2* by cytokinin [67]; lower *LAX2* expression in procambium cells adjacent to the xylem axis may reduce their ability to compete with the xylem axis for auxin, allowing auxin to accumulate in the axis despite other changes induced by cytokinin. However, contrary phenotypes are observed in the root and leaf, suggesting that the picture may be more complex. It is also interesting to note that *AUX1* and *LAX3*, but not *LAX1* or *LAX2*, have been reported to play a role in lateral root initiation [48], while *AUX1* and *LAX1* or *LAX2* (but not *LAX3*) have been reported to regulate phyllotaxis [44]. Here, we have identified a role for *AUX1* and *LAX1* or *LAX2* (but not *LAX3*) in vascular specification in the primary root, suggesting the possibility that specific *LAX* genes may act in concert with *AUX1* in different developmental contexts, amplifying and buffering the auxin pattern generated by the PIN transporters.

We found that an even distribution of cytokinin throughout the root is sufficient to activate the PINs in a way that results in efficient auxin transport to the xylem axis and patterning; a gradient centred around local sites of cytokinin biosynthesis, whether in the xylem or the phloem, is not required and is unlikely. Our results demonstrate the difficulty in generating a cytokinin gradient on the cross-sectional scale of the *Arabidopsis* root via an effectively diffusion-driven process. Forming a reliably informative gradient within the stele requires a membrane permeability of 0.01*μ*m/*s* and an apoplastic diffusion coefficient of 0.6*μ*m^2^/*s*. Altering these parameters results in either a steepening of the gradient to form a step-like cytokinin pattern (in which the cytokinin is effectively trapped in the source tissues) or a flat distribution of cytokinin throughout the root (when cytokinin can escape from the source cells).

To generate a graded distribution spanning the stele, it was necessary to significantly reduce the diffusion coefficient of cytokinin within the apoplast. In retrospect, the reason for this is clear. The source cells can retain high cytokinin levels when membrane permeability is low, but rapid cytokinin diffusion within the apoplast allows cytokinin to readily move to all of the remaining cells in the stele, which will take it up based on the membrane permeability. While lowering membrane permeability can trigger a difference in cytokinin levels, it is still the diffusion coefficient which determines whether the difference takes the form of a steep drop-off or an informative smooth gradient. In addition to demonstrating the implausibility of an informative cytokinin gradient forming on this scale via diffusive processes (whether or not combined with apolar transport), this also highlights the importance of explicitly including the apoplast in computational models of plants so as to avoid such artefacts.

**Fig 14:**
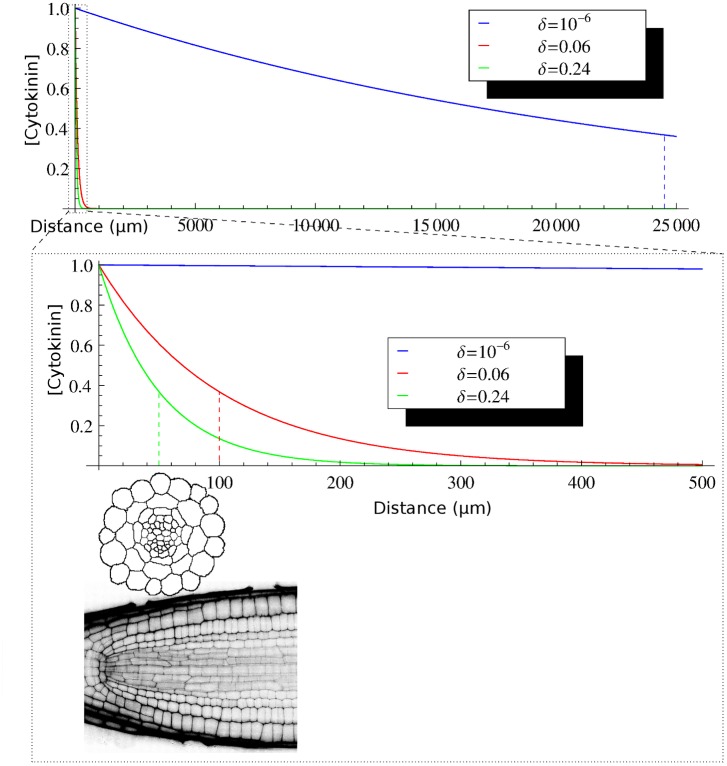
The spatial scale of the cytokinin gradients. The spatial steady state profile of cytokinin in a source-diffusion-decay model, for different degradation rates. The lower panel is zoomed in (50x) to the dotted area within the upper panel; the root sections are drawn to scale for comparison with the gradient. Dotted lines mark the characteristic length of each gradient. The blue line shows the cytokinin distribution with degradation rate and diffusion coefficient similar to auxin; the red and green lines represent gradients that could be informative at the transverse scale of the root meristematic zone.

This finding is reinforced by spatial-temporal considerations of hormone diffusion within the context of this system: in order to be informative on the transverse scale of the *Arabidopsis* stele (around 50*μ*m), a gradient would need to have a characteristic length of roughly the same order (Fig 14). Generating such a gradient via diffusion would require the degradation rate or diffusion coefficient of cytokinin to differ by five orders of magnitude from those used for auxin here (see Materials and Methods) and elsewhere [31, 39]; such values have in fact been used to produce cytokinin gradients in other computational models [38]. However, such a large difference seems unlikely, given that auxin and cytokinin are molecules of approximately the same size, composition and shape. Furthermore, a consequence of kinetic parameter values that would give rise to a potentially informative transverse gradients would be that the root mean square displacement of cytokinin (the distance which molecules are typically able to move before breakdown) would be limited to approximately 75*μ*m, which is inconsistent with the observation that cytokinin travels over long distances *in planta*[52].

In addition, the timescale on which cytokinin patterning takes place would also be four orders of magnitude slower than for auxin. We have shown that application of cytokinin at the surface of the root leads to activation of a cytokinin response marker within the stele, at a distance of approximately 50*μ*m, within hours of treatment. We cannot rule out the possibility of a systemic response to exogenous cytokinin application; however, such a mechanism would seem to preclude the possibility of deriving positional information from cytokinin. While our data are not sufficient to provide a quantitative estimate of the cytokinin movement rate, they do establish a lower limit on the order of tens of microns per hour. This is on the scale of the upper limit of the parameters required to form an informative gradient in our simulations. Spatial patterning of cytokinin on a scale appropriate to affect vascular patterning in the root therefore seems unlikely, despite evident patterns in cytokinin signalling and response within these tissues. While cytokinin remains important in such a scenario, the patterning process is ultimately driven by auxin.

We therefore suggest that cytokinin is unlikely to form informative gradients in contexts at the scale of transverse vascular patterning in developing *Arabidopsis* roots. We propose that the observed spatial patterns in cytokinin signalling (Fig 1C) may instead result from patterning of the cytokinin response machinery, perhaps due to modifications by auxin signalling. Alternatively, these patterns may arise from local, directed cytokinin transport, potentially combined with localised production and/or degradation, a possibility which we cannot exclude. Regardless of the preferred scenario, our analysis raises questions and challenges regarding the mechanics and dynamics of cytokinin transport which must be further addressed by experiments and more modelling alike. Furthermore, given that the parameters used here and elsewhere [38] to generate a locally informative cytokinin gradient are incompatible with long-distance movement of cytokinin, our findings highlight how local patterning mechanisms may have implications at the scale of the whole organism. It is therefore important when constructing verbal or conceptual models to consider constraints which are introduced by conflicting requirements at different spatial scales. Quantitative considerations can lead to different qualitative mechanisms for how patterning occurs. Investigating the plausibility of a cytokinin gradient in quantitative terms has led us to a qualitatively different way of thinking about the role of cytokinin, where the hormone is spatially homogeneous and patterning emerges from the response machinery.

This contrasts with the perspective reflected in a recent computational study on the same network [41], which argued that local cytokinin biosynthesis is crucial for vascular patterning. Although the model in [41] distinguishes between cytokinin levels and response, the cytokinin response only serves to regulate cell division; the auxin/PIN dynamics are driven by cytokinin levels, not cytokinin response. A gradient in cytokinin itself is thus crucial for the operation of their model; indeed, they establish a cytokinin gradient by using a membrane permeability equivalent to the lowest value we used in Fig 7. However, their results rely on the biologically implausible requirement of extremely little to no cytokinin diffusion within the apoplast. Our findings therefore fundamentally contrast with those reported in [41], in which severely restricted cytokinin movement establishes a gradient in cytokinin levels which drives PIN localisation and thus auxin dynamics; the auxin pattern then modulates the cytokinin response to control cell division. Thus, a gradient in cytokinin levels is a driving element in their model. By contrast, while we concur that local biosynthesis may be important to ensure that enough cytokinin is available to the developing root, we argue that a cytokinin gradient is not required for the patterning, and it is, in fact, challenging to establish such a pattern on the cross-sectional scale of the *Arabidopsis* root. Although directed cytokinin transport would allow such a pattern to be established straightforwardly, there is currently no indication of such a process.

We note that similar constraints apply in the embryo, where spatial scales even smaller than in the root make the presence of an informative cytokinin gradient even less likely. Although the embryo is too small for the formation of an informative cytokinin gradient solely via diffusion, a cytokinin signalling pattern has been proposed as a source of spatial information in this context [41], raising the question of how this signalling pattern is established. A cytokinin gradient has also been proposed to play a role in patterning processes in other plant organs (e.g., [38]), and these proposals will likewise have to be re-evaluated in light of our findings. While cytokinin transport or patterned cytokinin perception may address these difficulties, this only pushes the patterning question back a level: how, then, are the patterns in perception or transport established?

Another outstanding question is how low cytokinin signalling levels are maintained in the metaxylem position. In the current work, auxin accumulates throughout the xylem axis, including the metaxylem, and inhibits cytokinin signalling. However, this inhibition is mediated via AHP6, which is expressed in the protoxylem but not in the metaxylem [35]. Although auxin signalling is seen throughout the xylem axis [3], consistent with our model, this alone is insufficient to account for the low levels of cytokinin signalling in these cells. The same assumption was used in a model of the auxin-cytokinin loop in the embryonic root [41], where auxin was considered to inhibit the cytokinin response in all cells, even those without *AHP6* expression, such as the metaxylem. Implicit in these approaches is the assumption that an auxin-responsive gene acts in the place of AHP6 in the metaxylem to inhibit cytokinin signalling. This assumption was made explicit in another modelling effort, which introduced an additional, hypothetical negative regulator of cytokinin, suggested to be a CKX or type A ARR, to simulate a cytokinin response pattern which matched experimental observations [4]. However, in this case the putative cytokinin repressor was not regulated by auxin, but was directly positioned in the metaxylem cells. By explicitly including the activity and regulation of *AHP6* and its unknown partner, future modelling efforts could test the effect of different regulatory mechanisms (e.g, differences in auxin sensitivity between *AHP6* and its redundant partner), hormone treatments or mutant combinations; the predictions from these simulations would help guide experimental biologists to identify the missing components in this network.

In our simulations, the auxin distribution pattern within the maximum in the xylem axis results from an auxin circuit generated by the polar subcellular localisation of PIN1 and PIN7 in the procambium and pericycle. To date, the challenges in detecting PIN polarity in cross-sections have impaired a definitive experimental determination of the subcellular localisation of the PINs in this context [3]. Our simulations have shown that polar efflux within the stele organises the auxin flux into a circuit directed towards the xylem axis. In addition to providing robustness to the auxin maximum along the xylem axis, this auxin circuit provides a mechanism favouring the xylem-pole pericycle cells, the very cells which will later initiate lateral root primordia. In the absence of the auxin circuit (i.e., if PIN1 and PIN7 are apolarly localised), geometric factors determine which pericycle cells (if any) are sensitive to increased auxin import leading them to accumulate auxin. For example, in our simulations of a geometric cross section with apolar PIN1 and PIN7, the auxin flux in the procambium is oriented towards a single protoxylem pole (Fig 10B) because of the geometry of the cross section, which contains a series of consecutive cell walls that are perfectly aligned from the central stele cell up to this cell. This bias is not seen in simulations with polar PIN localisation, in which the auxin circuit stabilises the xylem axis against such perturbations; increased auxin import persists in the xylem-pole pericycle cells but is transient in other pericycle cells. Thus, the auxin circuit not only buffers the xylem axis against fluctuations in auxin import in other cells, but also provides the flexibility needed for auxin to accumulate in the xylem-pole pericycle, an event which plays a critical role in lateral root initiation [39, 60]. Furthermore, our results have shown that pericycle cells on opposite xylem poles compete for auxin accumulation, consistent with the observation that lateral roots are initiated in an alternating left-right pattern, never appearing concurrently at both sides [61]. Nevertheless, we cannot describe the factors which lead to one pole being favoured over the other in any given instance, although we have shown that the relative timing of activation is important. A full understanding of this phenomenon is likely to require incorporating existing longitudinal and transverse auxin transport models into a three-dimensional model of the root.

In addition to their specific implications for development in *Arabidopsis thaliana*, our simulations have also uncovered a novel mechanism by which processes can be made sensitive to the geometry of a tissue. Such a sensitivity, which may be desirable in particular contexts, is dependent on the polar subcellular localisation of a transporter and can be modulated by the effect of other transporters and network components. In this context, we have shown how such a mechanism can alter the transport dynamics to generate a coordinated flux pattern at the scale of a tissue or organ. This circuit thus serves to link patterning at several levels, connecting subcellular polarity to tissue-level organisation.

## Supporting Information

S1 Fig Auxin regulation of the auxin importers. A qRT-PCR of the auxin importers following treatments with 1 *μ*m IAA shows modest increase in expression in response to auxin. Error bars depict standard deviation; statistically significant differences in gene expression (*p* < 0.05) in IAA-treated roots in comparison to the respective mock are marked with asterisks.
(TIF)

S2 Fig Auxin regulation of the importer does not significantly alter the auxin pattern. The ‘DR5-like’ output from simulations with (A) hormonal regulation of the PINs and auxin-regulation of the importer in (B) wild type, (C) *wol*, and (D) cytokinin-treated geometric root sections.
(TIF)

S3 Fig Transporter regulation is needed to generate the correct auxin pattern. The ‘DR5-like’ output from simulations of a realistic root with unregulated PIN expression (A–C) do not in all cases generate the observed auxin signalling pattern (D–F). (G–I) Simulations in which the PINs are regulated correspond more closely to the experimental data. (A, D, G) Wild-type roots; (B, E, H) *wol*; (C, F, I) cytokinin-treated roots. Insets show the *wol* roots at the same scale as others; the larger *wol* figures are scaled 250% to improve visibility.
(TIF)

S4 Fig Localisation of the PIN transporters in the simulations. (A–C) Localisation of (A) PIN1; (B) PIN3; and (C) PIN7, according to experimental observations in *wol*. (D–F) Localisation of (D) PIN1; (E) PIN3; and (F) PIN7, according to experimental observations in wild-type roots treated with cytokinin. Localisations are based on observations reported in [3]; lighter colour indicates a lower maximum expression level.
(TIF)

S5 Fig Auxin fluxes within the stele in dynamic *wol* simulations. (A, C) Graphs depicting the radial (A) and angular (C) fluxes in the procambium of static (black) and dynamic (red) simulations of *wol*. The fluxes are plotted as a function of the angular position along the circumference of the root from 0°–360°. (B, D) Graphs depicting the radial (B) and angular (D) fluxes in the pericycle of static (black) and dynamic (red) simulations of *wol*. Fluxes are again plotted as a function of the angular position.
(TIF)

S6 Fig Mutation of auxin transporters alters the auxin pattern. The simulated DR5 pattern in geometric (A–C) and realistic (D–F) roots of (A, D) *pin1*, (B, E) *pin7*, and (C, F) auxin importer mutants.
(TIF)

S7 Fig A steep cytokinin gradient does not alter the final auxin distribution. (A) Heatmap of cytokinin distribution in a steep gradient centred around the phloem poles. ‘DR5-like’ output from simulations of (B) wild-type and (C) *wol* roots produce the same pattern as with a flat cytokinin distribution. (D) Heatmap of a cytokinin gradient formed by synthesis in the xylem axis. (E) Simulation of a wild-type root produces the same auxin pattern as with a flat cytokinin distribution.
(TIF)

S8 Fig Severely reduced cytokinin movement is required to establish a cytokinin gradient across the *Arabidopsis* root via diffusion from a local biosynthesis source in the phloem poles. To establish a cytokinin gradient without altering the diffusion coefficient of cytokinin, we introduced bidirectional membrane permeability. Decreasing the membrane permeability (moving right within rows) concentrates cytokinin in the source cells (here, the phloem poles), but a gradient is not established because the diffusion within the apoplast evens out the distribution within the rest of the root. Decreasing the diffusion coefficient (downwards in columns) in the apoplast transforms the step-wise cytokinin distribution pattern into a true gradient. Parameter values are given in Table 6.
(TIF)

S9 Fig Auxin-driven cytokinin biosynthesis also requires severely reduced cytokinin movement to establish a cytokinin gradient across the *Arabidopsis* root. All cells in the cross section can synthesize cytokinin in an auxin-dependent manner. As in the simulations with fixed cytokinin biosynthesis (Fig 7, S8 Fig), decreasing the membrane permeability (moving right within rows) does not establish a gradient because the diffusion within the apoplast evens out the distribution within the rest of the root. Decreasing the diffusion coefficient within the apoplast (moving downwards within the columns) transforms the step-wise cytokinin distribution pattern into a true gradient. Parameter values are given in Table 6 and Table 4.
(TIF)

S10 Fig Auxin fluxes in simulations with asymmetric or apolar subcellular localisation of PIN1. Graphs depicting radial (A, C, E) and angular (B, D, F) auxin fluxes in simulations with apolar (red) and polar (red) *PIN1* localisation. The fluxes are plotted as a function of the angular position along the circumference of the root from 0° − 360°. (A, B) Radial (A) and angular (B) fluxes in the procambium. (C, D) Radial (C) and angular (D) fluxes in the pericycle. (E, F) Radial (E) and angular (F) fluxes in the endodermis.
(TIF)

S11 Fig Pericycle cells do not accumulate auxin when PIN1 and PIN7 are apolar in the pericycle. ‘DR5-like’ output of simulations in which PIN1 and PIN7 are apolar in the pericycle and a focal cell receives an AUX1 pulse. (A) The cell receiving the AUX1 pulse. The resulting auxin pattern when PIN1 and PIN7 are (B) polar and (C) apolar in the procambium.
(TIF)

S12 Fig Polar PIN localisation favours *AUX1* accumulation in the xylem-pole pericycle. A 120-second pulse of *AUX1* activation was provided to the pericycle cell marked with an asterisk (A, D, G, J). In simulations with apolar PIN1 and PIN7, only two of the xylem-pole pericycle cells could maintain *AUX1* activity and accumulate auxin (B, E, H, K). By contrast, the pulse caused persistent activation in any xylem-pole pericycle cell in simulations with polar PIN1 and PIN7 (C, F, I, L). Auxin levels are depicted using a ‘DR5-like’ scale.
(TIF)

S13 Fig A short delay is sufficient to determine which cell xylem-pole pericycle cell accumulates auxin in simulations of realistic root sections. A 120-second pulse of *AUX1* activation was provided to two pericycle cells with a five second delay between the cells marked 1 and 2 (A, C, E). The first cell to receive the *AUX1* pulse retains *AUX1* activation and accumulates auxin (B, D, F). Auxin levels are depicted using a ‘DR5-like’ scale.
(TIF)

S14 Fig Timing can determine which cell xylem-pole pericycle cell accumulates auxin in simulations of geometric cross sections. A 120-second pulse of *AUX1* activation was provided to two pericycle cells with a 20 s or 100 s delay between the cells marked 1 and 2 (A, D, G, J). Following a 20 s delay, the identity of the cell accumulating auxin is the same as when the pulse is simultaneous (B, E, H, K), but with a longer delay of 100 s the cell receiving the earlier pulse accumulates auxin (C, F, I, L). Auxin levels are depicted using a ‘DR5-like’ scale.
(TIF)

## References

[1] Dolan L, Janmaat K, Willemsen V, Linstead P, Poethig SR, Roberts K, et al Cellular organisation of the Arabidopsis thaliana root. Development. 1993;119:71–84. http://dev.biologists.org/content/119/1/71 8275865 10.1242/dev.119.1.71

[2] Mähönen AP, Bonke M, Kauppinen L, Riikonen M, Benfey PN, Helariutta Y. A novel two-component hybrid molecule regulates vascular morphogenesis of the Arabidopsis root. Genes & Development. 2000 12;14(23):2938–2943. Available from: http://www.genesdev.org/cgi/doi/10.1101/gad.189200. 10.1101/gad.189200 11114883 PMC317089

[3] Bishopp A, Help H, El-Showk S, Weijers D, Scheres B, Friml J, et al A mutually inhibitory interaction between auxin and cytokinin specifies vascular pattern in roots. Current Biology. 2011 6;21(11):917–26. Available from: http://www.sciencedirect.com/science/article/pii/S0960982211004337. 10.1016/j.cub.2011.04.017 21620702

[4] Muraro D, Mellor N, Pound MP, Help H, Lucas M, Chopard J, et al Integration of hormonal signaling networks and mobile microRNAs is required for vascular patterning in Arabidopsis roots. Proceedings of the National Academy of Sciences. 2014 1;111(2):857–862. Available from: http://www.pnas.org/cgi/doi/10.1073/pnas.1221766111. 10.1073/pnas.1221766111 PMC3896157 24381155

[5] Cruz-Ramírez A, Díaz-Triviño S, Blilou I, Grieneisen VA, Sozzani R, Zamioudis C, et al A bistable circuit involving SCARECROW-RETINOBLASTOMA integrates cues to inform asymmetric stem cell division. Cell. 2012 8;150(5):1002–1015. Available from: http://linkinghub.elsevier.com/retrieve/pii/S009286741200880X. 10.1016/j.cell.2012.07.017 22921914 PMC3500399

[6] Coenen C, Lomax TL. Auxin-cytokinin interactions in higher plants: old problems and new tools. Trends in Plant Science. 1997;2(9):351–356. Available from: http://www.sciencedirect.com/science/article/pii/S1360138597846237. 10.1016/S1360-1385(97)84623-7 11540614

[7] Vanstraelen M, Benková E. Hormonal interactions in the regulation of plant development. Annual Review of Cell and Developmental Biology. 2012;28:463–87. Available from: http://www.ncbi.nlm.nih.gov/pubmed/22856461. 10.1146/annurev-cellbio-101011-155741 22856461

[8] El-Showk S, Ruonala R, Helariutta Y. Crossing paths: cytokinin signalling and crosstalk. Development. 2013 3;140(7):1373–1383. Available from: http://dev.biologists.org/cgi/doi/10.1242/dev.086371. 10.1242/dev.086371 23482484

[9] Zhao Z, Andersen SU, Ljung K, Dolezal K, Miotk A, Schultheiss SJ, et al Hormonal control of the shoot stem-cell niche. Nature. 2010 6;465(7301):1089–1092. Available from: 10.1038/nature09126 20577215

[10] Dello Ioio R, Nakamura K, Moubayidin L, Perilli S, Taniguchi M, Morita MT, et al A genetic framework for the control of cell division and differentiation in the root meristem. Science. 2008 11;322(5906):1380–4. Available from: http://www.sciencemag.org/content/322/5906/1380.abstract. 10.1126/science.1164147 19039136

[11] Bainbridge K, Sorefan K, Ward S, Leyser O. Hormonally controlled expression of the Arabidopsis MAX4 shoot branching regulatory gene. The Plant Journal. 2005;44(4):569–580. Available from: 10.1111/j.1365-313X.2005.02548.x. 10.1111/j.1365-313X.2005.02548.x 16262707

[12] Chatfield SP, Stirnberg P, Forde BG, Leyser O. The hormonal regulation of axillary bud growth in Arabidopsis. The Plant Journal. 2000 10;24(2):159–69. Available from: http://www.ncbi.nlm.nih.gov/pubmed/11069691. 10.1046/j.1365-313x.2000.00862.x 11069691

[13] Laplaze L, Benková E, Casimiro I, Maes L, Vanneste S, Swarup R, et al Cytokinins act directly on lateral root founder cells to inhibit root initiation. The Plant Cell. 2007 12;19(12):3889–3900. Available from: http://www.plantcell.org/content/19/12/3889.abstract. 10.1105/tpc.107.055863 18065686 PMC2217640

[14] Takei K, Yamaya T, Sakakibara H. Arabidopsis CYP735A1 and CYP735A2 encode cytokinin hydroxylases that catalyze the biosynthesis of trans-Zeatin. The Journal of Biological Chemistry. 2004 10;279(40):41866–72. Available from: http://www.ncbi.nlm.nih.gov/pubmed/15280363. 10.1074/jbc.M406337200 15280363

[15] Miyawaki K, Matsumoto-Kitano M, Kakimoto T. Expression of cytokinin biosynthetic isopentenyltransferase genes in Arabidopsis: tissue specificity and regulation by auxin, cytokinin, and nitrate. The Plant Journal. 2004 1;37(1):128–138. Available from: 10.1046/j.1365-313X.2003.01945.x. 10.1046/j.1365-313X.2003.01945.x 14675438

[16] Jones B, Gunnerås SA, Petersson SV, Tarkowski P, Graham N, May S, et al Cytokinin regulation of auxin synthesis in Arabidopsis involves a homeostatic feedback loop regulated via auxin and cytokinin signal transduction. The Plant Cell. 2010 9;22(9):2956–2969. Available from: http://www.plantcell.org/content/22/9/2956.abstract. 10.1105/tpc.110.074856 20823193 PMC2965550

[17] Werner T, Köllmer I, Bartrina I, Holst K, Schmülling T. New insights into the biology of cytokinin degradation. Plant Biology. 2006 5;8:371–381. Available from: http://www.ncbi.nlm.nih.gov/pubmed/16807830. 10.1055/s-2006-923928 16807830

[18] Schlereth A, Möller B, Liu W, Kientz M, Flipse J, Rademacher EH, et al MONOPTEROS controls embryonic root initiation by regulating a mobile transcription factor. Nature. 2010 5;464(7290):913–916. Available from: 10.1038/nature08836. 10.1038/nature08836 20220754

[19] Müller B, Sheen J. Cytokinin and auxin interaction in root stem-cell specification during early embryogenesis. Nature. 2008 6;453(7198):1094–1097. Available from: http://www.nature.com/nature/journal/v453/n7198/suppinfo/nature06943_S1.html. 10.1038/nature06943 18463635 PMC2601652

[20] Taniguchi M, Sasaki N, Tsuge T, Aoyama T, Oka A. ARR1 directly activates cytokinin response genes that encode proteins with diverse regulatory functions. Plant and Cell Physiology. 2007 2;48(2):263–277. Available from: http://pcp.oxfordjournals.org/content/48/2/263.abstract. 10.1093/pcp/pcl063 17202182

[21] Růžička K, Šimášková M, Duclercq J, Petrášek J, Zažímalová E, Simon S, et al Cytokinin regulates root meristem activity via modulation of the polar auxin transport. Proceedings of the National Academy of Sciences of the United States of America. 2009 3;106(11):4284–9. Available from: http://www.pnas.org/content/106/11/4284.abstract. 10.1073/pnas.0900060106 19246387 PMC2657394

[22] Petrásek J, Mravec J, Bouchard R, Blakeslee JJ, Abas M, Seifertová D, et al PIN proteins perform a rate-limiting function in cellular auxin efflux. Science. 2006 5;312(5775):914–8. Available from: http://www.ncbi.nlm.nih.gov/pubmed/16601150. 10.1126/science.1123542 16601150

[23] Kramer EM, Bennett MJ. Auxin transport: a field in flux. Trends in Plant Science. 2006 8;11(8):382–6. Available from: http://www.ncbi.nlm.nih.gov/pubmed/16839804. 10.1016/j.tplants.2006.06.002 16839804

[24] Han X, Hyun TK, Zhang M, Kumar R, Koh EJ, Kang BH, et al Auxin-callose-mediated plasmodesmatal gating is essential for tropic auxin gradient formation and signaling. Developmental Cell. 2014 1;28(2):132–146. Available from: http://linkinghub.elsevier.com/retrieve/pii/S1534580713007351. 10.1016/j.devcel.2013.12.008 24480642

[25] Blakeslee JJ, Bandyopadhyay A, Lee OR, Mravec J, Titapiwatanakun B, Sauer M, et al Interactions among PIN-FORMED and P-glycoprotein auxin transporters in Arabidopsis. The Plant Cell. 2007 1;19(1):131–147. Available from: http://www.plantcell.org/cgi/doi/10.1105/tpc.106.040782. 10.1105/tpc.106.040782 17237354 PMC1820964

[26] Mravec J, Kubes M, Bielach A, Gaykova V, Petrásek J, Skůpa P, et al Interaction of PIN and PGP transport mechanisms in auxin distribution-dependent development. Development. 2008 10;135(20):3345–54. Available from: http://www.ncbi.nlm.nih.gov/pubmed/18787070. 10.1242/dev.021071 18787070

[27] Geisler M, Blakeslee JJ, Bouchard R, Lee OR, Vincenzetti V, Bandyopadhyay A, et al Cellular efflux of auxin catalyzed by the Arabidopsis MDR/PGP transporter AtPGP1. The Plant Journal. 2005 8;44(2):179–194. Available from: http://doi.wiley.com/10.1111/j.1365-313X.2005.02519.x. 10.1111/j.1365-313X.2005.02519.x 16212599

[28] Terasaka K. PGP4, an ATP Binding Cassette P-glycoprotein, catalyzes auxin transport in Arabidopsis thaliana roots. The Plant Cell. 2005 11;17(11):2922–2939. Available from: http://www.plantcell.org/cgi/doi/10.1105/tpc.105.035816. 10.1105/tpc.105.035816 16243904 PMC1276020

[29] Guo FQ, Wang R, Crawford NM. The Arabidopsis dual-affinity nitrate transporter gene AtNRT1.1 (CHL1) is regulated by auxin in both shoots and roots. Journal of Experimental Botany. 2002;53(370):835–844. Available from: http://jxb.oxfordjournals.org/content/53/370/835.short. 10.1093/jexbot/53.370.835 11912226

[30] Krouk G, Lacombe B, Bielach A, Perrine-Walker F, Malinska K, Mounier E, et al Nitrate-regulated auxin transport by NRT1.1 defines a mechanism for nutrient sensing in plants. Developmental Cell. 2010 6;18(6):927–937. Available from: http://linkinghub.elsevier.com/retrieve/pii/S1534580710002169. 10.1016/j.devcel.2010.05.008 20627075

[31] Grieneisen VA, Xu J, Marée AFM, Hogeweg P, Scheres B. Auxin transport is sufficient to generate a maximum and gradient guiding root growth. Nature. 2007 10;449(7165):1008–1013. Available from: http://www.nature.com/nature/journal/v449/n7165/full/nature06215.html. 10.1038/nature06215 17960234

[32] Blilou I, Xu J, Wildwater M, Willemsen V, Paponov I, Friml J, et al The PIN auxin efflux facilitator network controls growth and patterning in Arabidopsis roots. Nature. 2005 1;433(7021):39–44. Available from: http://www.nature.com/nature/journal/v433/n7021/full/nature03184.html. 10.1038/nature03184 15635403

[33] Wisniewska J, Xu J, Seifertová D, Brewer PB, Růžička K, Blilou I, et al Polar PIN localization directs auxin flow in plants. Science. 2006 5;312(5775):883 Available from: http://www.ncbi.nlm.nih.gov/pubmed/16601151. 10.1126/science.1121356 16601151

[34] Mähönen AP, Higuchi M, Törmäkangas K, Miyawaki K, Pischke MS, Sussman MR, et al Cytokinins regulate a bidirectional phosphorelay network in Arabidopsis. Current Biology. 2006 6;16(11):1116–22. Available from: http://www.ncbi.nlm.nih.gov/pubmed/16753566. 10.1016/j.cub.2006.04.030 16753566

[35] Mähönen AP, Bishopp A, Higuchi M, Nieminen KM, Kinoshita K, Tormakangas K, et al Cytokinin signaling and its inhibitor AHP6 regulate cell fate during vascular development. Science. 2006 1;311(5757):94–98. Available from: http://www.sciencemag.org/cgi/content/abstract/sci;311/5757/94. 10.1126/science.1118875 16400151

[36] Carlsbecker A, Lee JY, Roberts CJ, Dettmer J, Lehesranta S, Zhou J, et al Cell signalling by microRNA165/6 directs gene dose-dependent root cell fate. Nature. 2010 5;465(7296):316–21. Available from: http://www.ncbi.nlm.nih.gov/pubmed/20410882. 10.1038/nature08977 20410882 PMC2967782

[37] Ibañes M, Fàbregas N, Chory J, Caño Delgado AI. Brassinosteroid signaling and auxin transport are required to establish the periodic pattern of Arabidopsis shoot vascular bundles. Proceedings of the National Academy of Sciences of the United States of America. 2009 8;106(32):13630–13635. Available from: http://www.pnas.org/content/106/32/13630.abstract. 10.1073/pnas.0906416106 19666540 PMC2717112

[38] Chickarmane VS, Gordon SP, Tarr PT, Heisler MG, Meyerowitz EM. Cytokinin signaling as a positional cue for patterning the apical-basal axis of the growing Arabidopsis shoot meristem. Proceedings of the National Academy of Sciences of the United States of America. 2012 3;109(10):4002–4007. Available from: http://www.pnas.org/content/109/10/4002.abstract. 10.1073/pnas.1200636109 22345559 PMC3309735

[39] Laskowski M, Grieneisen VA, Hofhuis H, Hove CAT, Hogeweg P, Marée AFM, et al Root system architecture from coupling cell shape to auxin transport. PLoS Biology. 2008 12;6(12):e307 10.1371/journal.pbio.0060307 19090618 PMC2602721

[40] Laskowski M, Biller S, Stanley K, Kajstura T, Prusty R. Expression profiling of auxin-treated Arabidopsis roots: toward a molecular analysis of lateral root emergence. Plant and Cell Physiology. 2006 6;47(6):788–92. Available from: http://www.ncbi.nlm.nih.gov/pubmed/16621846. 10.1093/pcp/pcj043 16621846

[41] De Rybel B, Adibi M, Breda AS, Wendrich JR, Smit ME, Novak O, et al Integration of growth and patterning during vascular tissue formation in Arabidopsis. Science. 2014 8;345(6197):1255215–1255215. Available from: http://www.sciencemag.org/cgi/doi/10.1126/science.1255215. 10.1126/science.1255215 25104393

[42] Peaceman DW, Rachford HH Jr. The numerical solution of parabolic and elliptic differential equations. Journal of the Society for Industrial and Applied Mathematics. 1955 3;3(1):28–41. 10.1137/0103003

[43] Swarup R, Kargul J, Marchant A, Zadik D, Rahman A, Mills R, et al Structure-function analysis of the presumptive Arabidopsis auxin permease AUX1. The Plant Cell. 2004;16:3069–3083. 10.1105/tpc.104.024737 15486104 PMC527199

[44] Bainbridge K, Guyomarc’h S, Bayer E, Swarup R, Bennett M, Mandel T, et al Auxin influx carriers stabilize phyllotactic patterning. Genes & Development. 2008 3;22(6):810–23. Available from: http://www.pubmedcentral.nih.gov/articlerender.fcgi?artid=2275433&tool=pmcentrez&rendertype=abstract. 10.1101/gad.462608 18347099 PMC2275433

[45] Marchant A, Bennett MJ. The Arabidopsis AUX1 gene: a model system to study mRNA processing in plants. Plant Molecular Biology. 1998 2;36(3):463–71. Available from: http://www.ncbi.nlm.nih.gov/pubmed/9484486. 10.1023/A:1005961303167 9484486

[46] Swarup K, Benková E, Swarup R, Casimiro I, Péret B, Yang Y, et al The auxin influx carrier LAX3 promotes lateral root emergence. Nature Cell Biology. 2008 8;10(8):946–54. Available from: http://www.ncbi.nlm.nih.gov/pubmed/18622388. 10.1038/ncb1754 18622388

[47] Band LR, Wells DM, Fozard JA, Ghetiu T, French AP, Pound MP, et al Systems analysis of auxin transport in the Arabidopsis root apex. The Plant Cell. 2014 3;26(3):862–875. Available from: http://www.plantcell.org/cgi/doi/10.1105/tpc.113.119495. 10.1105/tpc.113.119495 24632533 PMC4001398

[48] Péret B, Swarup K, Ferguson A, Seth M, Yang Y, Dhondt S, et al AUX/LAX genes encode a family of auxin influx transporters that perform distinct functions during Arabidopsis development. The Plant Cell. 2012 7;24(7):2874–85. Available from: http://www.ncbi.nlm.nih.gov/pubmed/22773749. 10.1105/tpc.112.097766 22773749 PMC3426120

[49] Fàbregas N, Formosa-Jordan P, Confraria A, Siligato R, Alonso JM, Swarup R, et al Auxin influx carriers control vascular paterning and xylem differentiation in Arabidopsis thaliana. PLoS Genetics. 2015;11(4):e1005183 Available from: http://journals.plos.org/plosgenetics/article?id=10.1371/journal.pgen.1005183. 10.1371/journal.pgen.1005183 25922946 PMC4414528

[50] Willemsen V, Wolkenfelt H, de Vrieze G, Weisbeek P, Scheres B. The HOBBIT gene is required for formation of the root meristem in the Arabidopsis embryo. Development. 1998 2;125(3):521–31. Available from: http://www.ncbi.nlm.nih.gov/pubmed/9425146. 9425146 10.1242/dev.125.3.521

[51] Müller A, Guan C, Gälweiler L, Tänzler P, Huijser P, Marchant A, et al AtPIN2 defines a locus of Arabidopsis for root gravitropism control. The EMBO Journal. 1998;17(23):6903–6911. Available from: http://www.nature.com/emboj/journal/v17/n23/abs/7591380a.html. 10.1093/emboj/17.23.6903 9843496 PMC1171038

[52] Bishopp A, Lehesranta S, Vatén A, Help H, El-Showk S, Scheres B, et al Phloem-transported cytokinin regulates polar auxin transport and maintains vascular pattern in the root meristem. Current Biology. 2011 6;21(11):927–932. Available from: http://www.sciencedirect.com/science/article/pii/S096098221100491X. 10.1016/j.cub.2011.04.049 21620705

[53] Kramer EM. How far can a molecule of weak acid travel in the apoplast or xylem? Plant Physiology. 2006 8;141(4):1233–1236. Available from: 10.1104/pp.106.083790. 10.1104/pp.106.083790 16896235 PMC1533949

[54] Grieneisen VA, Scheres B, Hogeweg P, Marée AFM. Morphogengineering roots: comparing mechanisms of morphogen gradient formation. BMC Systems Biology. 2012 1;6:37 Available from: http://www.ncbi.nlm.nih.gov/pubmed/22583698. 10.1186/1752-0509-6-37 22583698 PMC3681314

[55] De Rybel B, Möller B, Yoshida S, Grabowicz I, Barbier de Reuille P, Boeren S, et al A bHLH complex controls embryonic vascular tissue establishment and indeterminate growth in Arabidopsis. Developmental Cell. 2013 24 1–12. Available from: http://www.ncbi.nlm.nih.gov/pubmed/23415953. 23415953 10.1016/j.devcel.2012.12.013

[56] Kudo T, Kiba T, Sakakibara H. Metabolism and long-distance translocation of cytokinins. Journal of Integrative Plant Biology. 2010 1;52(1):53–60. Available from: 10.1111/j.1744-7909.2010.00898.x. 10.1111/j.1744-7909.2010.00898.x 20074140

[57] Zurcher E, Tavor-Deslex D, Lituiev D, Enkerli K, Tarr PT, Muller B. A robust and sensitive synthetic sensor to monitor the transcriptional output of the cytokining signaling network in planta. Plant Physiology. 2013 1;161(3):1066–1075. Available from: http://www.plantphysiol.org/cgi/doi/10.1104/pp.112.211763. 10.1104/pp.112.211763 23355633 PMC3585579

[58] De Smet I, Tetsumura T, De Rybel B, Frey NFD, Laplaze L, Casimiro I, et al Auxin-dependent regulation of lateral root positioning in the basal meristem of Arabidopsis. Development. 2007 2;134(4):681–90. Available from: http://www.ncbi.nlm.nih.gov/pubmed/17215297. 10.1242/dev.02753 17215297

[59] Van Norman JM, Xuan W, Beeckman T, Benfey PN. To branch or not to branch: the role of pre-patterning in lateral root formation. Development. 2013 11;140(21):4301–4310. Available from: http://dev.biologists.org/cgi/doi/10.1242/dev.090548. 10.1242/dev.090548 24130327 PMC4007709

[60] Dubrovsky JG, Sauer M, Napsucialy-Mendivil S, Ivanchenko MG, Friml J, Shishkova S, et al Auxin acts as a local morphogenetic trigger to specify lateral root founder cells. Proceedings of the National Academy of Sciences of the United States of America. 2008 6;105(25):8790–4. Available from: http://www.pubmedcentral.nih.gov/articlerender.fcgi?artid=2438385&tool=pmcentrez&rendertype=abstract. 10.1073/pnas.0712307105 18559858 PMC2438385

[61] Dubrovsky JG, Gambetta GA, Hernández-Barrera A, Shishkova S, González I. Lateral root initiation in Arabidopsis: developmental window, spatial patterning, density and predictability. Annals of botany. 2006 5;97(5):903–15. Available from: http://www.pubmedcentral.nih.gov/articlerender.fcgi?artid=2803408&tool=pmcentrez&rendertype=abstract. 10.1093/aob/mcj604 16390845 PMC2803408

[62] Novoselova ES, Mironova VV, Omelyanchuk NA, Kazantsev FV, Likhoshvai VA. Mathematical modeling of auxin transport in protoxylem and protophloem of Arabidopsis thaliana root tips. Journal of Bioinformatics and Computational Biology. 2013 2;11(1). Available from: http://www.ncbi.nlm.nih.gov/pubmed/23427992. 10.1142/S0219720013400106 23427992

[63] Lucas M, Godin C, Jay-Allemand C, Laplaze L. Auxin fluxes in the root apex co-regulate gravitropism and lateral root initiation. Journal of Experimental Botany. 2008 1;59(1):55–66. Available from: http://www.ncbi.nlm.nih.gov/pubmed/17720688. 10.1093/jxb/erm171 17720688

[64] Band LR, King JR. Multiscale modelling of auxin transport in the plant-root elongation zone. Journal of Mathematical biology. 2012 10;65(4):743–85. Available from: http://www.ncbi.nlm.nih.gov/pubmed/22015980. 10.1007/s00285-011-0472-y 22015980

[65] Mähönen AP, ten Tusscher K, Siligato R, Smetana O, Díaz-Triviño S, Salojärvi J, et al PLETHORA gradient formation mechanism separates auxin responses. Nature. 2014 8;515(7525):125–129. Available from: http://www.nature.com/doifinder/10.1038/nature13663. 10.1038/nature13663 25156253 PMC4326657

[66] Barbosa ICR, Zourelidou M, Willige BC, Weller B, Schwechheimer C. D6 PROTEIN KINASE activates auxin transport-dependent growth and PIN-FORMED phosphorylation at the plasma membrane. Developmental Cell. 2014 6;29(6):674–685. Available from: http://linkinghub.elsevier.com/retrieve/pii/S1534580714003086. 10.1016/j.devcel.2014.05.006 24930721

[67] Zhang W, Swarup R, Bennett M, Schaller GE, Kieber JJ. Cytokinin induces cell division in the quiescent center of the Arabidopsis root apical meristem. Current Biology. 2013 10;23(20):1979–1989. Available from: http://linkinghub.elsevier.com/retrieve/pii/S0960982213009846. 10.1016/j.cub.2013.08.008 24120642

