# Supplementary figures and images for: Parsimonious Model of Vascular Patterning Links Transverse Hormone Fluxes to Lateral Root Initiation: Auxin Leads the Way, while Cytokinin Levels Out
Source: PLoS Comput Biol. 2015 Oct 27;11(10):e1004450. doi: 10.1371/journal.pcbi.1004450 (PMC4623515; doi:10.1371/journal.pcbi.1004450)

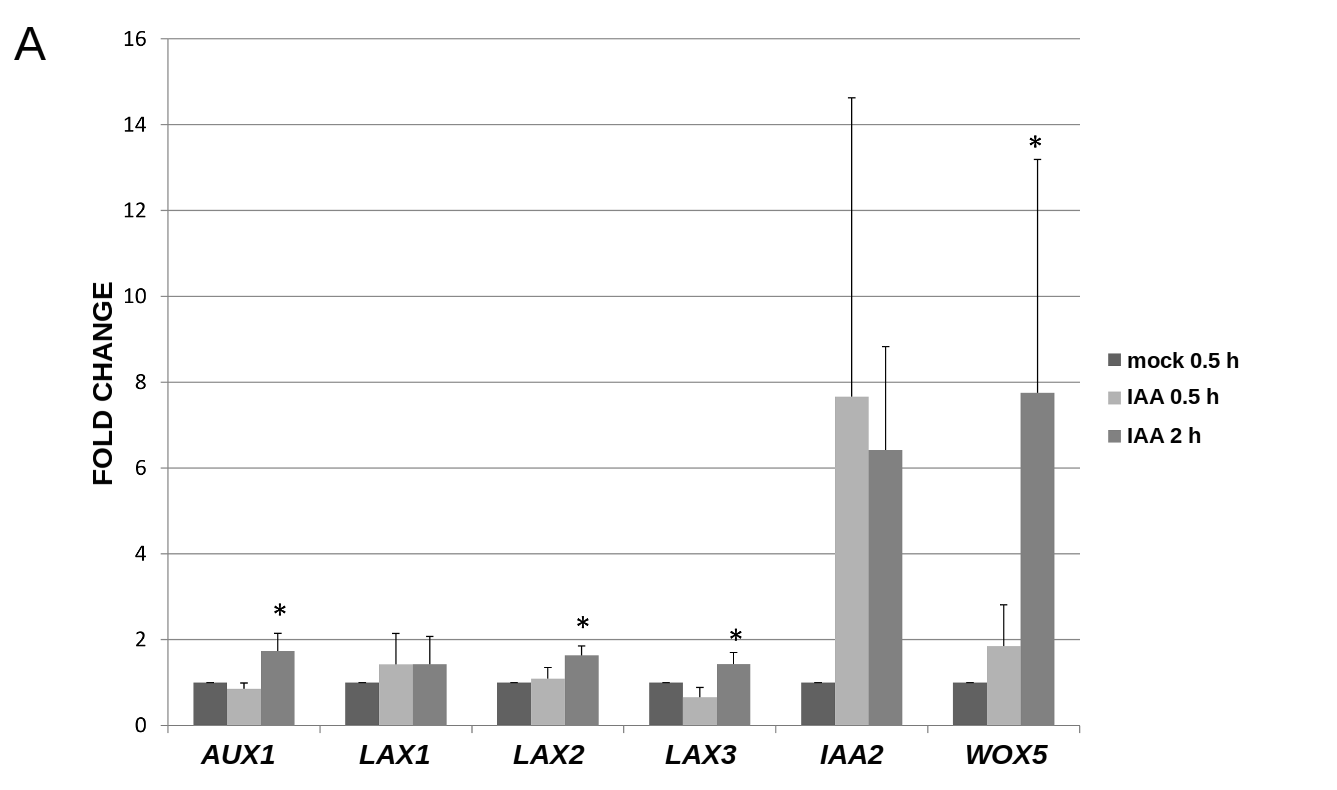

Supplement: S1 Fig — A qRT-PCR of the auxin importers following treatments with 1 μm IAA shows modest increase in expression in response to auxin. Error bars depict standard deviation; statistically significant differences in gene expression (p < 0.05) in IAA-treated roots in comparison to the respective mock are marked with asterisks. (TIF) [file pcbi.1004450.s001.tif]

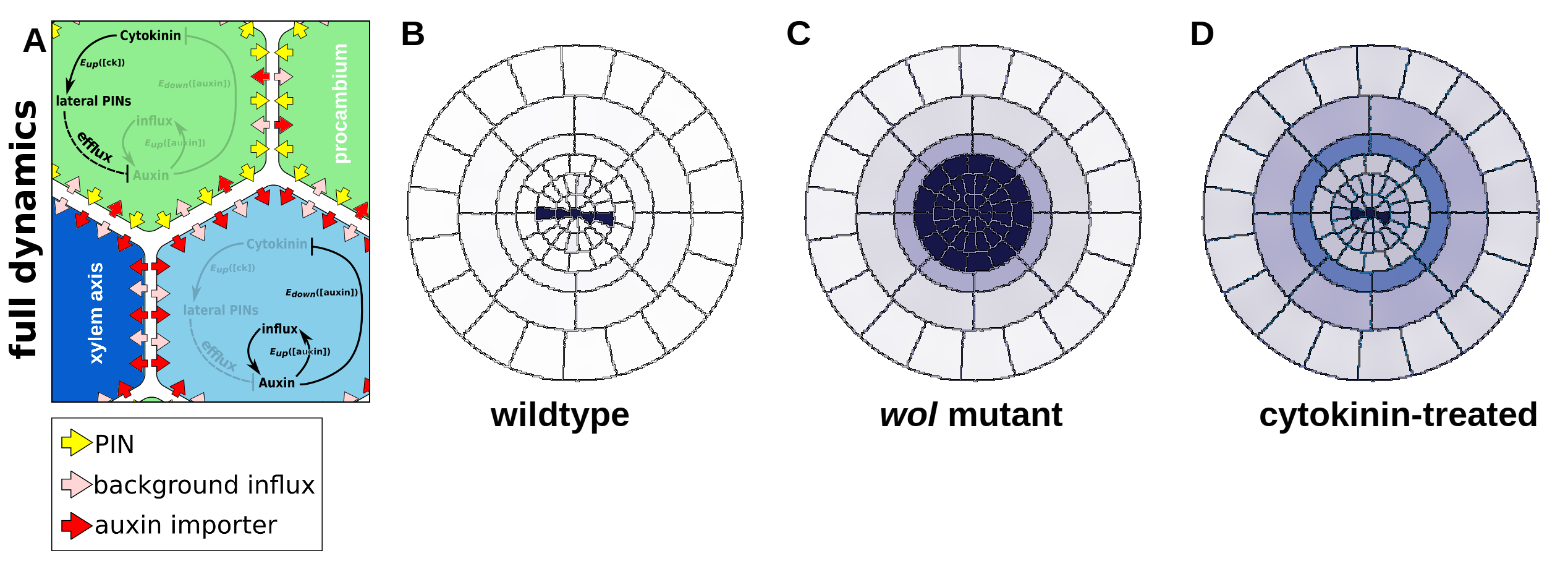

Supplement: S2 Fig — The ‘DR5-like’ output from simulations with (A) hormonal regulation of the PINs and auxin-regulation of the importer in (B) wild type, (C) wol, and (D) cytokinin-treated geometric root sections. (TIF) [file pcbi.1004450.s002.tif]

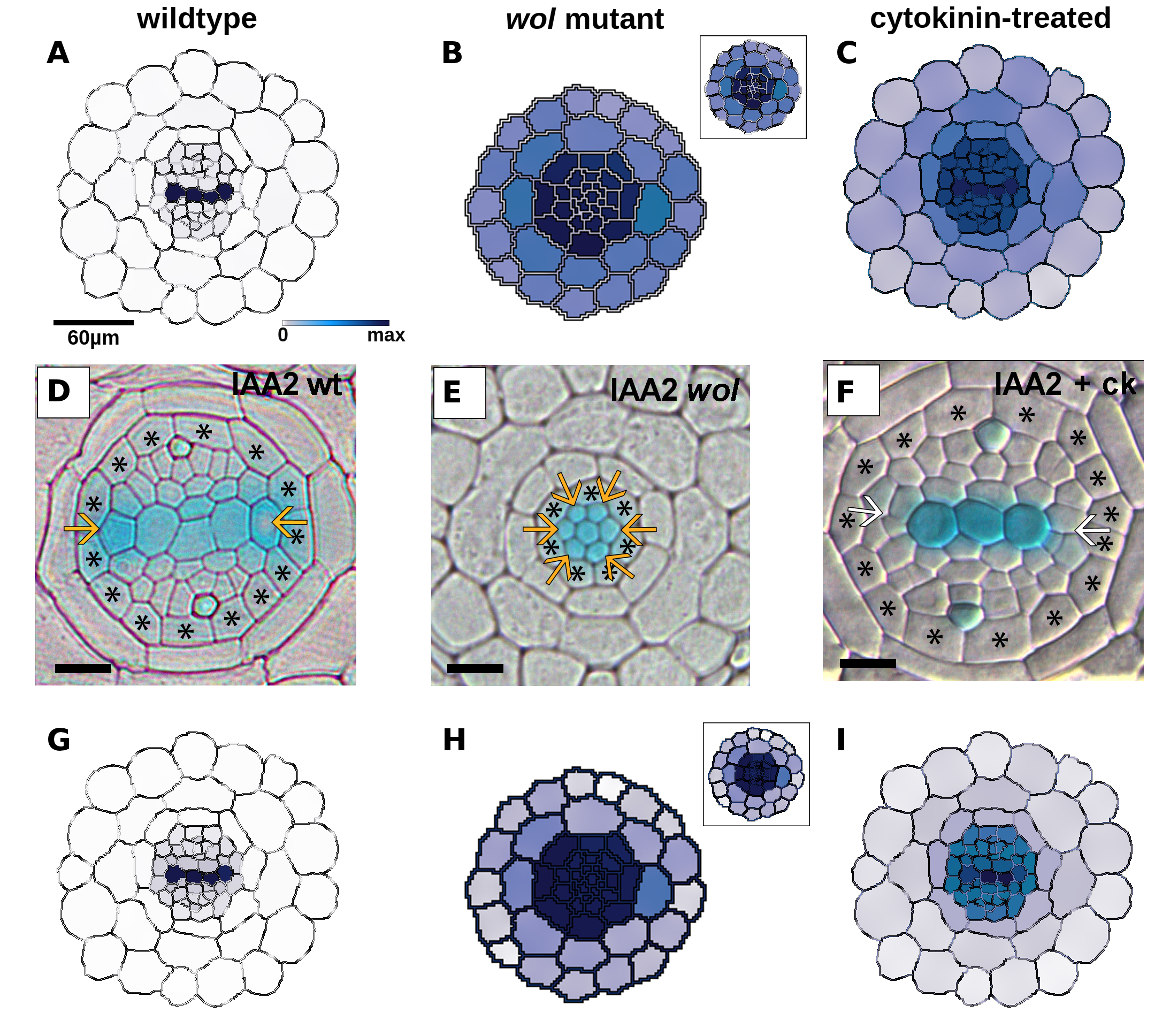

Supplement: S3 Fig — The ‘DR5-like’ output from simulations of a realistic root with unregulated PIN expression (A–C) do not in all cases generate the observed auxin signalling pattern (D–F). (G–I) Simulations in which the PINs are regulated correspond more closely to the experimental data. (A, D, G) Wild-type roots; (B, E, H) wol; (C, F, I) cytokinin-treated roots. Insets show the wol roots at the same scale as others; the larger wol figures are scaled 250% to improve visibility. (TIF) [file pcbi.1004450.s003.tif]

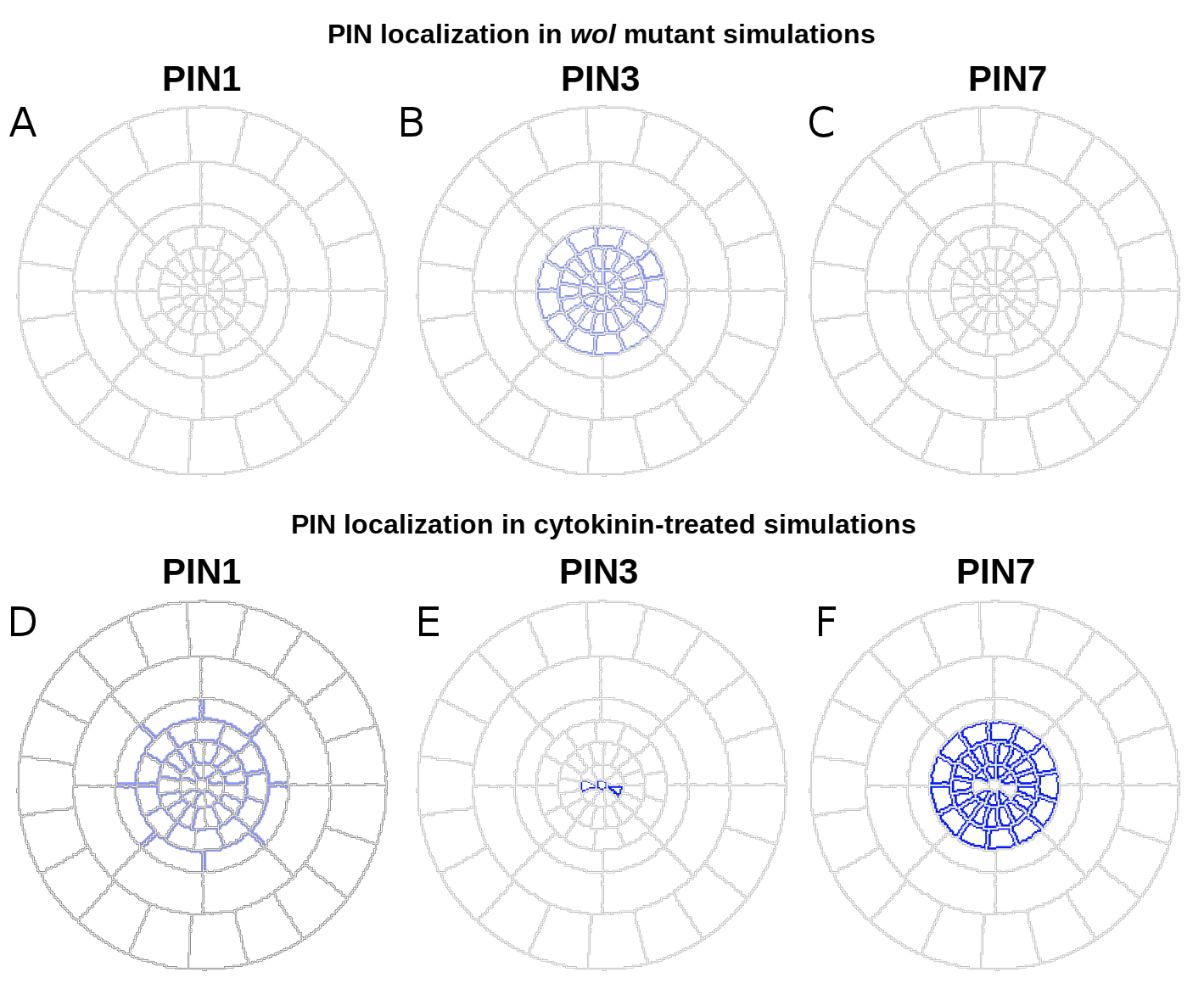

Supplement: S4 Fig — (A–C) Localisation of (A) PIN1; (B) PIN3; and (C) PIN7, according to experimental observations in wol. (D–F) Localisation of (D) PIN1; (E) PIN3; and (F) PIN7, according to experimental observations in wild-type roots treated with cytokinin. Localisations are based on observations reported in [3]; lighter colour indicates a lower maximum expression level. (TIF) [file pcbi.1004450.s004.tif]

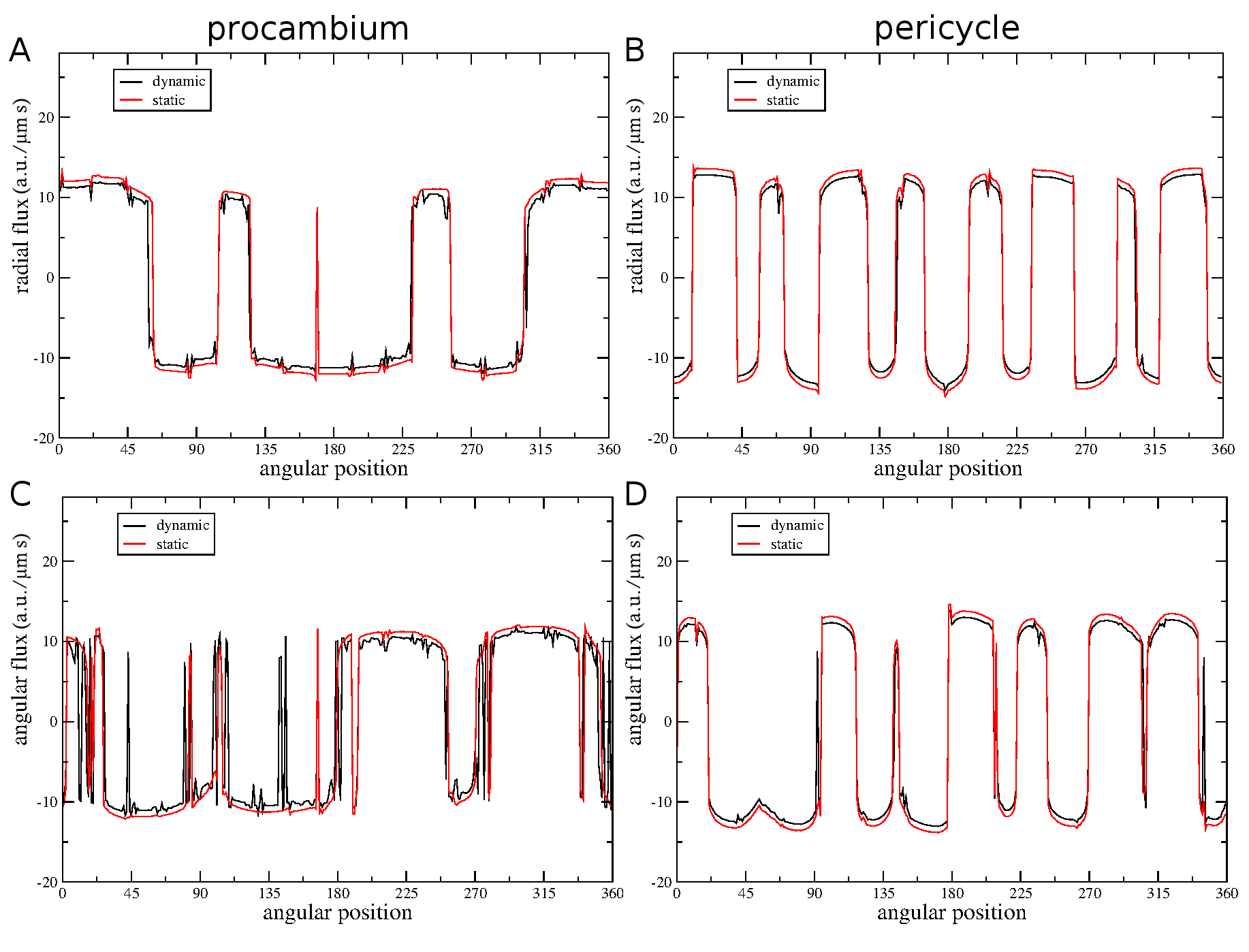

Supplement: S5 Fig — (A, C) Graphs depicting the radial (A) and angular (C) fluxes in the procambium of static (black) and dynamic (red) simulations of wol. The fluxes are plotted as a function of the angular position along the circumference of the root from 0°–360°. (B, D) Graphs depicting the radial (B) and angular (D) fluxes in the pericycle of static (black) and dynamic (red) simulations of wol. Fluxes are again plotted as a function of the angular position. (TIF) [file pcbi.1004450.s005.tif]

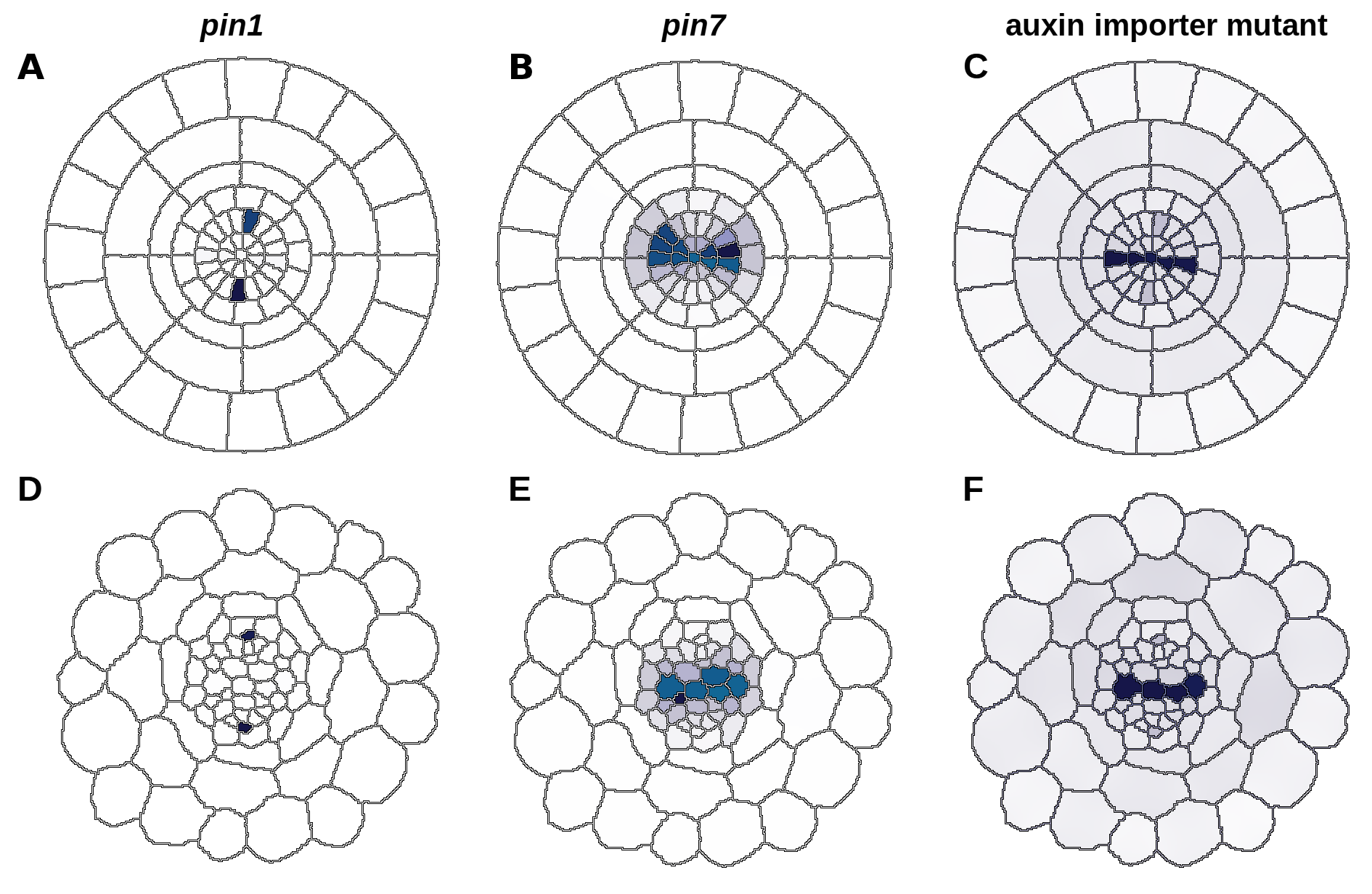

Supplement: S6 Fig — The simulated DR5 pattern in geometric (A–C) and realistic (D–F) roots of (A, D) pin1, (B, E) pin7, and (C, F) auxin importer mutants. (TIF) [file pcbi.1004450.s006.tif]

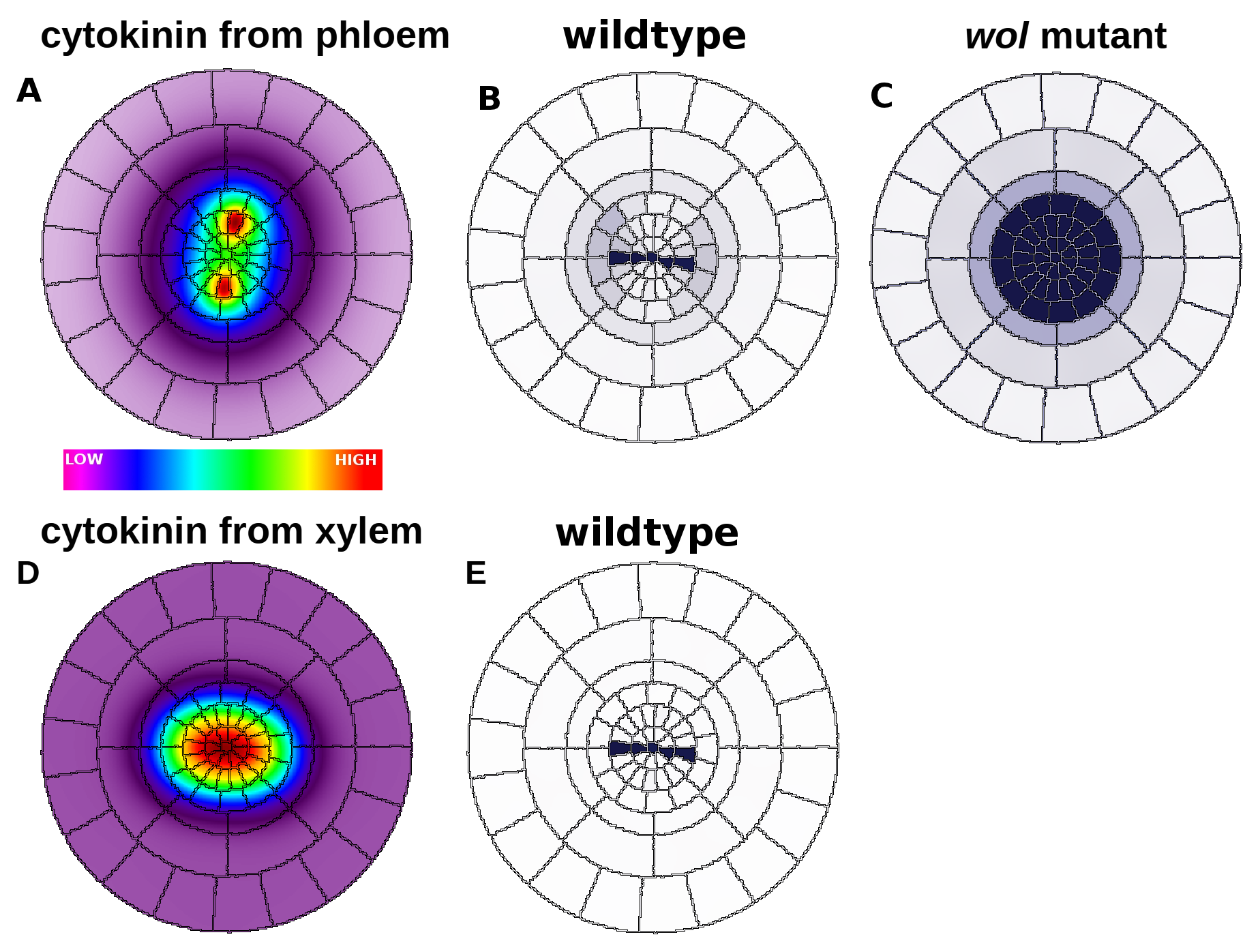

Supplement: S7 Fig — (A) Heatmap of cytokinin distribution in a steep gradient centred around the phloem poles. ‘DR5-like’ output from simulations of (B) wild-type and (C) wol roots produce the same pattern as with a flat cytokinin distribution. (D) Heatmap of a cytokinin gradient formed by synthesis in the xylem axis. (E) Simulation of a wild-type root produces the same auxin pattern as with a flat cytokinin distribution. (TIF) [file pcbi.1004450.s007.tif]

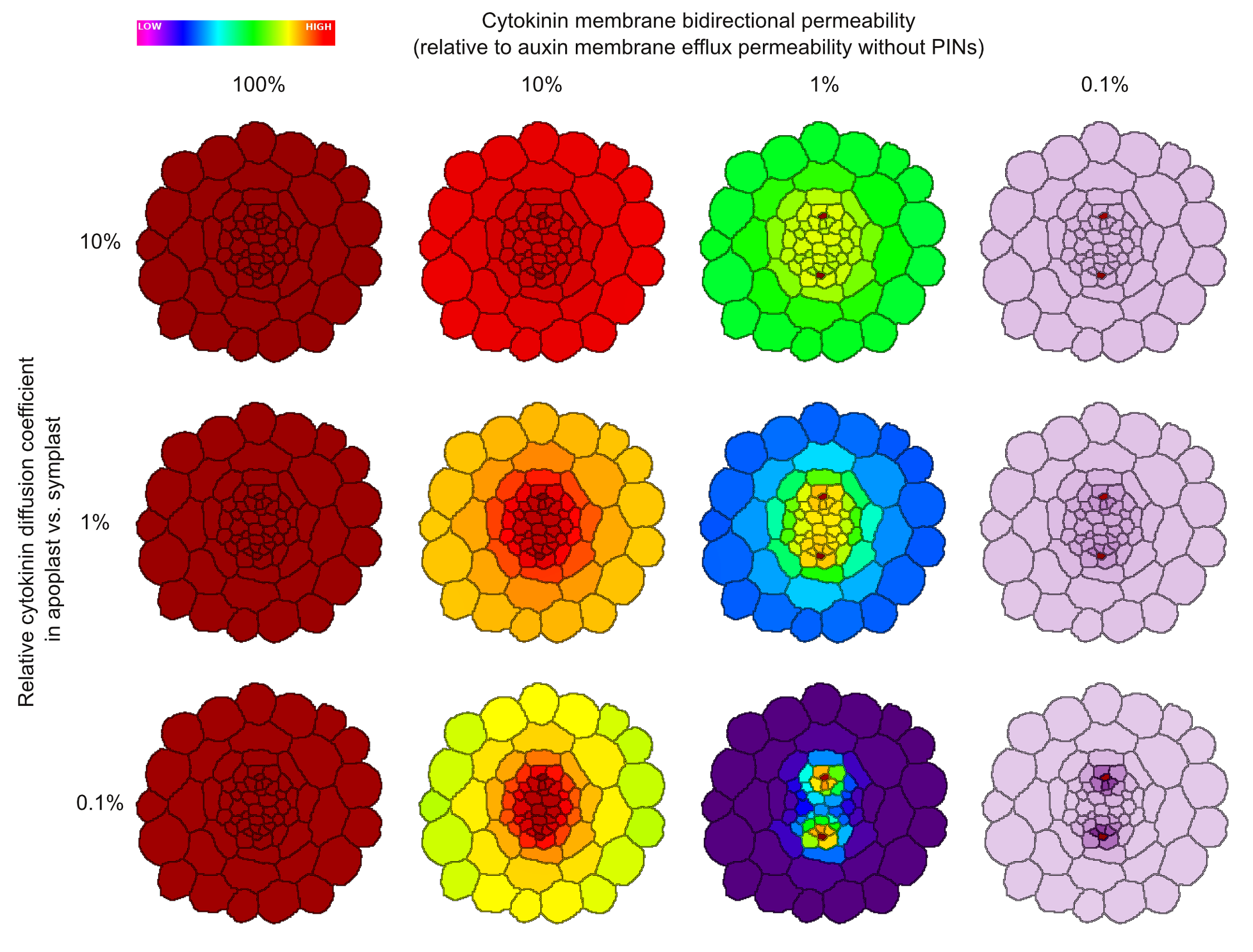

Supplement: S8 Fig — To establish a cytokinin gradient without altering the diffusion coefficient of cytokinin, we introduced bidirectional membrane permeability. Decreasing the membrane permeability (moving right within rows) concentrates cytokinin in the source cells (here, the phloem poles), but a gradient is not established because the diffusion within the apoplast evens out the distribution within the rest of the root. Decreasing the diffusion coefficient (downwards in columns) in the apoplast transforms the step-wise cytokinin distribution pattern into a true gradient. Parameter values are given in Table 6. (TIF) [file pcbi.1004450.s008.tif]

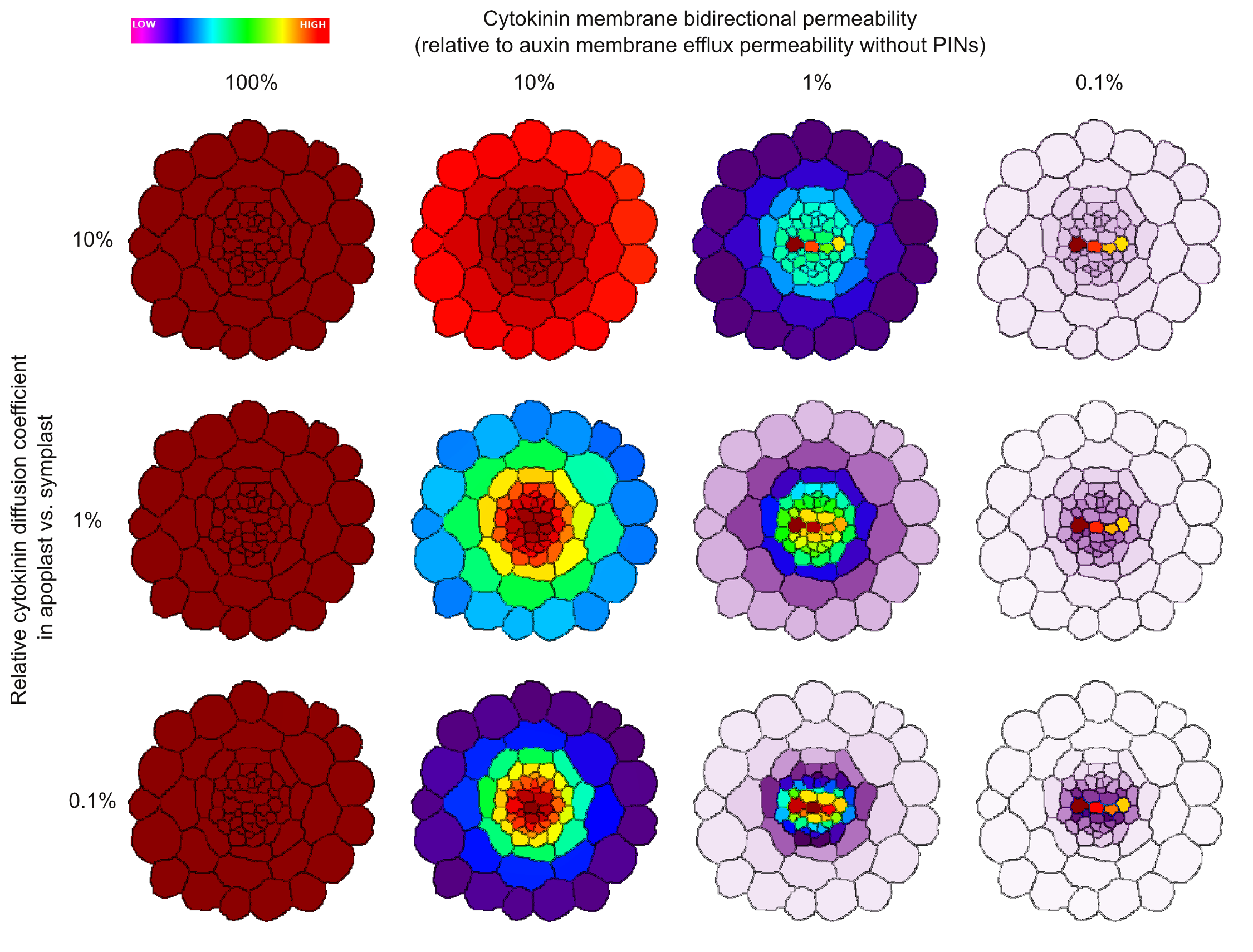

Supplement: S9 Fig — All cells in the cross section can synthesize cytokinin in an auxin-dependent manner. As in the simulations with fixed cytokinin biosynthesis (Fig 7, S8 Fig), decreasing the membrane permeability (moving right within rows) does not establish a gradient because the diffusion within the apoplast evens out the distribution within the rest of the root. Decreasing the diffusion coefficient within the apoplast (moving downwards within the columns) transforms the step-wise cytokinin distribution pattern into a true gradient. Parameter values are given in Table 6 and Table 4. (TIF) [file pcbi.1004450.s009.tif]

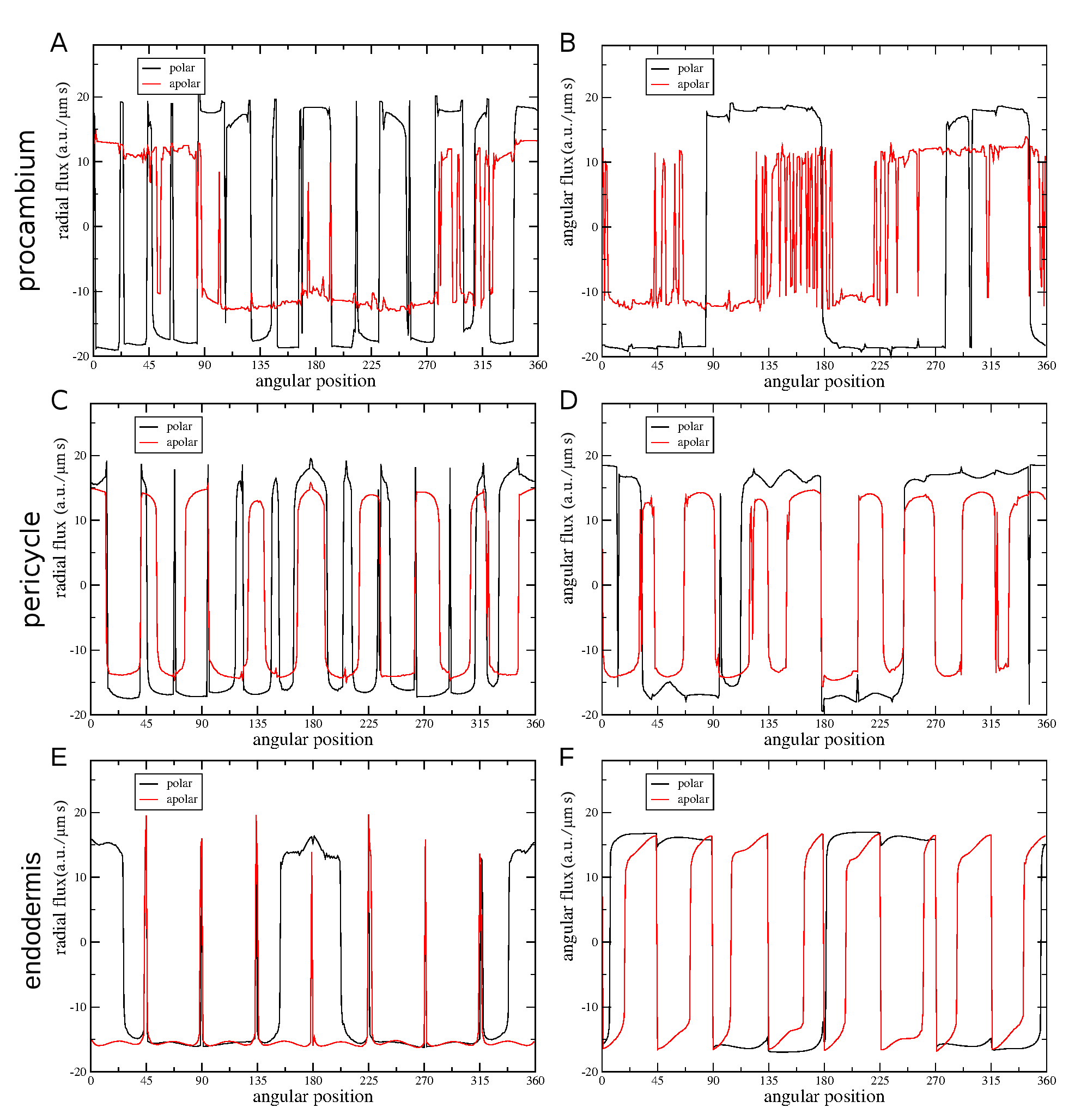

Supplement: S10 Fig — Graphs depicting radial (A, C, E) and angular (B, D, F) auxin fluxes in simulations with apolar (red) and polar (red) PIN1 localisation. The fluxes are plotted as a function of the angular position along the circumference of the root from 0° − 360°. (A, B) Radial (A) and angular (B) fluxes in the procambium. (C, D) Radial (C) and angular (D) fluxes in the pericycle. (E, F) Radial (E) and angular (F) fluxes in the endodermis. (TIF) [file pcbi.1004450.s010.tif]

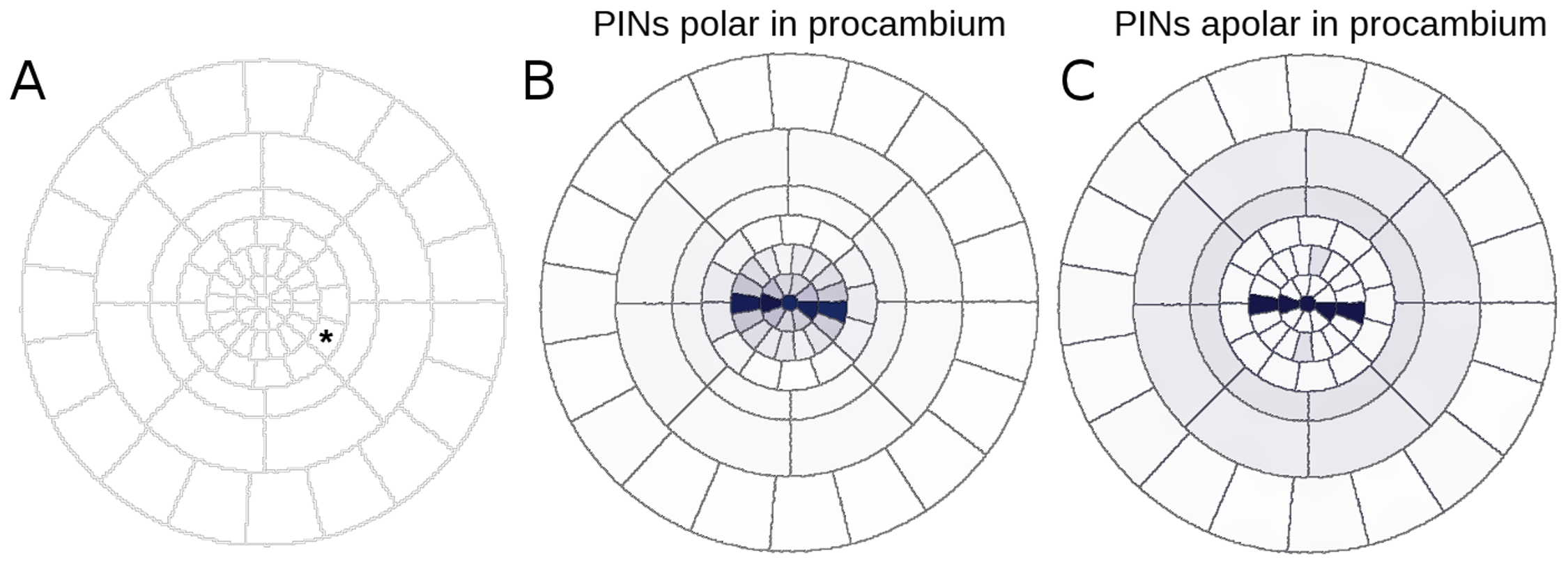

Supplement: S11 Fig — ‘DR5-like’ output of simulations in which PIN1 and PIN7 are apolar in the pericycle and a focal cell receives an AUX1 pulse. (A) The cell receiving the AUX1 pulse. The resulting auxin pattern when PIN1 and PIN7 are (B) polar and (C) apolar in the procambium. (TIF) [file pcbi.1004450.s011.tif]

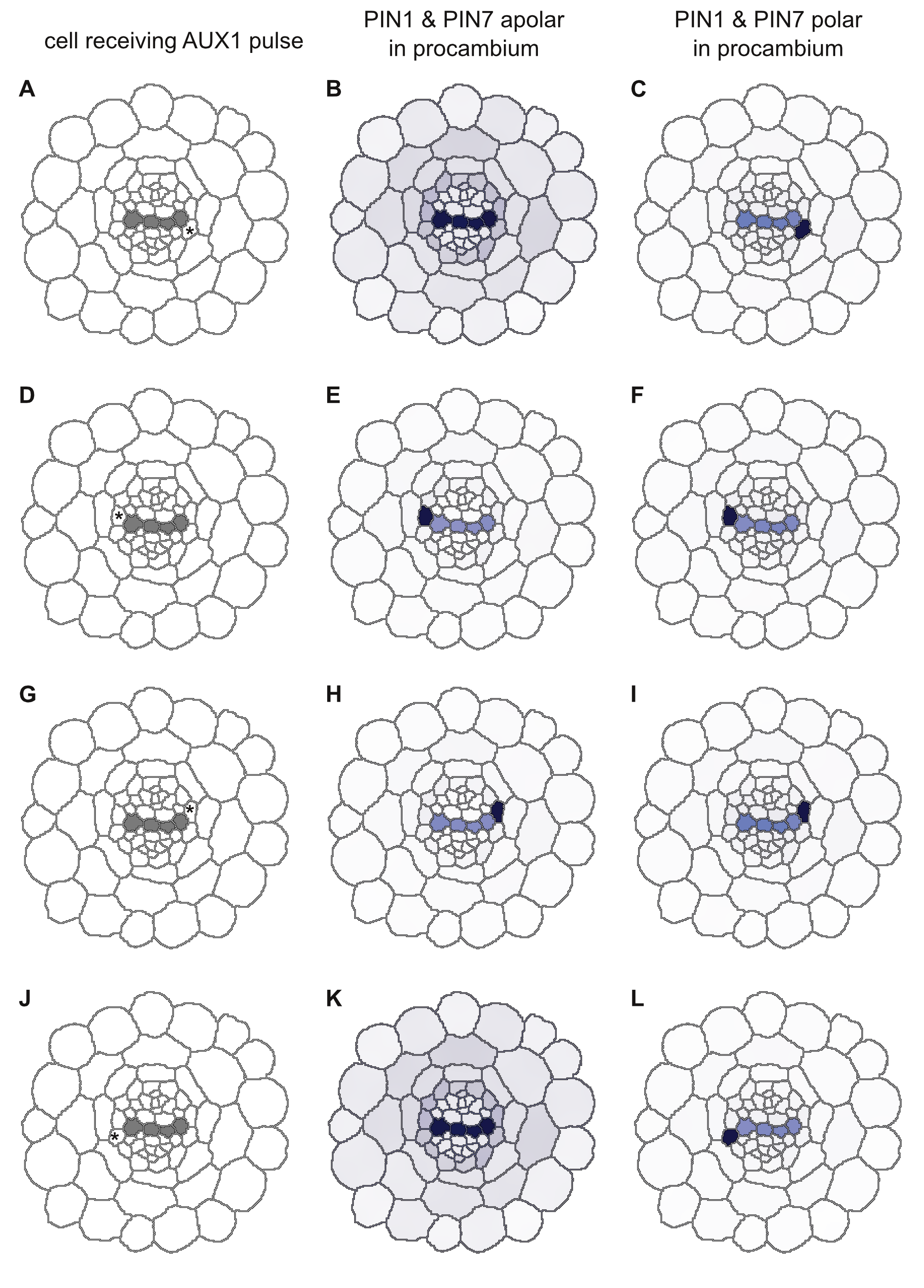

Supplement: S12 Fig — A 120-second pulse of AUX1 activation was provided to the pericycle cell marked with an asterisk (A, D, G, J). In simulations with apolar PIN1 and PIN7, only two of the xylem-pole pericycle cells could maintain AUX1 activity and accumulate auxin (B, E, H, K). By contrast, the pulse caused persistent activation in any xylem-pole pericycle cell in simulations with polar PIN1 and PIN7 (C, F, I, L). Auxin levels are depicted using a ‘DR5-like’ scale. (TIF) [file pcbi.1004450.s012.tif]

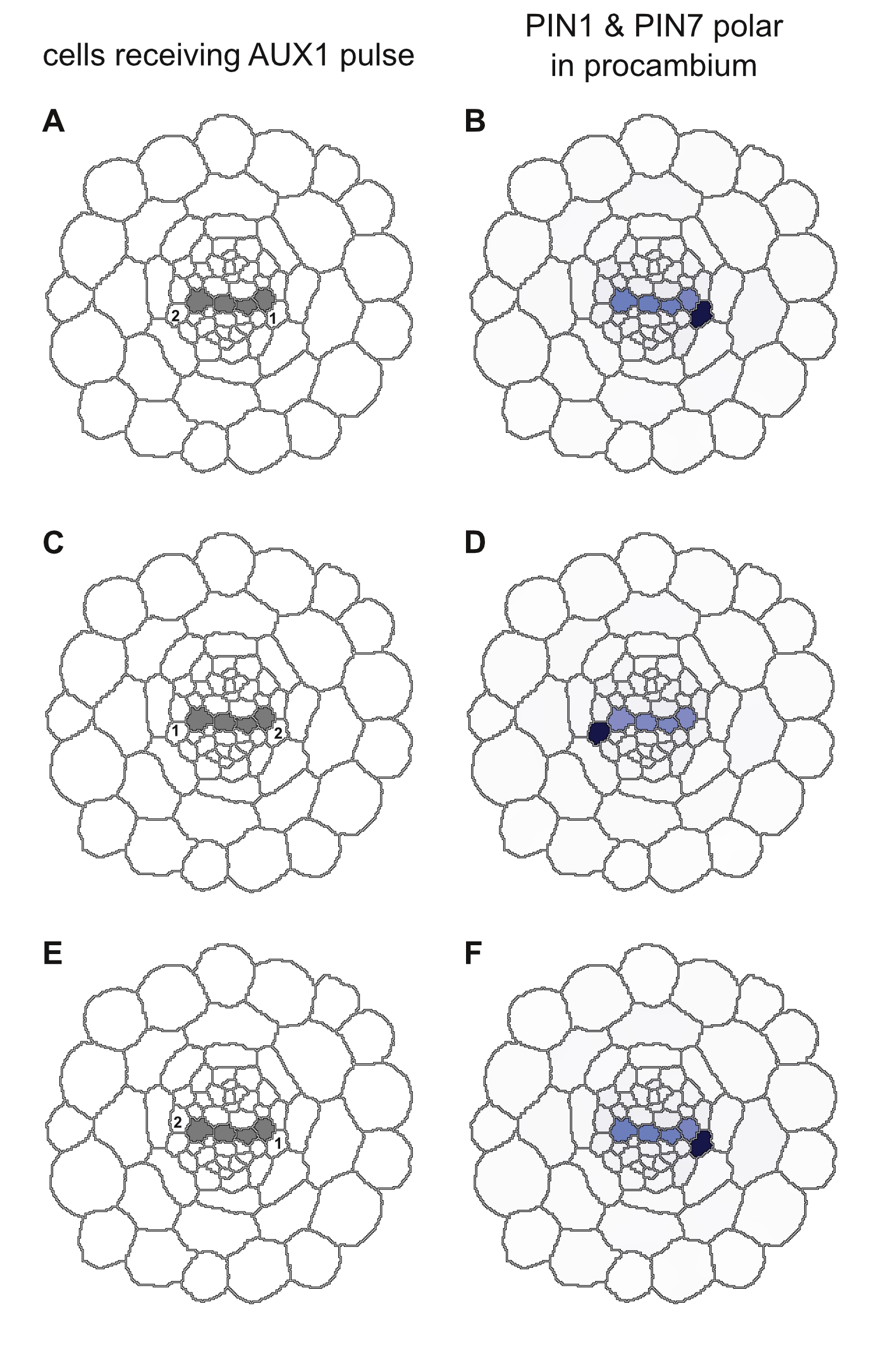

Supplement: S13 Fig — A 120-second pulse of AUX1 activation was provided to two pericycle cells with a five second delay between the cells marked 1 and 2 (A, C, E). The first cell to receive the AUX1 pulse retains AUX1 activation and accumulates auxin (B, D, F). Auxin levels are depicted using a ‘DR5-like’ scale. (TIF) [file pcbi.1004450.s013.tif]

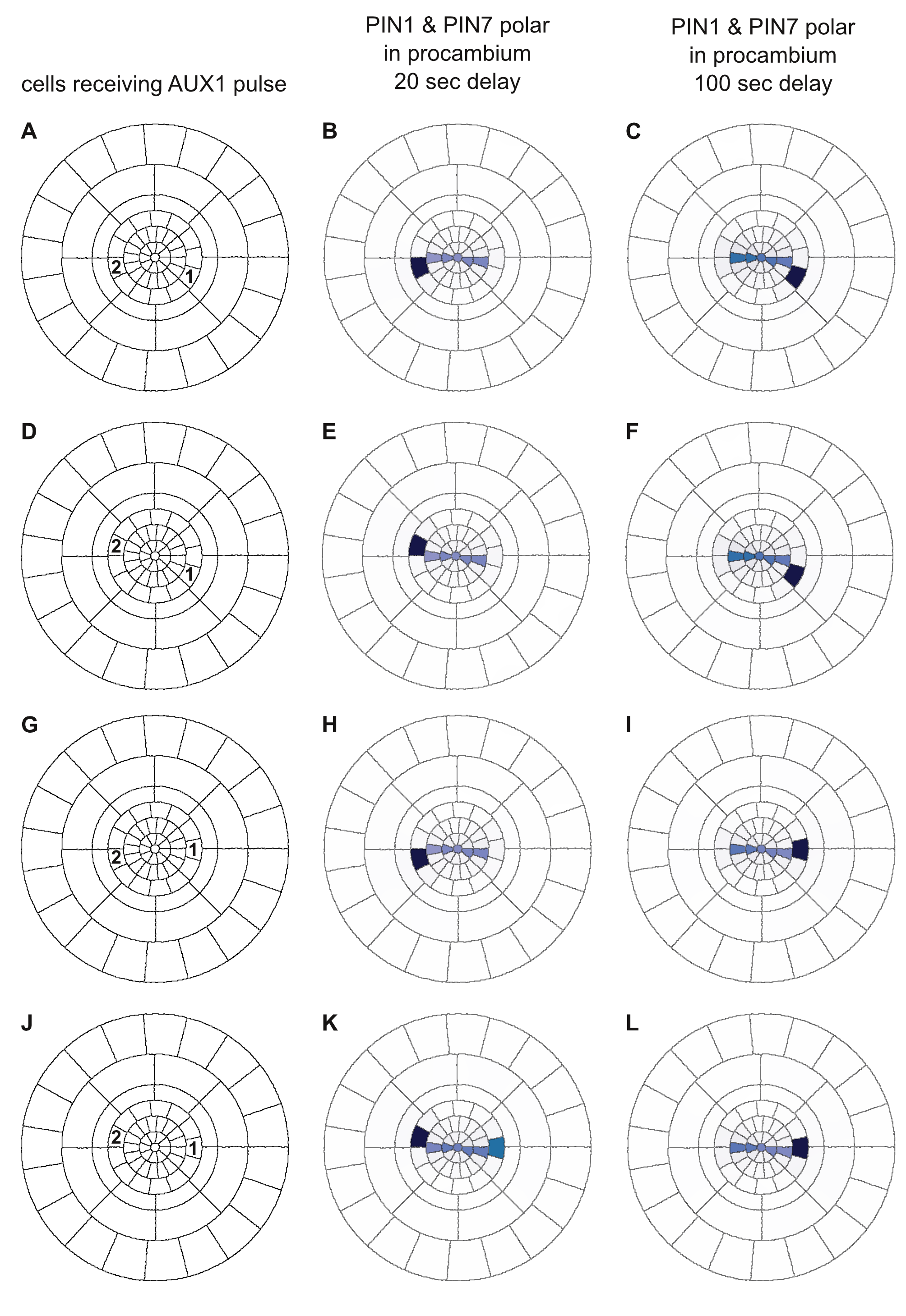

Supplement: S14 Fig — A 120-second pulse of AUX1 activation was provided to two pericycle cells with a 20 s or 100 s delay between the cells marked 1 and 2 (A, D, G, J). Following a 20 s delay, the identity of the cell accumulating auxin is the same as when the pulse is simultaneous (B, E, H, K), but with a longer delay of 100 s the cell receiving the earlier pulse accumulates auxin (C, F, I, L). Auxin levels are depicted using a ‘DR5-like’ scale. (TIF) [file pcbi.1004450.s014.tif]
